# Modulating Calcium Homeostasis via a Biomimetic Scaffold to Rescue Diabetic Ischemic Wounds

**DOI:** 10.1002/advs.76758

**Published:** 2026-07-27

**Authors:** Xiang Zheng, Mingmei Li, Jianjun Jiang, Yongchao Wang, Yaru Jia, Weilun Sun, Yi Yao, Pengli Gao, Linhua Zhang, Qi Guo, Guang Jia, Xing‐Jie Liang, Dunwan Zhu, Jinchao Zhang, Fangzhou Li

**Affiliations:** ^1^ College of Chemistry *&* Materials Science Key Laboratory of Medicinal Chemistry and Molecular Diagnosis of Ministry of Education State Key Laboratory of New Pharmaceutical Preparations and Excipients Chemical Biology Key Laboratory of Hebei Province Hebei University Baoding People's Republic of China; ^2^ State Key Laboratory of Advanced Medical Materials and Devices Tianjin Key Laboratory of Biomedical Materials Key Laboratory of Biomaterials and Nanotechnology for Cancer Immunotherapy Institute of Biomedical Engineering Tianjin Institutes of Health Science Chinese Academy of Medical Sciences *&* Peking Union Medical College Tianjin People's Republic of China; ^3^ School of Life Sciences Zhengzhou University Zhengzhou People's Republic of China; ^4^ CAS Key laboratory for Biomedical effects of nanomaterials and nanosafety CAScenter for excellence in nanoscience National Center for Nanoscience and Technology of China Beijing People's Republic of China; ^5^ State Key Laboratory of Natural Medicines Department of Pharmaceutics School of Pharmacy China Pharmaceutical University Nanjing People's Republic of China; ^6^ University of Chinese Academy of Sciences Beijing People's Republic of China

**Keywords:** diabetic wound healing, extracellular matrix, immune microenvironment, nanofibrous scaffold, perfusion

## Abstract

Diabetic wound healing is critically impaired by a pathological microenvironment with two core barriers: circulatory deficits from microangiopathy, and a loss of nanoscale topographical guidance due to extracellular matrix (ECM) disruption. To address this, we developed Musc@CP, an advanced dressing that synergistically combines an ECM‐mimetic multi‐property scaffold with active vascular modulation. The platform is based on a chitosan‐pullulan (CP) nanofibrous scaffold that structurally recapitulates healthy dermal ECM to direct fibroblast adhesion and migration. The matrix incorporates muscone (Musc), a bioactive compound that attenuates intracellular Ca^2+^ overload‐associated endothelial dysfunction and is associated with improved local perfusion. This restoration of blood circulation may contribute to reprogramming the immune microenvironment within the wound, helping to suppress both inflammation and oxidative stress. Consequently, the healing progression in diabetic mice is effectively restored. Together, this integrated strategy represents a promising and mechanistically sound therapy for chronic diabetic wounds.

## Graphical abstract

This strategy addresses impaired microcirculation and loss of extracellular matrix (ECM) guidance in diabetic wound healing. Musc@CP, a nanofibrous dressing combining an ECM‐mimetic chitosan‐pullulan scaffold with muscone, enhances perfusion by attenuating intracellular Ca^2+^ overload‐associated endothelial dysfunction. The resulting increase in blood flow is associated with an antiinflammatory immune shift. Together, these synergistic effects accelerate wound healing in diabetic mice.

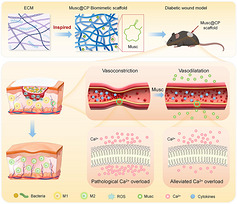

## 1 Introduction

Chronic non‐healing wounds, driven by the global surge in diabetes, pose a significant clinical crisis. Among them, diabetic foot ulcers (DFUs) are a major contributor to non‐traumatic amputations [1, 2]. The failure to heal stems from a self‐reinforcing pathological loop involving two key elements: ischemic conditions due to microangiopathy and the structural collapse of the native extracellular matrix (ECM). This ECM breakdown, specifically at the nanoscale, deprives cells of the topographical signals needed for directed migration [3, 4]. Concurrently, insufficient perfusion creates a deficit of oxygen, nutrients, and crucial molecular signals [5]. Trapped by this dual burden, the wound microenvironment cannot escape a chronic inflammatory state, thus halting the normal progression to the proliferation phase [6, 7].

Modern wound care has moved toward advanced dressings that provide a structural scaffold for tissue regrowth [8, 9]. However, a critical and often overlooked limitation lies in their physical structure. Many widely used commercial dressings are composed of microfibers [10, 11]. While these materials can absorb exudate and provide a protective cover, their coarse, micron‐scale topography creates a fundamental mismatch with the cellular environment [12]. As evidenced by electron microscopy, their micron‐scale features fail to emulate the fine topographical landscape that cells naturally encounter, thereby lacking the necessary contact guidance to orchestrate cell alignment, migration, and tissue reorganization [13, 14]. The absence of such biomimetic physical signals, combined with the persistent ischemic milieu, is considered to contribute to the disordered and incomplete repair seen in diabetic wounds [15].

Here, we engineer a quaternized chitosan‐based wound dressing featuring a genuine nanofibrous architecture that mimics the dimensions of native ECM fiber. This scaffold provides potential topographical guidance as part of a multi‐faceted biomimetic design, addressing a key limitation of current micrometer‐scale wound products. Furthermore, we integrated muscone into the nanofibrous system to combat the impaired microcirculation characteristic of diabetic wounds. This natural compound is recognized in traditional medicine for its ability to promote perfusion and resolve stasis [16, 17]. Clinically, muscone serves as a key functional ingredient in established formulations such as Mayinglong Muscone Hemorrhoid Ointment, where it contributes to swelling reduction and pain relief. Through this integrated strategy combining comprehensive physicochemical optimization with biochemical modulation, we demonstrate in a diabetic mouse model that our multi‐functional platform acts synergistically with muscone's biochemical activity to dramatically outperform conventional microfiber dressings. Our work establishes that replicating the ECM‐mimetic microenvironment via multi‐property integration, combined with muscone's ability to promote perfusion, constitutes a critical design principle for the next generation of highly effective diabetic wound therapies.

## 2 Results and Discussion

### 2.1 Structural and Performance Analysis of ECM‐Mimetic Musc@CP

**FIGURE 1:**
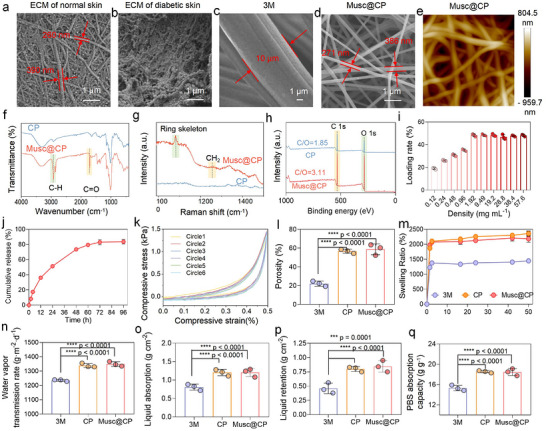
Preparation and Characterization of Musc@CP. SEM images of (a) the ECM of normal mouse skin, (b) the ECM of diabetic mouse skin, (c) 3M brand commercial dressings, and (d) Musc@CP scaffold. (e) AFM image of Musc@CP scaffold. (f) FTIR spectrum and (g) Raman spectroscopy of Musc@CP with characteristic peaks labeled. (h) High‐resolution XPS spectra of the C1s and O1s regions, along with the C/O ratio. (i) Loading rate of Musc on CP scaffold. (j) Cumulative release of muscone from the Musc@CP scaffold at pH 8. (k) Stress–strain curves of Musc@CP. (l) Porosity and (m) swelling kinetics (ratio of mass increase to initial mass vs. time) of scaffolds. (n) Water vapor transmission rate of scaffolds. (o) Water absorption and (p) water retention capacity of scaffolds after 1 day immersion in water (Data are presented as mean values ± s.d. (q) Absorption capacity of scaffolds toward PBS. (n = 3 independent experiments).

The native skin ECM exhibits a highly fibrous [18, 19], three‐dimensional architecture composed of regularly aligned nanoscale fibers that provide crucial structural support and contact guidance to cells (Figure 1a). This organized structure presents a stark contrast to the disorganized ECM fiber arrangement observed in diabetic mouse tissues (Figure 1b and Figure S1). Commercial wound dressings, including the representative 3M product shown in Figure 1c, feature fiber diameters exceeding 10 µm, which are substantially larger than natural ECM nanofibers and consequently fail to replicate their contact guidance functions [20, 21].

Inspired by the nanofibrous architecture of native ECM, we developed a biomimetic quaternized chitosan‐pullulan (CP) scaffold through electrospinning (Figure S2). The introduction of pullulan into the chitosan system significantly improved spinnability by counteracting the inherent rigidity of pure chitosan that normally hinders uniform nanofiber formation [22, 23]. Through systematic optimization of key electrospinning parameters such as solution concentration, spinning distance, C/P ratio, and applied voltage, followed by a controlled foaming process, we fabricated a series of three‐dimensional porous nanofiber scaffolds (Figures S3 and S4 and Table S1). Among these, CP3 emerged as the optimal candidate based on critical structural parameters including pore size (58.7%), fiber diameter (379 ± 119 nm), and specific surface area (298.5 m^2^ g^−1^) (Figure S5). This selected formulation closely mimics the native ECM, featuring nanoscale fibers and a well‐defined three‐dimensional architecture (Figure 1d). The ECM‐mimetic CP scaffolds are expected to provide favorable contact guidance and enhance directional cell migration through their specialized physical traits, representing distinct microenvironmental advantages over conventional microfiber dressings such as 3M products [13, 14, 15].

To evaluate whether the ECM‐mimetic CP scaffold can emulate the regulatory functions of native extracellular matrix in wound healing, we systematically investigated its effects on primary human dermal fibroblast behaviors, with particular focus on cellular activation, migration and proliferation. When L929 fibroblasts were cultured on CP scaffold, they exhibited distinct morphological features of activation, including the prominent extension of filopodia and lamellipodia at the cell periphery. These structural changes were not observed in the control or 3M dressing groups (Extended Data Figure S1a). Consistent with these morphological alterations, expression of Fibroblast Activation Protein (FAP) was significantly higher in CP compared to the control and 3M groups (Extended Data Figure S1b). We next investigated two critical functional outcomes of fibroblast activation: migratory capacity and proliferative potential [24]. In scratch wound healing assays, L929 cells cultured with CP scaffold demonstrated substantially improved wound closure under standard culture conditions (37°C, 5% CO_2_), achieving 54.1% closure within 24 h. This migratory performance significantly surpassed that of the control (26.9%) and 3M groups (27.1%) (Extended Data Figure S1c,d), indicating enhanced directional migration into the wound gap when guided by the ECM‐mimetic scaffolds. To determine whether our scaffold expanded the pool of cells for tissue repair, we measured the proliferation of 3T3 fibroblasts. The CP scaffold markedly increased fibroblast expansion (Extended Data Figure S1e,f), providing a substantial cellular basis for the subsequent development of robust granulation tissue. These results demonstrate that the CP scaffold, designed to mimic ECM, promotes pro‐migratory and pro‐proliferative responses.

To fabricate a more bioactive scaffold, muscone (Musc) was incorporated into the CP matrix, yielding the Musc@CP composite. Muscone, a principal active component of Mayinglong Hemorrhoid Ointment, is recognized for its ability to promote perfusion [25]. Structural analysis revealed that the muscone loading process did not alter the scaffold's integrity. The atomic force microscope (AFM) (Figure 1e and Figure S6) images showed a preserved morphological structure. The successful incorporation was further confirmed by spectroscopic data: FTIR spectra displayed characteristic C─H and C═O stretching vibrations (Figure 1f), Raman spectroscopy identified CH_2_ groups (Figure 1g), and XPS analysis indicated a substantially increased C/O ratio in Musc@CP compared to pure CP (Figure 1h and Table S2). These results collectively confirm that muscone was successfully integrated into the fibrous matrix.

Musc@CP composite material exhibits excellent drug properties, with a drug loading capacity as high as 48.24% (Figure 1i). At pH 8, 80% of the drug was continuously released within 72 h (Figure 1j), while at pH 7.4, 60% sustained release was also achieved (Figure S7). Therefore, Musc@CP exhibits favorable release behavior (Figure S7). Stress‐strain curves confirmed its compressive resilience (Figure 1k and Figure S8), a key mechanical property for wound support. In direct comparison with a commercial 3M dressing, our scaffold presented superior porosity (58.7%, Figure 1l), swelling ratio (2180%, Figure 1m), and hydrophilicity (Figure S9). Its fluid management performance was advanced, featuring a water vapor transmission rate of 1350 g m^−2^ d^−1^ (Figure 1n). The scaffold absorbed liquid at 1.23 g cm^−2^ (Figure 1o) and retained 0.85 g cm^−2^ (Figure 1p), with PBS and blood absorption values recorded at 18.5 and 10.4 g g^−1^, respectively (Figure 1q and Figure S10). A hemolysis rate of only 4.5% (Figure S11) indicated excellent hemocompatibility and a promising safety profile. This combination of efficient drug carriage, controlled release, robust mechanics, balanced fluid control, and blood compatibility addresses the fundamental requirements for an ideal dressing in diabetic wound care.

While these findings highlight the advantages of our platform, the direct comparison with the 3M dressing inherently involves distinct material parameters such as chemical composition, porosity, surface charge, and hydrophilicity. Therefore, the specific bio‐receptive contribution of the nanoscale topography has not been completely isolated from other physicochemical traits in this study, which warrants systematically decoupled investigations utilizing compositionally identical but morphologically distinct substrates in future work.

### 2.2 The Enhanced Perfusion, Anti‐Inflammatory, and Anti‐Oxidative Stress Properties of Musc@CP

**FIGURE 2:**
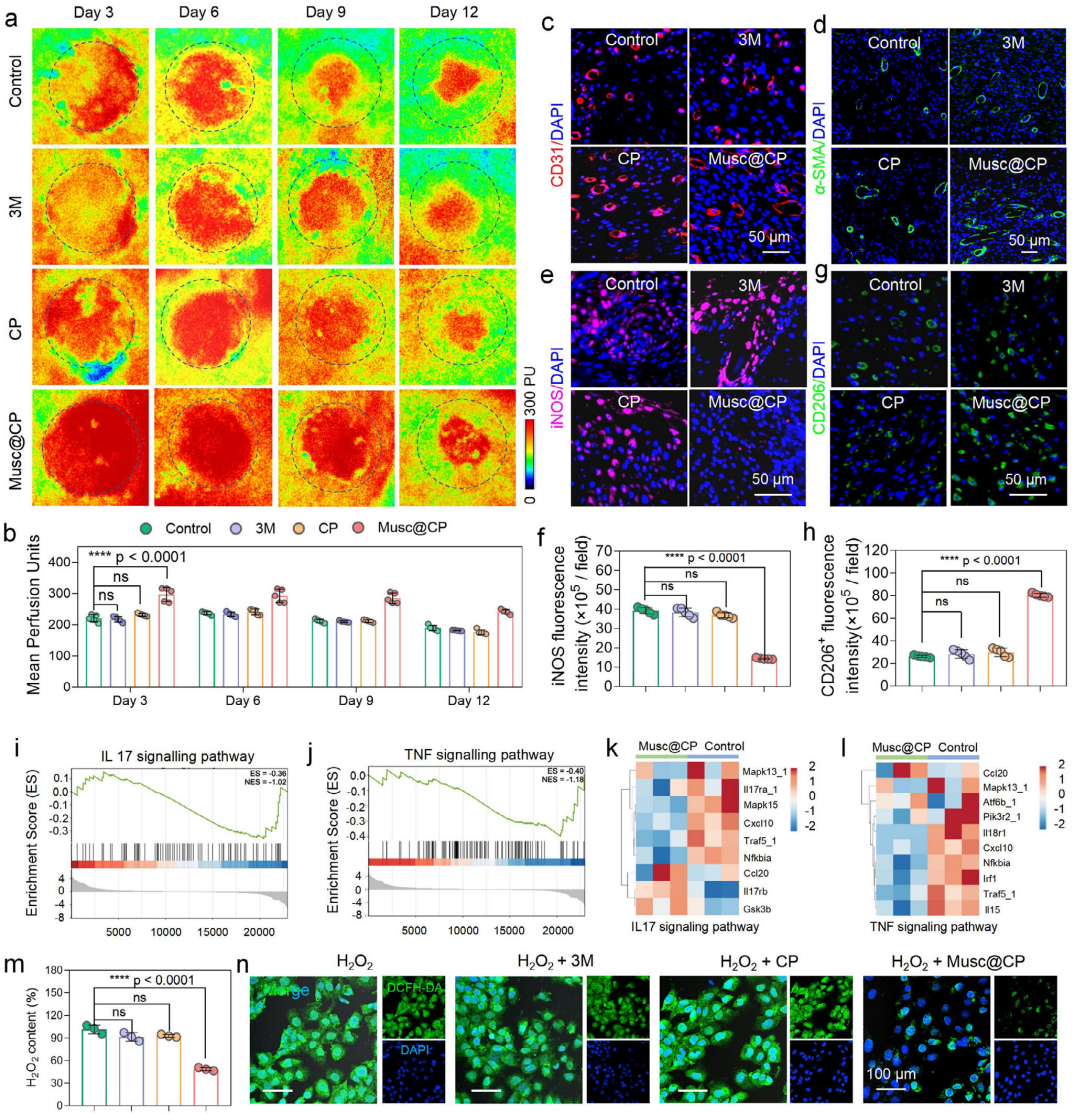
The enhanced perfusion, anti‐inflammatory, and anti‐oxidative stress properties of Musc@CP. (a) Spatial map of blood perfusion in different in vivo treatment groups on day 3, day 6, day 9 and day 12. (b) PU of ROI (Region of Interest) in different treatment groups on day 3, day 6, day 9 and day 12. Relative immunofluorescence staining images of (c) CD31, (d) *α*‐SMA, (e) iNOS, and (g) CD206 in different in vivo treatment groups on day 3. The fluorenscence intensity of (f) iNOS and (h) CD206 in different in vivo treatment groups on day 3. (n = 5 independent cells). (i,j) GSEA plots for the IL‐17 and TNF signaling pathways. (k,l) Heatmaps of DEGs involved in KEGG pathways (IL‐17 signaling pathway, TNF signaling pathway). (m) H_2_O_2_ scavenging performance in response to various treatments. The data are presented as the mean ± s.d. (n) ROS scavenging ability of scaffolds in L929. Each experiment was repeated independently three times with similar results.

The restoration of microcirculation is critical for wound repair, as it delivers oxygen, nutrients, and cytokines and eliminates metabolic waste [26, 27], thereby directly reducing tissue hypoxia and inflammation to accelerate healing [28, 29], In our study, laser Doppler imaging demonstrated that Musc@CP treatment most effectively enhanced wound perfusion in diabetic mice, achieving an average level of 300 Perfusion Units (PU), significantly higher than controls (Figure 2a,b). Consistent with this functional improvement, immunofluorescence staining revealed that Musc@CP also promoted vascular remodeling, increasing vessel diameter and maturity, as indicated by CD31 and *α*‐SMA markers (Figure 2c,d and Figure S12a,b). This revitalized circulation modulated the local immune landscape [30, 31]. On post‐wounding day 3, the Musc@CP group showed minimal iNOS expression (Figure 2e,f) and low TNF‐*α* levels (Figure S13a), alongside maximal CD206 intensity (Figure 2g,h) and elevated IL‐4 (Figure S13b), collectively indicating a transition toward anti‐inflammatory responses [32, 33]. Notably, relative to the Control group, the Musc+CP group (CP scaffold plus free muscone) exhibited a marginal reduction in iNOS expression and a slight elevation in CD206 expression (Figure S14). Nevertheless, both of these indicators were significantly inferior to those observed in the Musc@CP group. These results suggest that simply admixing muscone with the CP scaffold, while showing a certain trend, is substantially less potent in suppressing inflammation and promoting M2 polarization compared with Musc@CP, which enables nanofiber based sustained release of muscone.

To determine whether this M2 polarization resulted directly from the CP scaffold/muscone or indirectly from improved perfusion, we performed in vitro assays. Co‐culture of macrophages with CP scaffold, free muscone, or Musc@CP did not significantly promote M1 to M2 transition, as assessed by flow cytometry and ELISA for inflammatory cytokines (Figure S15), indicating minimal direct immunomodulation by the scaffold or muscone alone. Therefore, the observed in vivo M2 shift is likely an indirect consequence of enhanced perfusion.

To further decipher the overarching regulatory mechanism in vivo, we performed RNA‐seq. Kyoto Encyclopedia of Genes and Genomes (KEGG) enrichment analysis of the data highlighted significant changes in specific inflammatory pathways, including TNF and IL‐17 signaling (Figures S16–S18). Gene Set Enrichment Analysis (GSEA) confirmed downregulation of both pathways (Figure 2i,j), characterized by reduced expression of *Il17ra* and *Il18r1* (Figure 2k,l), indicating suppression of inflammatory signaling [34, 35]. This anti‐inflammatory profile was further supported by upregulated Arginase‐1 expression (Figure S19). Thus, the restored blood circulation is associated with the reprogramming of the local immune landscape toward a reparative phenotype, which in turn correlates with the resolution of chronic inflammation in diabetic wounds. We note that while our in vitro data argue against a dominant direct immunomodulatory effect, we cannot completely rule out minor contributions from the scaffold's topography or muscone; nevertheless, the preponderance of evidence supports a correlation between perfusion restoration and anti‐inflammatory immune changes.

Given the critical role of oxidative stress in impaired diabetic healing [36], we next evaluated the anti‐oxidant capacity of Musc@CP. The scaffold demonstrated significantly enhanced reactive oxygen species (ROS) scavenging ability compared to both 3M and CP controls across multiple concentrations (Figure 2m and Figure S20). In H_2_O_2_‐treated cells simulating diabetic oxidative stress, Musc@CP significantly reduced intracellular ROS levels versus controls (Figure 2n and Figure S21). Collectively, these findings demonstrate that Musc@CP effectively disrupts the oxidative stress cycle.

### 2.3 Transcriptomic Insights Into Musc@CP‐Mediated Perfusion Enhancement

**FIGURE 3:**
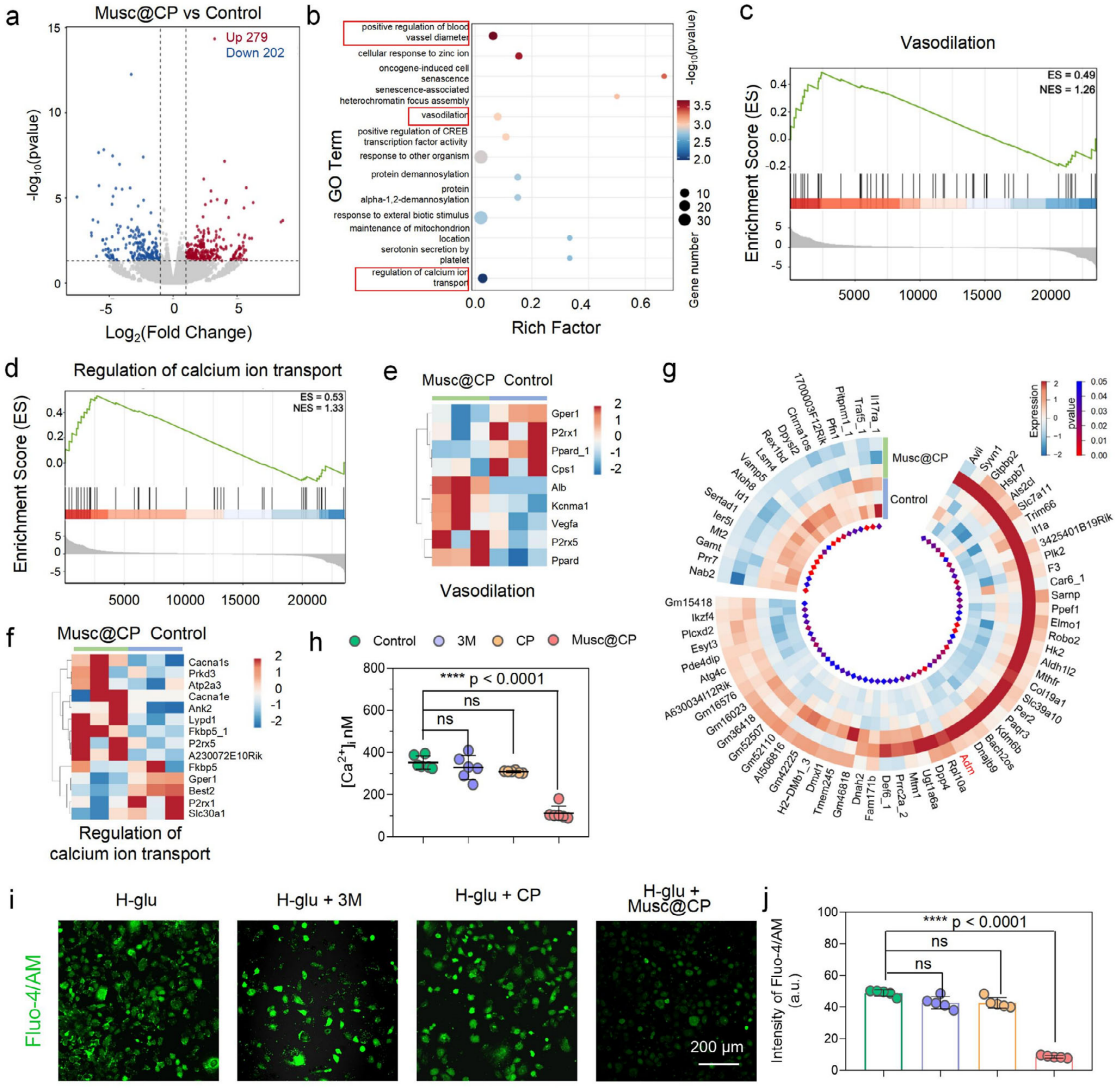
Transcriptomic Insights into Musc@CP‐Mediated Perfusion Enhancement. (a) Volcano plot of DEGs between the Musc@CP group and Control group. (b) GO biological process enrichment analysis of DEGs between the Musc@CP and Control groups. (c,d) GSEA enrichment plots for the GO biological process Terms (Vasodilation, Regulation of calcium ion transport). (e,f) Heatmaps of DEGs involved in GO biological process Terms (Vasodilation, Regulation of calcium ion transport). (g) Circos heatmap of DEGs selected with a lowered threshold (pvalue > 0.05, 0.585 < |log_2_(fold change)| < 1). (h) Quantification of intracellular free Ca^2+^ concentrations using a microplate reader. (i) Representative Fluo‐4/AM staining images and (j) quantitative analysis of the mean fluorescence intensity of Fluo‐4/AM in C166 cells from different treatment groups. (n = 5 independent cells).

To systematically investigate the mechanism by which Musc@CP enhances blood perfusion, we performed RNA‐seq transcriptomic analysis to obtain genome‐wide expression profiles and identify key regulatory pathways (Figure S22). Comparative analysis between Musc@CP and control groups identified 481 differentially expressed genes (DEGs), with 279 upregulated and 202 downregulated genes (Figure 3a and Figures S23 and S24). Gene Ontology (GO) enrichment analyses revealed significant alterations in biological processes related to “Vasodilation” and “Regulation of calcium ion transport” (Figure 3b and Figure S25). GSEA further demonstrated significant upregulation of both vasodilation and calcium ion transport pathways (Figure 3c,d), with key upregulated genes including *Vegfa* and *Cacna1*, respectively (Figure 3e,f). As *Vegfa* serves as an important regulator of angiogenesis, this indicates that Musc@CP can effectively promote neovascularization. Concurrently, the elevated expression of *Cacna1s* and *Cacna1e* suggests that Musc@CP modulates the transcriptional profile of calcium ion transport‐associated pathways, which may contribute to the active remediation of intracellular calcium homeostasis. Combined with GO and GSEA enrichment analyses, these transcriptomic alterations indicate that Musc@CP regulates calcium signaling networks at the genomic level, which may systematically contribute to the mitigation of pathological intracellular Ca^2+^ overload [37]. To further investigate the mechanisms underlying vasodilation, a more lenient threshold for screening differentially expressed genes (p value < 0.05, 0.585 < |log_2_(fold change)| < 1) was applied (Figure 3g). Among the genes identified under this expanded criterion, *Adm* was significantly highlighted, as it encodes a potent vasodilatory peptide whose upregulation is widely recognized as a crucial compensatory and protective response in cardiovascular homeostasis. Furthermore, the protein‐protein interaction (PPI) network reveals tightly clustered functional modules among these differentially expressed candidates (Figure S26), wherein *Ddx39b* and *Atp5a1* are identified as the top hub genes. These hubs collectively reshape the endothelial cellular state by suppressing aberrant inflammatory signaling and augmenting metabolic energy supply, thereby establishing an optimized microenvironment to support robust vasodilation.

In diabetic conditions, hyperglycemia disrupts calcium ion homeostasis, leading to excessive intracellular calcium accumulation and subsequent overload in vascular endothelial cells [38, 39] This pathological cascade triggers profound endothelial dysfunction and ultimately impairs microcirculatory blood flow [40, 41]. Based on this mechanistic understanding, we hypothesized that Musc@CP could rescue vascular function by attenuating intracellular Ca^2+^ overload‐associated endothelial dysfunction. Experimentally, compared to 3M and CP controls, Musc@CP treatment effectively alleviated intracellular Ca^2+^ overload. This was quantified using a fluorescence‐based microplate assay (Figure 3h) and visually confirmed by Fluo‐4/AM staining (Figure 3i,j). Collectively, these findings demonstrate that Musc@CP successfully attenuates aberrant intracellular Ca^2+^ overload and its associated endothelial dysfunction, thereby leading to the observed enhancement in local blood perfusion.

### 2.4 The In Vitro Antibacterial and Hemostatic Properties of Musc@CP

Having established that Musc@CP enhances perfusion by inhibiting endothelial calcium overload, we next evaluated its auxiliary antibacterial and hemostatic functions, which are clinically relevant for managing infection and bleeding complications in diabetic wounds. Bacterial infection impedes diabetic wound healing, as the hyperglycemic environment weakens host defenses and promotes bacterial colonization and biofilm growth [42, 43]. We next assessed the in vitro antibacterial activity of Musc@CP. Against Staphylococcus aureus (*S. aureus*), CP and Musc@CP reduced bacterial survival to 7.8% ± 2.2% and 5.7% ± 1.2%, respectively, which were significantly lower than the 66.0% ± 1.9% observed with the 3M dressing (Extended Data Figure S2a,b). OD_600_ growth curves confirmed this inhibition (Extended Data Figure S2c). Against methicillin resistant *S. aureus* (MRSA), CP and Musc@CP suppressed survival to 11.4% ± 3.1% and 9.7% ± 0.9%, respectively, outperforming the 3M control (37.8% ± 3.4%) (Extended Data Figure S2d,e). OD_600_ kinetics showed sustained suppression, with Musc@CP being the most potent (Extended Data Figure S2f). SEM revealed severe membrane damage, including collapse and fusion, in bacteria treated with CP or Musc@CP, whereas untreated bacteria had smooth, intact surfaces (Extended Data Figure S2g). Live/dead staining further confirmed a significantly lower proportion of viable bacteria in the CP and Musc@CP groups compared to controls (Extended Data Figure S2h–k). The excellent antibacterial effect of Musc@CP can be attributed to the quaternized chitosan, as it contains permanent positively charged quaternary ammonium groups. These stable cationic groups maintain strong electrostatic interactions with the negatively charged bacterial cell membrane, disrupting its integrity and leading to cytoplasmic leakage and cell death [44].

Impaired hemostasis is a critical challenge in diabetic wound care, as chronic hyperglycemia increases blood viscosity and alters platelet function, prolonging clotting and bleeding times [45]. To address this, we evaluated the hemostatic performance of the Musc@CP scaffold. In vitro blood clotting assays showed that both CP and Musc@CP had a significantly lower Blood Clotting Index than the 3M dressing (Extended Data Figure S3a), and SEM revealed extensive red blood cell adhesion and aggregation on the scaffold surfaces (Extended Data Figure S3b). Mechanistically, activated partial thromboplastin time (APTT) was significantly shortened in the CP and Musc@CP groups, while prothrombin time (PT) remained unchanged, indicating activation of the intrinsic coagulation pathway (Extended Data Figure S3c,d) [46]. In vivo, using a diabetic mouse tail transection model, both CP and Musc@CP shortened hemostasis time to approximately 115 s, about 2.7 times faster than the 3M group (Extended Data Figure S3e–g), and significantly reduced blood loss (Extended Data Figure S3h). In a diabetic mouse liver hemorrhage model, Musc@CP achieved a bleeding time of 28.7 ± 3.3 s and blood loss of 13.9 ± 2.8 mg, outperforming both the 3M and Control groups (Extended Data Figure S3i–l). Therefore, beyond its core mechanism of alleviating endothelial Ca^2+^ overload, the Musc@CP scaffold provides robust support for localized fluid management and initial hemostasis, thereby actively contributing to the orchestration of the complex microenvironmental remodeling required for diabetic wound repair.

### 2.5 In Vivo Evaluation of Diabetic Wound Healing

**FIGURE 4:**
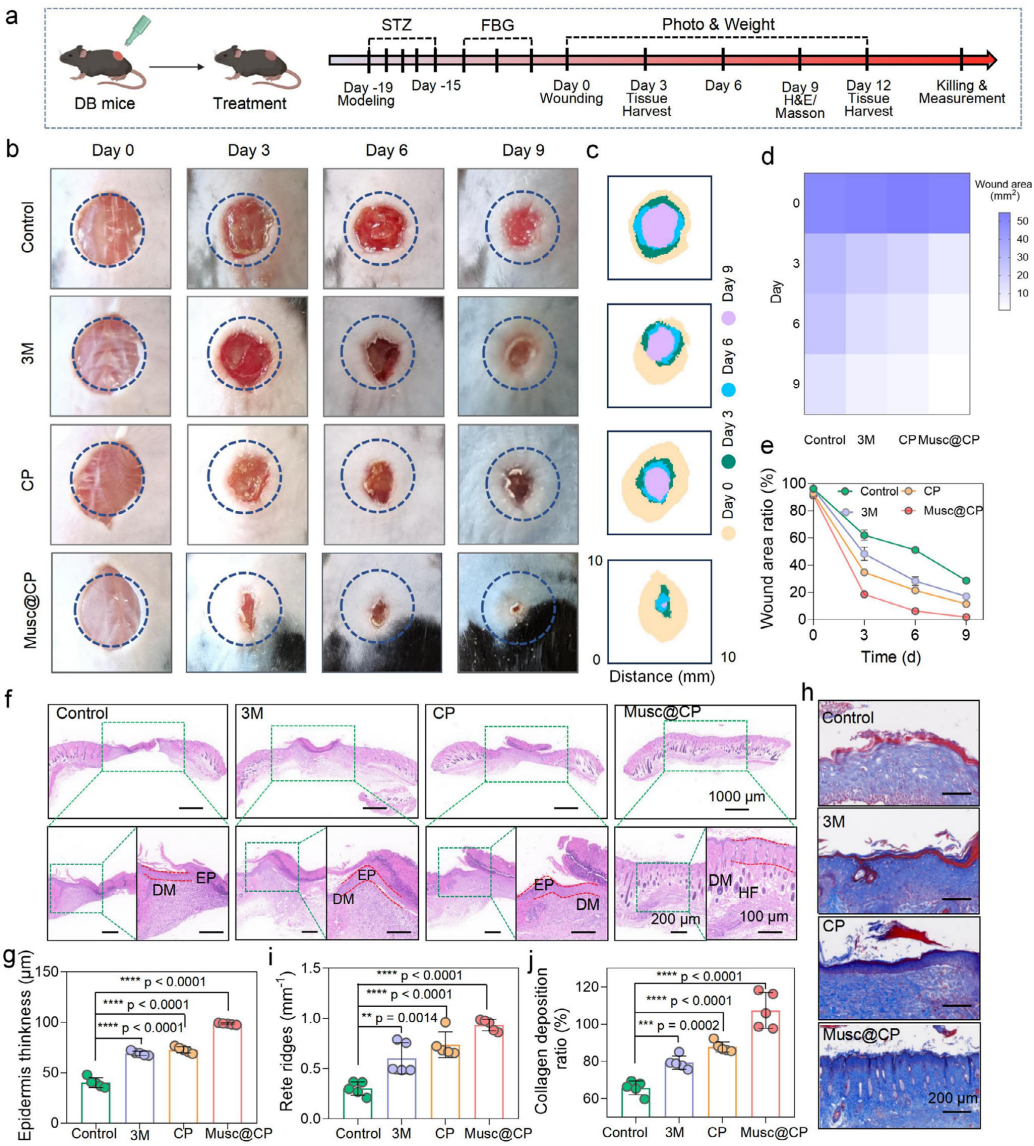
Effects of Musc@CP on promoting wound healing in diabetic C57BL/6 mice with bacteria‐free wounds. (a) Schematic depiction of the sequence of animal experiments conducted to evaluate the therapeutic efficacy of Musc@CP. (b) Photographs of representative wounds from various treatment groups. (c) Quantitative investigation of the wound area. Each experiment was repeated five times independently with similar results. (d) Statistical analysis of the healing time. (e) Statistical analysis of the wound closure ratio. (f) H*&*E‐stained wounds in different treatment groups on day 9. EP, DM and HF stand for epidermis, dermis and hair follicle, respectively. Each experiment was repeated five times independently with similar results. (g) Quantitative analysis of the thickness of the epidermis. (h) Representative images of MST‐stained wounds in different treatment groups on day 9. (i) The area of rete ridges per millimetre length of wounds. (j) Quantitative analysis of collagen deposition in wounds of different treatment groups on day 9. The data are presented as the mean ± s.d. (n = 5 independent mice). Statistical differences were analyzed by one‐way ANONA.

To evaluate the therapeutic efficacy of Musc@CP, we first established a full‐thickness skin wound model in diabetic mice (Figure 4a). Macroscopic imaging and quantitative analysis demonstrated that the Musc@CP group exhibited the most effective healing outcome (Figure 4b–d). The wounds in this group achieved a wound closure rate of 98.1% by day 9, which was significantly higher than that in the CP (88.7%) and 3M (81.1%) groups (Figure 4e). Moreover, the Musc+CP group exhibited a residual wound area of approximately 21%, which was marginally reduced relative to the Control group but remained significantly larger than that of the Musc@CP group (Figure S27). These findings provide direct evidence that simple physical mixing is insufficient to achieve the outstanding therapeutic efficacy conferred by Musc@CP.

Histological examination on day 9 provided further insight into tissue regeneration. Hematoxylin and eosin (H*&*E) staining on day 3 revealed that Musc@CP treatment most rapidly accelerated the resolution of the inflammatory phase (Figure S28). This was evidenced by a significantly faster clearance of inflammatory infiltrates, an effect associated with the enhanced anti‐inflammatory microenvironment. H*&*E staining on day 9 revealed that the Musc@CP group developed the thickest epidermis, indicating enhanced proliferative activity of basal keratinocytes (Figure 4f,g). Furthermore, Masson's trichrome (MST) staining revealed that the Musc@CP group possessed the most prominent rete ridges, along with early‐stage, well‐organized collagen bundle alignment, the highest collagen deposition ratio, and the most apparent regeneration of skin appendages such as hair follicles (Figure 4h‐j and Figure S29). In contrast, the Control, 3M and CP groups displayed incomplete regeneration of the epidermis and dermis, consistent with delayed healing.

The biosafety of Musc@CP was thoroughly evaluated. All mice maintained a stable increase in body weight throughout the experiments (Figure S30). Histopathological examination of major organs (heart, liver, spleen, lungs, and kidneys) collected at the endpoint showed intact tissue architectures with no signs of damage in the Musc@CP group (Figure S31). Analysis of liver and kidney function markers revealed no statistically significant differences between the Musc@CP group and controls (Figure S32), confirming the absence of hepatorenal toxicity and reaffirming the favorable biosafety profile of Musc@CP.

### 2.6 In Vivo Evaluation of Bacterial‐Infected Diabetic Wound Healing

**FIGURE 5:**
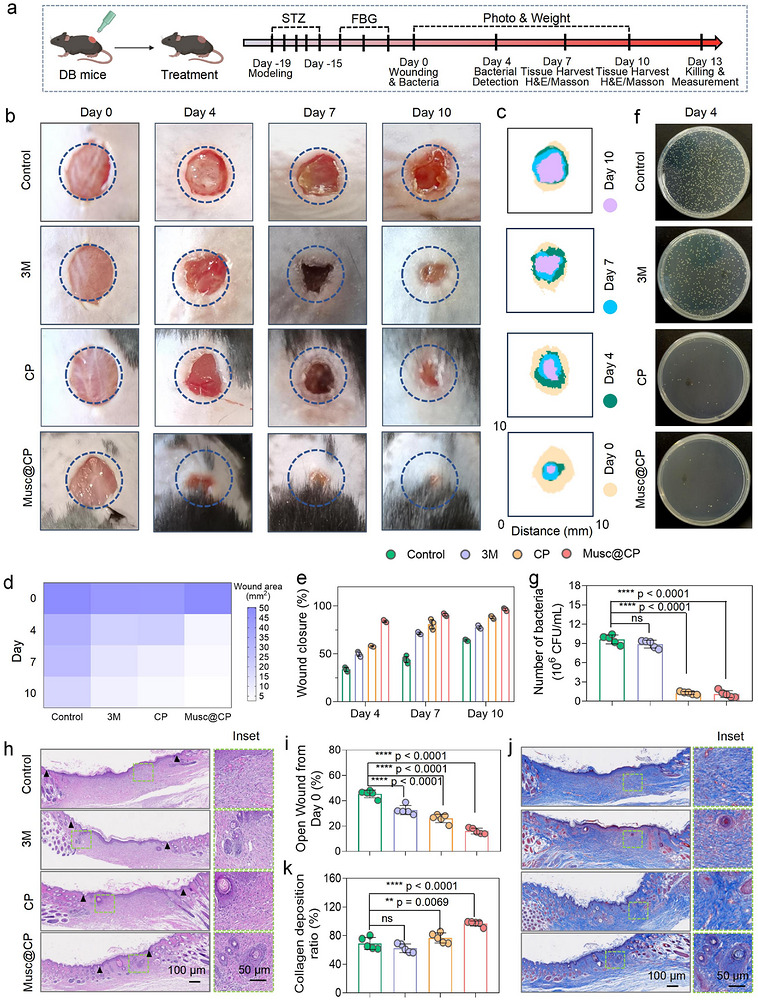
Effects of Musc@CP on enhancing wound healing in infected diabetic mice. (a) Schematic illustration of the sequence of animal experiments carried out to assess the therapeutic efficacy. (b) Photographs of representative wounds. (c) Quantitative investigation of the wound area. (d) Statistical analysis of the healing time. (e) Statistical analysis of the wound area ratio. (f) Representative images for quantifying the residual bacterial bioburden on wound beds under various treatments on day 4. (g) The number of live bacteria. (h) H*&*E‐stained images of the wound tissue on day 13. (i) Quantification of the wound closure is expressed as the percentage of the open wound area compared to that on day 0. Statistical significance and pvalue were determined by one‐way ANOVA followed by Tukey's multiple comparison test. (j) MST‐stained images of the wound tissue on day 13. (k) Quantification of collagen deposition was performed. Statistical significance and pvalue were determined using one‐way ANOVA followed by Tukey's multiple comparison test. (n = 5 mice in each group).

Secondary bacterial infection complicates management and delays healing in approximately 60%–70% of chronic diabetic wounds [47]. To model this challenging clinical scenario, we created full‐thickness skin wounds in diabetic mice and inoculated them with MRSA (Figure 5a). Macroscopic evaluation showed the most rapid wound closure in the Musc@CP group, with wounds nearly fully healed by day 10. The final healing rate reached 96.6% ± 1.3%, significantly surpassing the rates for the CP group (88.3% ± 2.0%) and the 3M group (77.8% ± 2.2%) (Figure 5b–e). Quantification of bacteria from wound tissues confirmed the potent anti‐MRSA activity of Musc@CP (Figure 5f,g), suggesting that the accelerated tissue repair is intimately linked to the effective control of localized infection. Histological analysis corroborated the superior healing quality. On day 13, H*&*E staining showed well‐defined granulation tissue and the smallest residual wound area in the Musc@CP group (Figure 5h,i). The MST staining further demonstrated that Musc@CP treatment fostered the most mature regeneration of skin appendages and the highest degree of organized collagen deposition, signifying effective extracellular matrix remodeling (Figure 5j,k). Throughout the study, the Musc@CP dressing exhibited excellent biocompatibility (Figures S33–S35).

## 3 Conclusion

This study addresses the dual challenge of extracellular matrix disintegration and microcirculatory failure in diabetic wound healing by introducing a synergistic therapeutic strategy that integrates biomimetic structural design with active hemodynamic modulation. The Musc@CP dressing, composed of a chitosan‐pullulan nanofibrous scaffold and the vasoactive agent muscone, recapitulates key structural features of the native ECM to direct fibroblast migration and proliferation. Concurrently, it rescues local perfusion by attenuating intracellular Ca^2+^ overload‐associated endothelial dysfunction. These coordinated effects correlate with a shift in the immune microenvironment toward a pro‐regenerative state. Equipped with auxiliary broad‐spectrum antibacterial and hemostatic properties, Musc@CP significantly accelerates wound closure and enhances tissue regeneration in both sterile and infected diabetic models. Ultimately, our work establishes an integrated platform that simultaneously addresses critical pathological bottlenecks, offering a translatable blueprint for advanced diabetic wound care.

## 4 Experimental Section

### 4.1 Materials

All chemical and biological reagents were commercially sourced. Chitosan (95% quaternized) and Pullulan came from Beyotime. Assay‐specific kits and probes (FAP, Calcein AM/PI, DCFH‐DA) were purchased from Solarbio, while cell culture media and solutions (DMEM, FBS, trypsin‐EDTA, PBS) were obtained from Gibco Life Technologies. Beyotime also supplied the Fluo‐4/AM probe. Preparation protocols and full characterization data for the Musc@CP scaffold can be found in Text S1. A complete list of antibodies is provided in Text S2. Experimental procedures for assessing contact angle, surface morphology, and fluid management properties are documented in Text S3.

### 4.2 Cell Culture

Mouse fibroblasts cells (L929), Mouse Embryonic Fibroblast Cells (NIH/3T3), Mouse vascular endothelial cells (C166) were purchased from BeNa Culture Collection (BNCC). Dulbecco's modified Eagle's medium (DMEM; Gibco) supplemented with 10% FBS, was employed to incubate cell lines L929, NIH/3T3 and C166.

The detailed experimental procedures for cytotoxicity and cell migration are described in Text S4.

### 4.3 In Vitro Clotting Test

The blood clotting assay was conducted with citrate‐anticoagulated whole blood. Solution preparation involved sodium citrate (38 mg mL^−1^, Solution A) and calcium chloride (0.2 M, Solution B). A pre‐cut dressing (15 mm × 15 mm) was equilibrated in a 15 mL tube at 37°C for 30 min. Subsequently, 1 mL of citrated blood (a 1:1 mix of whole blood and Solution A) was added to cover the sample. The coagulation process was started by introducing 75 µL of Solution B. After 5 and 10 min, 10 mL of deionized water was slowly added to the tube. Following dressing removal, the liquid was centrifuged. The resulting supernatant was combined with 20 mL of deionized water and held at 37°C for 1 h. Absorbance measurement was performed by transferring 200 µL of the final liquid to a 96‐well plate and reading the absorbance at 540 nm (recorded as a). The absorbance of 1 mL of whole blood and solution A mixed with 75 mL of deionized water was recorded as b. The value of a/b was calculated to obtain the coagulation index BCI (%) (the average of three repeated experiments, n = 3; using the same batch of blood). Since hemoglobin comes from uncoagulated red blood cells, a lower BCI indicates a faster coagulation rate.

In addition, the adhesion of blood cells to different dressings was further investigated. The wound dressings were cut into 1 cm × 1 cm squares and placed in 24‐well plates. Blood was taken from mice and mixed evenly with the anticoagulant in the anticoagulant tube, then dropped into the well plates and incubated at 37°C for 30 min. Subsequently, the red blood cells were fixed with 2.5% paraformaldehyde solution for 2 h. After the fixation was completed, the wound dressings loaded with red blood cells were gradually dehydrated with 25%, 50%, 75%, 95%, and 100% ethanol for 15 min at each concentration. Finally, they were freeze‐dried and the adhesion of red blood cells was observed under a scanning electron microscope.

### 4.4 In Vivo Clotting Test

#### 4.4.1 Skin Hemostasis

The experimental mice were randomly assigned to four groups. Subsequently, the mice were anesthetized using 0.25% tribromoethanol (Avertin) and secured on the operating board. The tails of the mice were wiped with alcohol, and then cut 1.5 cm from the root. The amount and time of bleeding were measured. The filter paper was weighed in advance and placed under the tail of the mouse to collect samples.

#### 4.4.2 Liver Hemostasis

The mice were anesthetized by intraperitoneal injection of Avertin. The hair in the abdominal surgical area was removed with a depilatory knife, and the area was disinfected with iodophor. The abdominal skin was cut open at the xiphoid process with tissue scissors, and the epidermis was held with tissue forceps. The muscle and fascia were cut layer by layer to open the abdominal cavity. The excess blood was wiped off with gauze, and the liver was removed from the abdominal cavity and fully exposed. Gauze and quantitative filter paper were placed under the exposed liver. The liver was cut with a scalpel, and 3M, CP and Musc@CP dressing was applied. The bleeding situation at the incision was observed, and the bleeding time and volume were measured.

### 4.5 Attenuation of Intracellular Ca^2+^ Overload by Musc@CP

The effect of Musc@CP on [Ca^2+^]_i_ was evaluated using a multi‐functional microplate reader. To further verify the capability of Musc@CP in attenuating intracellular Ca^2+^ overload, the fluorescence intensity of Fluo‐4 AM was observed by confocal microscopy to monitor intracellular calcium levels. Fluo‐4 AM is a fluorescent dye that can penetrate the cell membrane and is an acetylmethyl ester derivative of Fluo‐4. Due to the extremely weak fluorescence of Fluo‐4 AM, it does not increase with the increase of Ca^2+^ concentration. Once inside the cell, Fluo‐4 AM is hydrolyzed by intracellular esterases to yield Fluo‐4, which emits strong fluorescence upon binding Ca^2+^. This fluorescence, with excitation/emission maxima at 494 and 516 nm, is typically detected using the FITC channel (excitation ∼488 nm).

### 4.6 RNA‐seq Analysis

Total RNA was isolated from C166 cells using TRIzol reagent (n = 3). RNA integrity and concentration was measured with NanoDrop ND‐2000 and Agilent 2100/4200 Bioanalyzer. After amplification into a cDNA library by PCR, sequencing was performed on the Illumina NovaSeq 6000 platform in PE150 mode. Differentially expressed gene analysis was performed in R language through the DESeq2 package (version 1.46.0). To interpret the biological functions of the DEGs, GO Enrichment Analysis and KEGG pathway enrichment analysis were performed in R language through the clusterProfiler package (version 4.14.6). Furthermore, GSEA was conducted by ranking all genes based on their log_2_(fold change) values with clusterProfiler package. According to the official nomenclature guidelines of the Mouse Genome Informatics (MGI) database, mouse gene symbols are presented with only the first letter capitalized.

### 4.7 Flow Cytometry Analysis and Detection of Macrophage Cytokines

The details are described in Text S5.

### 4.8 ROS Scavenging Capacity

The ROS scavenging capacity of Musc@CP was evaluated using H_2_O_2_ and •O_2_^−^ as representative ROS. The details are described in Text S6.

### 4.9 Antibacterial Performance

Preparation of bacterial SEM samples. In order to observe the morphology of bacteria, we co‐cultured the bacteria with Musc@CP material. Subsequently, the material was removed, and the bacterial cells were collected via centrifugation and rinsed with PBS. Then, the cells were subjected to serial dilution. The bacteria were fixed using a 2.5% paraformaldehyde solution at 4°C overnight and then dehydrated through a series of ethanol gradients, namely 25%, 50%, 75%, 95%, and 100%. The treated bacterial suspension was dropped onto a silicon wafer, left to air‐dry naturally, and then fixed onto a sample stage using conductive adhesive. A 60 s platinum sputtering coating was applied for scanning electron microscopy observation.

Plate colony count method. The bacterial activity was quantified by means of the plate spread method. The treated bacteria were collected, rinsed once with PBS, and resuspended. The bacterial suspension was adjusted to an OD_600_ of 0.1. One milliliter of this suspension was used to soak each group of nanofiber dressings and incubated at room temperature in the dark for 24 h. After incubation, the dressings were removed, and the bacterial – containing liquid was entirely transferred to a centrifuge tube and rinsed twice with 1 mL of PBS. The combined eluate was approximately 2 mL. This eluate was diluted to an appropriate concentration (about 10^4^ CFU mL^−1^), and an appropriate amount was spread onto sterile LB agar plates, which were then incubated overnight at 37°C. The next day, the growth of colonies was observed and photographed, and the actual number of viable bacteria was calculated by counting the colonies on each plate.

The specific steps of the bacterial viability staining experiment are detailed in Text S7.

### 4.10 Animal Study

Male C57BL/6J mice (6 weeks, 14–16 g) provided by Beijing HFK Bioscience Company were used in this study. The details are described in Text S8.

### 4.11 Establishment of an Infected Diabetic Wound Model

To evaluate the therapeutic efficacy of Musc@CP, a mouse model of infected diabetic wounds was created. The details are described in Text S9.

### 4.12 Infected Diabetic Wound Healing

After the establishment of the infectious diabetic wound model, under the anesthesia of mice, 3M, CP or Musc@CP nanofibers were applied to cover the entire wound area and fixed with hollowed‐out medical transparent adhesive tape. The details are described in Text S10.

## Author Contributions

**Xiang Zheng**: conceptualization, methodology, data curation, validation, Writing – original draft, investigation, formal analysis, software. **Mingmei Li**: conceptualization, methodology, investigation, funding acquisition, writing – review and editing. **Jianjun Jiang**: data curation, software. **Yongchao Wang**: data curation, conceptualization. **Yaru Jia**: data curation, visualization. **Weilun Sun**: validation. **Yi Yao**: data curation. **Pengli Gao**: funding acquisition. **Linhua Zhang**: formal analysis. **Qi Guo**: data curation. **Guang Jia**: data curation. **Xing‐Jie Liang**: methodology, conceptualization, resources, writing – review and editing. **Dunwan Zhu**: methodology, writing – review and editing. **Jinchao Zhang**: writing – review and editing, funding acquisition, project administration. **Fangzhou Li**: conceptualization, funding acquisition, writing – review and editing, project administration, supervision.

## Conflicts of Interest

The authors declare no conflicts of interest.

## Supporting information




**Supporting File**: advs76758‐sup‐0001‐SuppMat.docx.

## Data Availability

The data that support the findings of this study are available from the corresponding author upon reasonable request.

## References

[1] Y. Shen , S. Li , X. Hou , et al., “Ultrasound‐Triggered Nanocomposite “Lever” Hydrogels With a Full Repair System Accelerates Diabetic Foot Ulcer Repair,” Advanced Science 12, no. 23 (2025): 2500720, 10.1002/advs.202500720. 40344623 PMC12199430

[2] Z. Hu , S. Qian , B. Liao , et al., “Apoptotic Vesicle Membrane‐Mediated Targeted Endothelial Mitochondrial Transplantation‐Clearance Therapy for Diabetic Wound Healing,” Research 9 (2026): 1042, 10.34133/research.1042. 42058651 PMC13123279

[3] J. Liu , Q. Song , W. Yin , et al., “Bioactive Scaffolds for Tissue Engineering: A Review of Decellularized Extracellular Matrix Applications and Innovations,” Exploration 5 (2025): 20230078, 10.1002/EXP.20230078. 40040827 PMC11875452

[4] G. S. Hussey , J. L. Dziki , and S. F. Badylak , “Extracellular Matrix‐Based Materials for Regenerative Medicine,” Nature Reviews Materials 3, no. 7 (2018): 159–173, 10.1038/s41578-018-0023-x.

[5] B. Li , W. Yang , R. Shu , et al., “Antibacterial and Angiogenic (2A) Bio‐Heterojunctions Facilitate Infectious Ischemic Wound Regeneration via an Endogenous–Exogenous Bistimulatory Strategy,” Advanced Materials 36, no. 6 (2024): 2307613, 10.1002/adma.202307613. 37848208

[6] O. A. Peña and P. Martin , “Cellular and Molecular Mechanisms of Skin Wound Healing,” Nature Reviews Molecular Cell Biology 25 (2024): 599–616, 10.1038/s41580-024-00715-1. 38528155

[7] Y. Zhao , L. Luo , L. Huang , et al., “ *In Situ* Hydrogel Capturing Nitric Oxide Microbubbles Accelerates the Healing of Diabetic Foot,” Journal of Controlled Release 350 (2022): 93–106, 10.1016/j.jconrel.2022.08.018. 35973472

[8] B. Zhou , Y. Duan , W. Li , et al., “A Tailored Hydrogel with Local Glycemia Management, Antioxidant Activity, and Photothermal Antibacterial Properties for Diabetic Wound Healing,” Advanced Science 12, no. 16 (2025): 2414161, 10.1002/advs.202414161. 40041979 PMC12021113

[9] M. Lu , Z. Luo , H. Chen , and Y. Zhao , “Hierarchical Micro/Nano‐Structured Biomaterials for Wound Healing,” Research 8 (2025): 0968, 10.34133/research.0968. 41255571 PMC12620629

[10] L. Wei , X. Liu , Y. Li , et al., “Multifunctional PHC Bandage for Accelerated Wound Healing in Movable Parts,” Exploration 5 (2025): 20230176, 10.1002/EXP.20230176. 40585762 PMC12199424

[11] T. Li , C. Chen , A. H. Brozena , et al., “Developing Fibrillated Cellulose as a Sustainable Technological Material,” Nature 590, no. 7844 (2021): 47–56, 10.1038/s41586-020-03167-7. 33536649

[12] Y. Jiao , X. Li , J. Chen , et al., “Constructing Nanoscale Topology on the Surface of Microfibers Inhibits Fibroblast Fibrosis,” Advanced Fiber Materials 4, no. 5 (2022): 1219–1232, 10.1007/s42765-022-00165-4.

[13] C. T. Laurencin , A. M. A. Ambrosio , M. D. Borden , and J. A. Cooper , “Tissue Engineering: Orthopedic Applications,” Annual Review of Biomedical Engineering 1, no. 1 (1999): 19–46, https://www.researchgate.net/publication/11652180. 10.1146/annurev.bioeng.1.1.19 11701481

[14] M. C. Benn , S. A. Pot , J. Moeller , et al., “How the Mechanobiology Orchestrates the Iterative and Reciprocal ECM‐Cell Cross‐Talk That Drives Microtissue Growth,” Science Advances 9, no. 13 (2023): add9275, https://pubmed.ncbi.nlm.nih.gov/36989370/. 10.1126/sciadv.add9275 PMC10058249 36989370

[15] Z. Li , L. Li , M. Yue , Q. Peng , X. Pu , and Y. Zhou , “Tracing Immunological Interaction in Trimethylamine N‐Oxide Hydrogel‐Derived Zwitterionic Microenvironment During Promoted Diabetic Wound Regeneration,” Advanced Materials 36, no. 33 (2024): 2402738, 10.1002/adma.202402738. 38885961

[16] Y. Zhou , S. Guo , B. O. A. Botchway , Y. Zhang , T. Jin , and X. Liu , “Muscone Can Improve Spinal Cord Injury by Activating the Angiogenin/Plexin‐B2 Axis,” Molecular Neurobiology 59 (2022): 5891–5901, 10.1007/s12035-022-02948-7. 35809154

[17] X. Gu , N. Bao , J. Zhang , et al., “Muscone Ameliorates Myocardial Ischemia‐Reperfusion Injury by Promoting Myocardial Glycolysis,” Heliyon 9 (2023): e22154, 10.1016/j.heliyon.2023.e22154. 38045159 PMC10692826

[18] K. Bera , A. Kiepas , I. Godet , et al., “Extracellular Fluid Viscosity Enhances Cell Migration and Cancer Dissemination,” Nature 611, no. 7935 (2022): 365–373, 10.1038/s41586-022-05394-6. 36323783 PMC9646524

[19] X. Zhao , P. Psarianos , L. S. Ghoraie , et al., “Metabolic Regulation of Dermal Fibroblasts Contributes to Skin Extracellular Matrix Homeostasis and Fibrosis,” Nature Metabolism 1, no. 1 (2019): 147–157, 10.1038/s42255-018-0008-5. 32694814

[20] X. Wang , M. Shen , M. Ma , et al., “Rational Construct of Extracellular Matrix Mimics via Peptide‐Co‐Assembling Nanofibers for Efficient Bone Regeneration,” Advanced Fiber Materials 7, no. 4 (2025): 1093–1110, 10.1007/s42765-025-00536-7.

[21] Y. Bai , X. Zheng , X. Zhong , et al., “Manipulation of Heterogeneous Surface Electric Potential Promotes Osteogenesis by Strengthening RGD Peptide Binding and Cellular Mechanosensing,” Advanced Materials 35, no. 24 (2023): 2209769, 10.1002/adma.202209769. 36934418

[22] A. Michna , D. Lupa , W. Plazinski , P. Batys , and Z. Adamczyk , “Physicochemical Characteristics of Chitosan Molecules: Modeling and Experiments,” Advances in Colloid and Interface Science 337 (2025): 103383, 10.1016/j.cis.2024.103383. 39733532

[23] Z. Qin , X. Jia , Q. Liu , B. Kong , and H. Wang , “Enhancing Physical Properties of Chitosan/Pullulan Electrospinning Nanofibers via Green Crosslinking Strategies,” Carbohydrate Polymers 247 (2020): 116734, 10.1016/j.carbpol.2020.116734. 32829855

[24] M. O'Mara , S. Zhang , and U. G. Knaus , “Spatiotemporal H_2_O_2_ Flashes Coordinate Actin Cytoskeletal Remodeling and Regulate Cell Migration and Wound Healing,” Nature Communications 16 (2025): 6868, 10.1038/s41467-025-62272-1. PMC12297405 40715145

[25] Y.‐C. Li , Y. Li , Y.‐N. Zhang , et al., “Muscone and (+)‐Borneol Cooperatively Strengthen CREB Induction of Claudin 5 in IL‐1*β*‐Induced Endothelium Injury,” Antioxidants 11, no. 8 (2022): 1455, 10.3390/antiox11081455. 35892657 PMC9394259

[26] C. M. Desmet , V. Préat , and B. Gallez , “Nanomedicines and Gene Therapy for the Delivery of Growth Factors to Improve Perfusion and Oxygenation in Wound Healing,” Advanced Drug Delivery Reviews 129 (2018): 262–284, 10.1016/j.addr.2018.02.001Get. 29448035

[27] S. S. Verma , C. K. Sen , R. Srivastava , et al., “Tissue Nanotransfection‐Based Endothelial PLCγ2‐Targeted Epigenetic Gene Editing Rescues Perfusion and Diabetic Ischemic Wound Healing,” Molecular Therapy 33, no. 3 (2025): 950–969, 10.1016/j.ymthe.2025.01.034. 39863930 PMC11897775

[28] H. J. Chu , C.‐W. Lee , S.‐C. Tang , and J.‐S. Jeng , “TCTAP A‐057 Endovascular Thrombectomy for Acute Ischemic Stroke: A Single‐Center Experience in Taiwan,” Journal of the American College of Cardiology 67, no. 16 (2016): S24–S25, 10.1016/j.jacc.2016.03.075.

[29] E. Lu , X. Yang , T. Wang , et al., “Biomimetic Thermo‐Sensitive Hydrogel Encapsulating Hemangiomas Stem Cell Derived Extracellular Vesicles Promotes Microcirculation Reconstruction in Diabetic Wounds,” Advanced Functional Materials 33, no. 45 (2023): 2304250, 10.1002/adfm.202304250.

[30] C. A. Fromen , W. J. Kelley , M. B. Fish , et al., “Neutrophil–Particle Interactions in Blood Circulation Drive Particle Clearance and Alter Neutrophil Responses in Acute Inflammation,” ACS Nano 11, no. 11 (2017): 10797–10807, https://pubmed.ncbi.nlm.nih.gov/29028303/. 29028303 10.1021/acsnano.7b03190 PMC5709153

[31] H. Liu , S. Qin , H. Zhang , et al., “Silk Sericin‐based ROS‐Responsive Oxygen Generating Microneedle Platform Promotes Angiogenesis and Decreases Inflammation for Scarless Diabetic Wound Healing,” Advanced Functional Materials 35, no. 7 (2025): 2404461, 10.1002/adfm.202404461.

[32] X. Gao , S. Li , F. Ding , et al., “A Virus‐Mimicking Nucleic Acid Nanogel Reprograms Microglia and Macrophages for Glioblastoma Therapy,” Advanced Materials 33, no. 9 (2021): 2006116, 10.1002/adma.202006116. 33501743

[33] N. Zhang , X. Wang , M. Feng , et al., “Early‐Life Exercise Induces Immunometabolic Epigenetic Modification Enhancing Anti‐Inflammatory Immunity in Middle‐Aged Male Mice,” Nature Communications 15, no. 1 (2024): 3103, 10.1038/s41467-024-47458-3. PMC11006929 38600123

[34] Z. Xu , W. Yang , R. Zhang , et al., “Microenvironment‐Programmed siRNA‐Based Hydrogel for Spatiotemporal Gene Silencing in Wound Healing,” Advanced Materials 37, no. 47 (2025): 09558, 10.1002/adma.202509558. 40913576

[35] R. Fan , J. Zhao , L. Yi , et al., “Anti‐Inflammatory Peptide‐Conjugated Silk Fibroin/Cryogel Hybrid Dual Fiber Scaffold with Hierarchical Structure Promotes Healing of Chronic Wounds,” Advanced Materials 36, no. 16 (2024): 2307328, 10.1002/adma.202307328. 38288789

[36] X. Wu , H. Zhu , C. Song , Q. Tan , Y. Zhao , and L. Shang , “Breadmaking‐Inspired Antioxidant Porous Yeast Microcarriers for Stem Cell Delivery in Diabetic Wound Treatment,” Advanced Materials 36, no. 2 (2024): 2309719, 10.1002/adma.202309719. 37985138

[37] N. Korte , A. Barkaway , J. Wells , et al., “Inhibiting Ca^2+^ Channels in Alzheimer's Disease Model Mice Relaxes Pericytes, Improves Cerebral Blood Flow and Reduces Immune Cell Stalling and Hypoxia,” Nature Neuroscience 27, no. 11 (2024): 2086–2100, 10.1038/s41593-024-01753-w. 39294491 PMC11537984

[38] K. D. Nadezhdin , I. A. Talyzina , A. Parthasarathy , A. Neuberger , D. X. Zhang , and A. I. Sobolevsky , “Structure of Human TRPV4 in Complex with GTPase RhoA,” Nature Communications 14, no. 1 (2023): 3733, 10.1038/s41467-023-39346-z. PMC10290124 37353478

[39] M. S. Abdul Halim , J. M. Dyson , M. M. Gong , M. K. O'Bryan , and R. Nosrati , “Fallopian Tube Rheology Regulates Epithelial Cell Differentiation and Function to Enhance Cilia Formation and Coordination,” Nature Communications 15, no. 1 (2024): 7411, https://pubmed.ncbi.nlm.nih.gov/39198453/. 10.1038/s41467-024-51481-9 PMC11358425 39198453

[40] W. Wong , J. Stajic , G. Chin , et al., “This Week in Science,” Science 357, no. 6346 (2017): 43–45, https://pubmed.ncbi.nlm.nih.gov/28684509/.

[41] A. Balistrieri , A. Makino , and J. X.‐J. Yuan , “Pathophysiology and Pathogenic Mechanisms of Pulmonary Hypertension: Role of Membrane Receptors, Ion Channels, and Ca^2+^ Signaling,” Physiological Reviews 103, no. 3 (2023): 1827–1897, https://pubmed.ncbi.nlm.nih.gov/36422993/. 36422993 10.1152/physrev.00030.2021 PMC10110735

[42] L. Lu , J. Liao , C. Xu , et al., “Kinsenoside‐Loaded Microneedle Accelerates Diabetic Wound Healing by Reprogramming Macrophage Metabolism via Inhibiting IRE1α/XBP1 Signaling Axis,” Advanced Science 12, no. 26 (2025): 2502293, 10.1002/advs.202502293. 40279546 PMC12245111

[43] F. Wang , H. Lei , C. Tian , et al., “An Efficient Biosynthetic System for Developing Functional Silk Fibroin‐Based Biomaterials,” Advanced Materials 37, no. 7 (2025): 2414878, 10.1002/adma.202414878. 39663673

[44] Z. Liu , K. Guo , L. Yan , et al., “Janus Nanoparticles Targeting Extracellular Polymeric Substance Achieve Flexible Elimination of Drug‐Resistant Biofilms,” Nature Communications 14, no. 1 (2023): 5132, 10.1038/s41467-023-40830-9. PMC10447547 37612285

[45] J. Wang , Y. Yang , H. Xu , S. Huang , B. Guo , and J. Hu , “All‐in‐One: A Multifunctional Composite Biomimetic Cryogel for Coagulation Disorder Hemostasis and Infected Diabetic Wound Healing,” Nano‐Micro Letters 17, no. 1 (2025): 171, 10.1007/s40820-024-01603-1. 40025402 PMC11872855

[46] Y. Guo , M. Wang , Q. Liu , G. Liu , S. Wang , and J. Li , “Recent Advances in the Medical Applications of Hemostatic Materials,” Theranostics 13, no. 1 (2023): 161–196, 10.7150/thno.79639. 36593953 PMC9800728

[47] J. Kong , S. Ma , R. Chu , et al., “Photothermal and Photocatalytic Glycol Chitosan and Polydopamine‐Grafted Oxygen Vacancy Bismuth Oxyiodide (BiO_1‐x_ I) Nanoparticles for the Diagnosis and Targeted Therapy of Diabetic Wounds,” Advanced Materials 36, no. 11 (2024): 2307695, 10.1002/adma.202307695. 38150667

