# Supplementary material for: Modulating Calcium Homeostasis via a Biomimetic Scaffold to Rescue Diabetic Ischemic Wounds
Source: Adv Sci (Weinh). 2026 Jul 27:e76758. Online ahead of print. doi: 10.1002/advs.76758 (PMC13403889; doi:10.1002/advs.76758)

Supplementary Information

**Mitigating Endothelial Calcium Overload-Associated Dysfunction via a Nanoscale Topographical Scaffold to Rescue Diabetic Ischemic Wounds**

*Xiang Zheng^1,2†^, Mingmei Li^2†^*, Jianjun Jiang^2†^, Yongchao Wang^5^, Yaru Jia^1,3^, Weilun Sun^3^, Yi Yao^6^, Pengli Gao^2^, Linhua Zhang^2^, Qi Guo^1^, Guang Jia^1^, Xing-Jie Liang^3,4^, Dunwan Zhu^2^*, Jinchao Zhang^1^*, Fangzhou Li^2^**

†These authors contributed equally to this work.

Dr. X. Zheng, Y. Jia, Q. Guo, G. Jia, Prof. J. Zhang

^1^ College of Chemistry *&* Materials Science, Key Laboratory of Medicinal Chemistry and Molecular Diagnosis of Ministry of Education, State Key Laboratory of New Pharmaceutical Preparations and Excipients, Chemical Biology Key Laboratory of Hebei Province, Hebei University, Baoding 071002, China;

Dr. M. Li, J. Jiang, Dr. P. Gao, Prof. L. Zhang, Prof. D. Zhu, Prof. F. Li

^2^ State Key Laboratory of Advanced Medical Materials and Devices, Tianjin Key Laboratory of Biomedical Materials, Key Laboratory of Biomaterials and Nanotechnology for Cancer Immunotherapy, Institute of Biomedical Engineering, Tianjin Institutes of Health Science, Chinese Academy of Medical Sciences & Peking Union Medical College, Tianjin 300192, China;

W. Sun, Prof. X-J. Liang

^3^ CAS Key laboratory for Biomedical effects of nanomaterials and nanosafety, CAScenter for excellence in nanoscience, National Center for Nanoscience and Technology of China, No. 11, First North Road, Zhongguancun, Beijing, 100190, P. R. China;

^4^ University of Chinese Academy of Sciences, Beijing, 100049, P. R. China.

Prof. Y. Wang

^5^ School of Life Sciences, Zhengzhou University, Zhengzhou 450001, China

Y. Yao

^6^ State Key Laboratory of Natural Medicines, Department of Pharmaceutics, School of Pharmacy, China Pharmaceutical University, Nanjing 211198, P. R. China

Corresponding author: Mingmei Li (mingmeili2023@163.com); Dunwan Zhu (zhudunwan@bme.pumc.edu.cn); Jinchao Zhang (zjc@hbu.edu.cn); Fangzhou Li (fzli@bme.pumc.edu.cn)

**Text S1. Preparation and characterization of Musc@CP**

The mixed solution of Chitosan (with a quaternization degree of 95%) and Pullulan was injected into the high-pressure electrospinning instrument to prepare nanofibers. The push speed was set at 6.7-26.7 μL min^-1^, the voltage at 18-25 kV, and the receiving distance at 6-16 cm. A roller receiver was used to receive the electrospun nanofibers, and the roller speed was set at 200 r min^-1^. Then, the nanofibers were partially immersed in 0, 0.1, and 1 M NaBH4 solutions for 30 minutes respectively, with the foaming solution kept 1 mm below the upper surface to ensure that the upper surface’s ECM-like structure was not damaged. The foamed nanofibers were then transferred to a new plate and washed three times with distilled water. Subsequently, they were placed in a circulating water pump and vacuum pump to remove bubbles for 3 seconds. After being taken out, they were frozen in a -80°C refrigerator for 1 hour and then freeze-dried in a freeze dryer for 24 hours to obtain 3D porous nanofiber CP. Finally, Musc was loaded onto the surface of CP by physical adsorption. Musc was dissolved in anhydrous ethanol, and CP was immersed in 1 mL of Musc solution. After the anhydrous ethanol was completely evaporated, uniform Musc-loaded Musc@CP was prepared.

**Text S2. Antibodies**

APC anti-mouse CD206 (MMR) Antibody, Biolegend (Product # 141707), Dilution 1:200; PE anti-mouse CD86 Antibody, Biolegend (Product # 159204), Dilution 1:200; Mouse momoclonal AntiCD31 antibody, Abcam (Product # ab222783), Dilution 1:500; Mouse momoclonal Anti-alpha smooth muscle Actin (*α*-SMA) antibody, Abcam(Product # ab7817), Dilution 1:500; Goat Anti-Rabbit IgG (H + L) Highly Cross-Adsorbed Secondary Antibody, ThermoFisher (Catalog # A-11034), Dilution 1:1000.

**Text S3. Cell toxicity and migration**

L929 were utilized to assess the cytotoxicity of Musc@CP via an CCK-8 assay. The cells were seeded into the lower chamber of a 24-well transwell plate (each well containing 1 × 10^5^ cells) for 12 h. Subsequently, the supernate of Musc@CP was added to each upper chamber and incubated for another 12 h. Then, CCK-8 solution was added, and the cells were incubated for 2 h. The absorbance at 490 nm was measured via a standardized process. Additionally, calcein-AM (4 × 10^-6^ M) and PI (4 × 10^-6^ M) staining were used to distinguish the distribution of living and dead cells. Cell apoptosis was analyzed via confocal laser scanning microscopy. For the cell migration experiment, 1 × 10^5^ L929 cells were seeded in 24-well plates and allowed to grow for 24 h before being scratched. A 100 μL pipette was subsequently used to draw a uniformly wide line in the center of each well of the 24-well plate to remove cells from that area. The cells were then placed in different environments and divided into four groups: the control group, the 3M group, the CP group, and the Musc@CP group. Cell migration was recorded at 24 h thereafter.

**Text S4. Contact angle, surface morphology and fluid management**

The contact angle (CA) was measured by the sessile drop method. The test was conducted at a room temperature of 25°C and a relative humidity of 60%. The samples were evenly spread and fixed on the glass slides with double-sided tape. Three random positions on each sample were selected for measurement. Each time, 16 μL of distilled water was used, and the contact angle between the water droplet and the sample surface (3M, CP, Musc@CP) was collected at the moment of initial contact during the 55 ms of droplet fall.

Swelling ratio reflects the water absorption performance of wound dressings. The swelling ratio is determined by the mass method. A 500-mesh sieve (8 cm × 8 cm square, folded into a 4 cm × 4 cm × 2 cm square trough) is placed in a drying oven and dried at 80°C to a constant weight, which is recorded as m_0_. The sample is cut into 1 cm × 1 cm pieces and placed on the 500-mesh sieve. The sieve is then placed in an evaporating dish, and 30 mL of distilled water is added at once to fully wet the sample. After the sample has fully swollen (at least 30 minutes), the sieve and sample are removed together. The liquid at the bottom and around the sieve is absorbed with filter paper until there is no wet mark on the filter paper. The weight is recorded as m_1_. Then the sieve is placed in the drying oven and dried at 80°C to a constant weight, which is recorded as m_2_. The swelling ratio is calculated according to the Equation S1.

Swelling ratio（%）= $\frac{m_{1}-m_{2}}{m_{2}-m_{0}}$ × 100% S1

Liquid absorption and liquid retention capacity. (1) Cut the wound dressings into pieces (1 cm × 1 cm), and immerse them in containers filled with four solutions (PBS, high-glucose PBS, blood, high-glucose blood) respectively. After culturing for 30 minutes, take out the dressings, transfer them to empty trays and let them drain naturally for 30 seconds, then weigh them to calculate the water absorption of the wound dressings. (2) Apply a pressure equivalent to 40 mmHg on the swollen area of the wound dressings after absorption for 30 seconds, then reweigh them to calculate the liquid retention capacity of the wound dressings. Perform each test on each type of wound dressing five times and take the average value. (3) Weigh the wound dressings as W_1_, immerse them in PBS/blood for 5 minutes, remove the excess liquid on the surface and weigh them again as W_2_. The amount of PBS/blood absorbed is calculated according to formula: Absorption rate (%) = (W_2_ - W_1_) / W_1_ × 100% Perform each test on each type of wound dressing three times and take the average value.

**Text S5. Flow cytometry analysis and detection of macrophage cytokines**

RAW 264.7 cells were seeded in 6-well plates. After overnight incubation, LPS solution (final concentration 40 ng/mL) was added, and the cells were further cultured for 24 h. The medium was then replaced with fresh medium, in which the experimental groups received medium containing 40 μg/mL CP exudate and 40 μg/mL musc (Musc+CP), or 40 μg/mL Musc@CP exudate, respectively. After 24 h, the cells were collected and labeled with anti‑CD86/PE and anti‑CD206/APC antibodies. Subsequently, the expression of CD86 and CD206 on RAW 264.7 cells was detected by flow cytometry. Stained cells were analyzed using a Cyto‑FLEX flow cytometer (Beckman Coulter Biotechnology) and data were processed with FlowJo (v.10) software.

RAW 264.7 cells were treated following the same procedure as described above. After 24 h, the cell culture supernatant was collected and centrifuged at 2000 rpm for 20 min to remove dead cells and contaminating proteins. Then, the levels of TNF‑α, IL‑6, Arginase‑1, and IL‑10 in RAW 264.7 cells were determined using ELISA kits.

**Text S6. ROS scavenging capacity**

The H_2_O_2_ scavenging capacity was determined by mixing 10 mL of 1 mM H_2_O_2_ with Musc@CP. 2 h later, 50 μL of the supernatant was mixed for 30 min with 100 μL of Ti(SO_4_)_2_ solution. The 405 nm absorbance was measured. The ability to scavenge •O_2_^-^ was evaluated by calculating the inhibition ratio of NBT photoreduction. Under a constant light intensity, NBT (75 μM), methionine (12.5 mM), riboflavin (20 μM), and Musc@CP were combined for 10 min. The solution’s full wavenumber scanning curve was detected, and the solution’s absorbance at 560 nm was recorded.

**Text S7. Live/dead staining of bacteria**

Bacteria were cultured to the logarithmic growth phase, and an appropriate amount of bacterial suspension was taken and centrifuged (3000 rpm, 10 min) to collect the bacteria. The bacteria were washed with PBS 2-3 times and resuspended in PBS, adjusting the bacterial suspension concentration to 10^8^ CFU mL^-1^. After 24 h of group treatment, the bacterial suspensions were collected. Meanwhile, the working solution of DAMO/PI staining agent was prepared according to the kit ratio. According to the ratio of 1 μL staining working solution (100 ×) per 100 μL bacterial suspension, the solutions were mixed. The mixture was incubated at 37°C in the dark for 15 min. After incubation, 10 μL of the bacterial suspension was dropped onto a glass slide and covered with a 24 mm square coverslip. The staining effect was observed under a fluorescence microscope. DMAO emits green fluorescence with Ex/Em = 503/530 nm; PI emits red fluorescence with Ex/Em = 535/617 nm.

**Text S8. Animal Study**

All animals were housed in a specific pathogen-free (SPF) animal facility for 2 weeks for environmental adaptation and allowed free access to food and water. During the experiment, all animals were kept in the same standard environment (23-26°C, 40-60% humidity, 12 h light-dark cycle, and five mice/cage). All procedures, including animal care, wound modeling, dosing, and termination, were performed according to the Experimental Animal Guidelines for Ethical Review of Animal Welfare (GB/T 35892-2018) and approved by the National Center for Nanoscience and Technology Institute Animal Ethics Committee with the assigned approval number NCNST21-2408-0421. The mice were anesthetized with isoflurane anesthetic before any procedure that would cause pain. After the experiment, the mice were euthanized by CO_2_ inhalation followed by cervical dislocation.

**Text S9. Establishment of an infected diabetic wound model**

Male C57BL/6J mice weighing 25-30 g were fasted for 12 h, followed by intraperitoneal injection of streptozotocin (50 mg kg^-1^), and the process was repeated five times within 5 days. The mice were kept under anesthesia during intraperitoneal injection. All mice were provided with 10% sucrose water. Two weeks after the fifth injection, diabetic mice were identified as having blood glucose levels exceeding 16.1 mmol L^-1^ for two consecutive measurements within 2 days. The diabetic mice were anesthetized with isoflurane anesthetic, and their back fur was completely shaved. Then, 10 mm diameter skin biopsy punches were used to create full thickness wounds, and 10 μL of MRSA suspension (1 × 10^7^ CFU mL^-1^) was dropped on the surface of the wounds to create an infected wound model. The mice were kept under anesthesia during this procedure. After 1 day of infection, the number of bacterial colonies increased from 10^5^ to 10^6^ CFUs, indicating that the infection model was successfully established.

**Text S10. Diabetic wound healing**

**Infected Diabetic wound healing:** The wound was treated with PBS as the control group. Subsequently, every 3 or 4 days, the mice were anesthetized with isoflurane and the wound macroscopic images were taken after replacement. To visually analyze the antibacterial effect of Musc@CP, the skin at the wound site was collected and homogenized on the 4th day. The homogenate was serially diluted and spread on LB agar plates to quantify the bacterial colonies. On the 4th, 7th and 13th days, the mice were euthanized by inhalation of carbon dioxide followed by cervical dislocation. Among them, the skin tissue on the 4th day was collected for grinding, then cultivated the bacteria and observed the antibacterial effects of different treatment groups. The skin tissue on the 13th day was collected and stained with H&E and MST. Rete ridges were quantified on Masson’s trichrome-stained wound tissue sections harvested on day 13. The region of interest was defined as the dermal-epidermal junction. For each section, the total area occupied by rete ridge structures was measured using Image J and then normalized to a defined linear length (1 mm) along the wound surface. The result was expressed as the rete ridge area per millimeter of wound length. To evaluate the biocompatibility and biosafety of Musc@CP, on the 14th day after Musc@CP treatment, as the wound healed, the edges of the dressing gradually separated from the healed wound tissue and the dressing was slowly removed. The main organs of the mice, including the heart, liver, spleen, lung and kidney, were collected for H&E staining analysis.

**Non-infected Diabetic wound healing:** For non-infectious diabetic mouse models, the wound was treated with PBS as the control group. Subsequently, every 3 or 4 days, the mice were anesthetized with isoflurane and the wound macroscopic images were taken after replacement. On the 3rd, 9th and 12th days, the mice were euthanized by inhalation of carbon dioxide followed by cervical dislocation. Among them, the skin tissue on the 3rd day was collected and analyzed for angiogenesis and macrophage polarization at the wound site by immunofluorescence staining of CD31/*α*-SMA and iNOS/CD206. The skin tissue on the 9th day was collected and stained with H&E and MST. To evaluate the biocompatibility and biosafety of Musc@CP, on the 12th day after Musc@CP treatment, as the wound healed, the edges of the dressing gradually separated from the healed wound tissue and the dressing was slowly removed. The main organs of the mice, including the heart, liver, spleen, lung and kidney, were collected for H&E staining analysis.

**
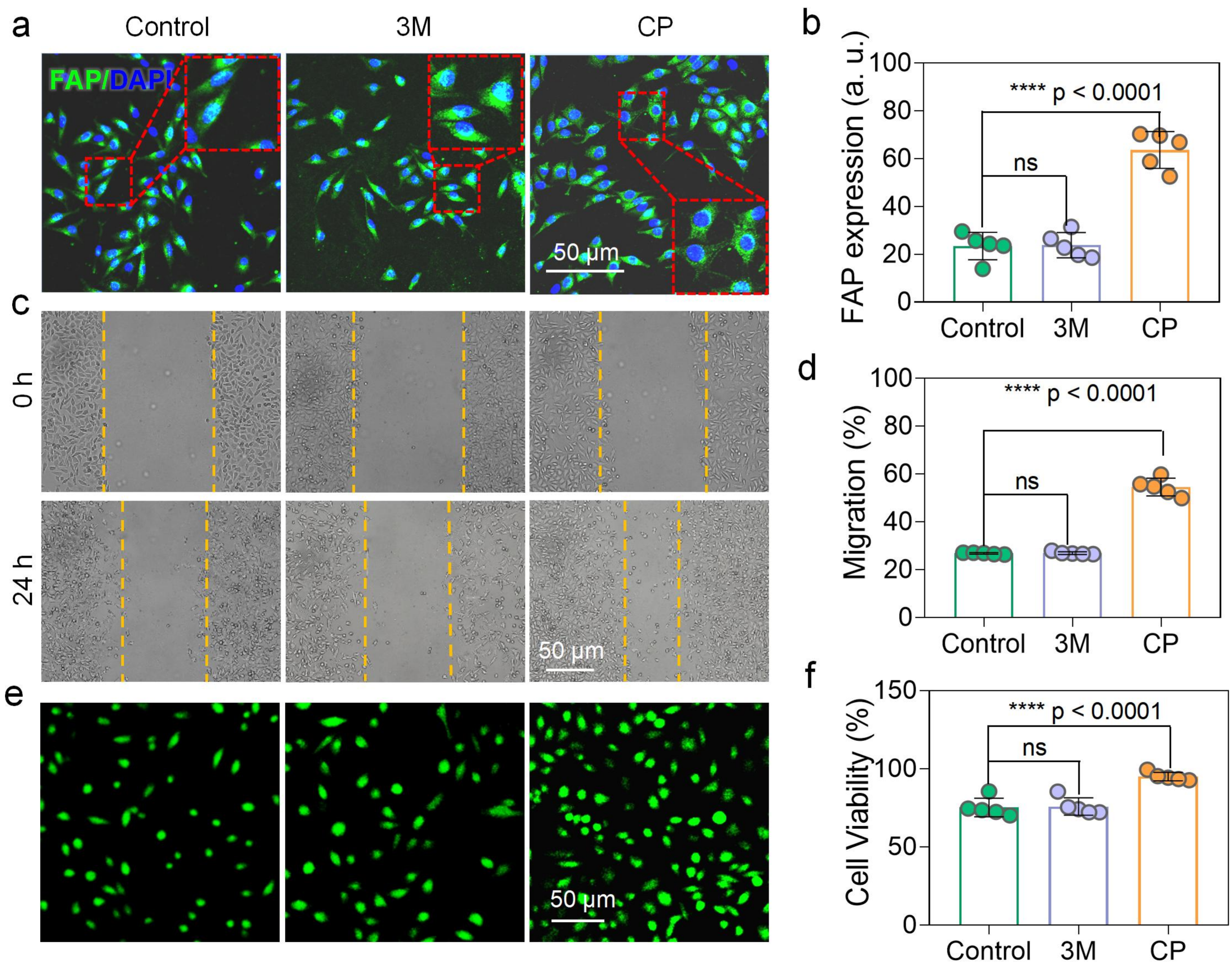
**

**Extended Data Figure S1.** ECM-mimetic CP scaffold promotes fibroblast activation, migration and proliferation. (a) Representative images of FAP staining and (b) quantitative analysis of the mean intensity of FAP in L929 cells. (c) Images and (d) quantification of the migration of L929 cells. (e, f) Proliferation of 3T3 cells after different treatments for 72 h. Each experiment was repeated independently three times with similar results. The data are presented as the mean ± s.d. (n = 5 biologically independent cells).

**
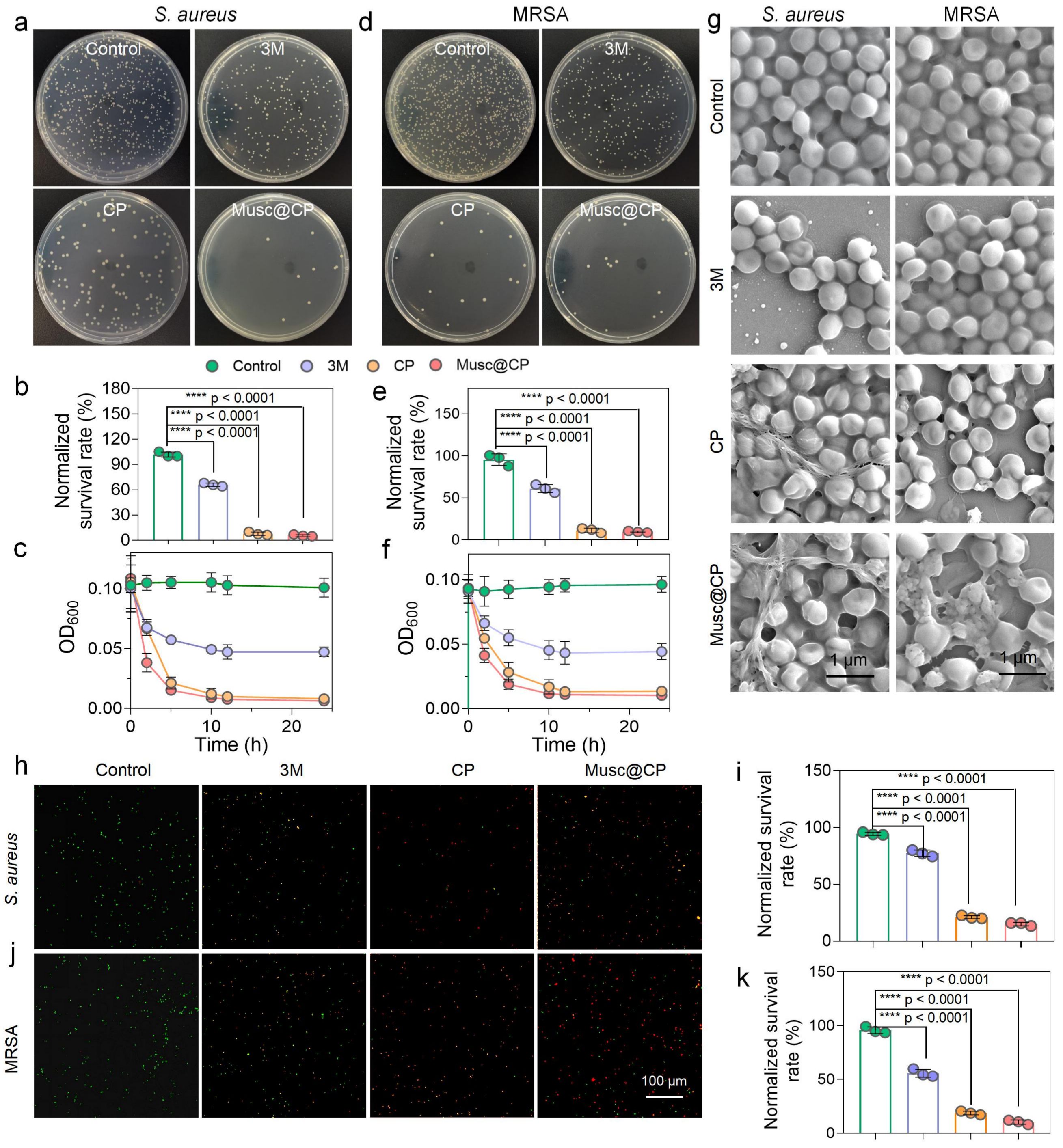
**

**Extended Data Figure S2.** Evaluation of the antibacterial performance of Musc@CP. (a) Representative culture images and (b) the relative amounts of *S. aureus* after different group treatments (LB agar, 37°C, 24 h). (c) OD_600_ of *S. aureus* after different group treatments. (d) Representative culture images and (e) the relative amounts of MRSA after different group treatments (LB agar, 37°C, 24 h). (f) OD_600_ of MRSA under different group treatments. (g) Representative SEM images of *S. aureus* and MRSA under different group treatments. Representative images of live/dead staining of (h) *S. aureus* and (j) MRSA after different group treatments. Quantificatifying survival rate of (i) *S. aureus* and (k) MRSA after different group treatments.


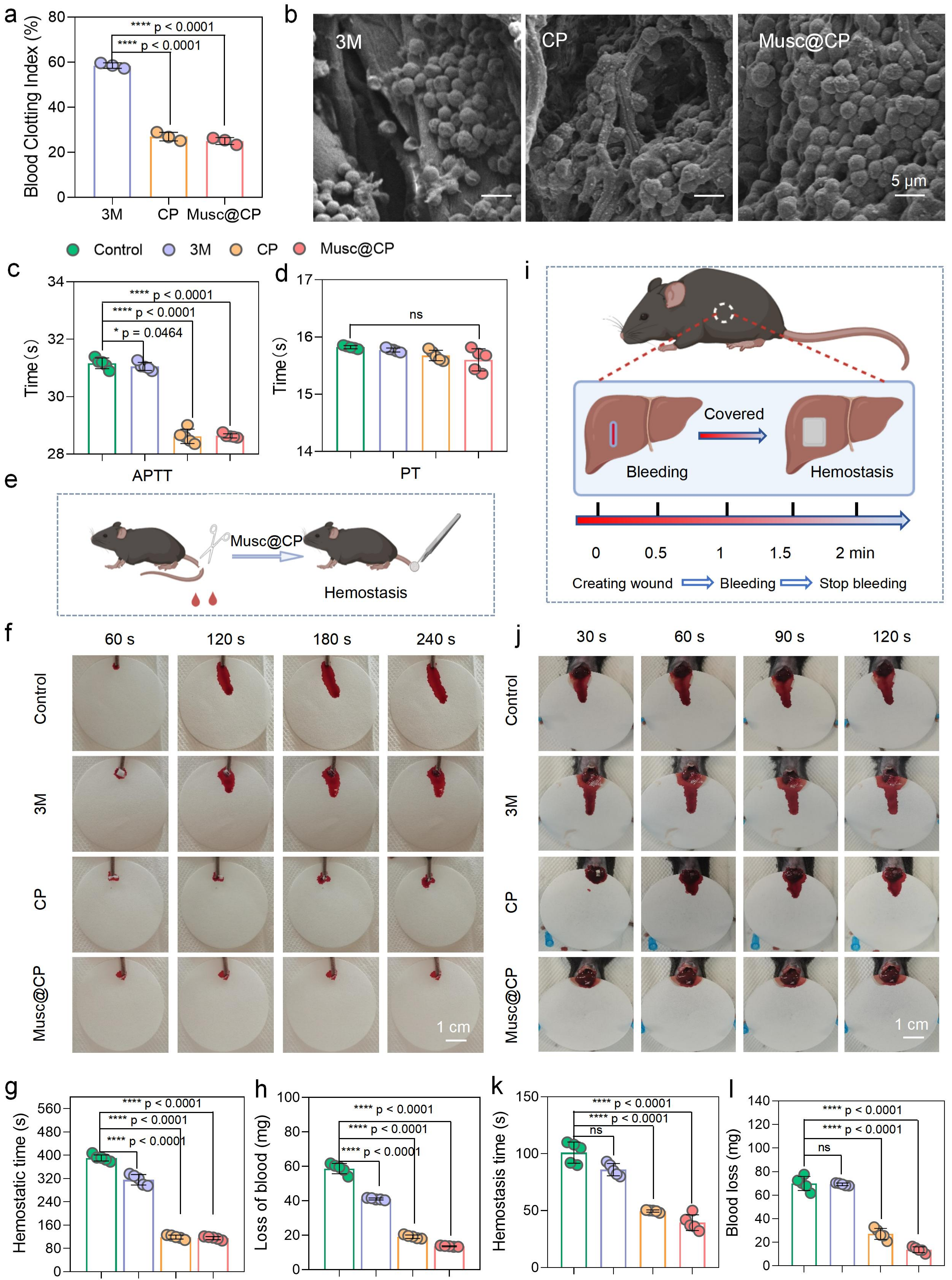


**Extended Data Figure S3.** Hemostatic properties of Musc@CP in diabetic mice. (a) Effect of scaffolds on the blood-clotting index within 60 s. (b) SEM of whole blood contacted with different scaffolds (one biologically independent sample in each group). Results of blood coagulation analysis of the serum from diabetic mice in each group: (c) analysis of APTT and (d) analysis of PT analysis. (e) Schematic diagram of the mouse tail perforation model and the hemostasis process. (f) Photographs of blood loss absorbed by filter paper in the mice tail bleeding model. Effect of different scaffolds on (g) hemostasis time and (h) blood loss in the diabetic mice tail bleeding model. i) Schematic diagram of the mouse liver perforation model and the hemostasis process. j) Photographs of blood loss absorbed by filter paper in the diabetic mice liver bleeding model. Effect of Musc@CP on (k) hemostasis time and (l) blood loss in the diabetic mice liver bleeding model (n = 5 mice in each group).


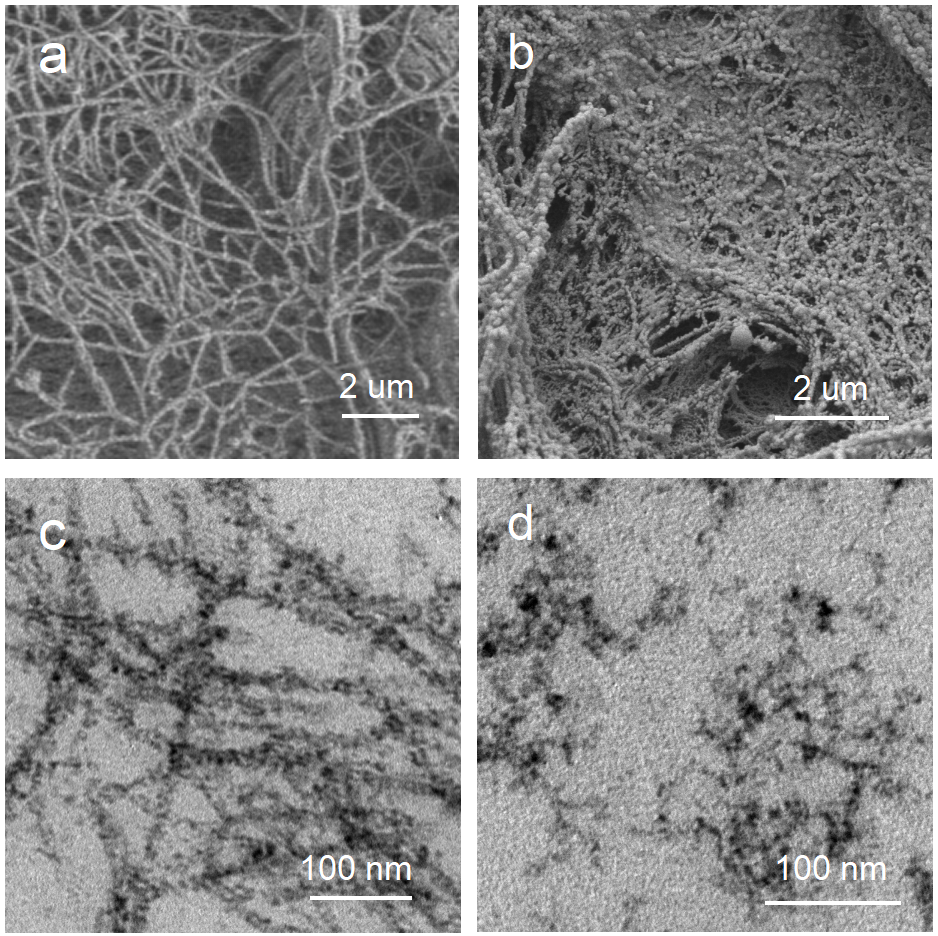


**Figure S1.** (a) SEM images of the reticular fibers in the extracellular matrix of normal mouse skin and (b) diabetic mouse skin. (c) TEM images of the reticular fibers in the extracellular matrix of normal mouse skin and (d) diabetic mouse skin.


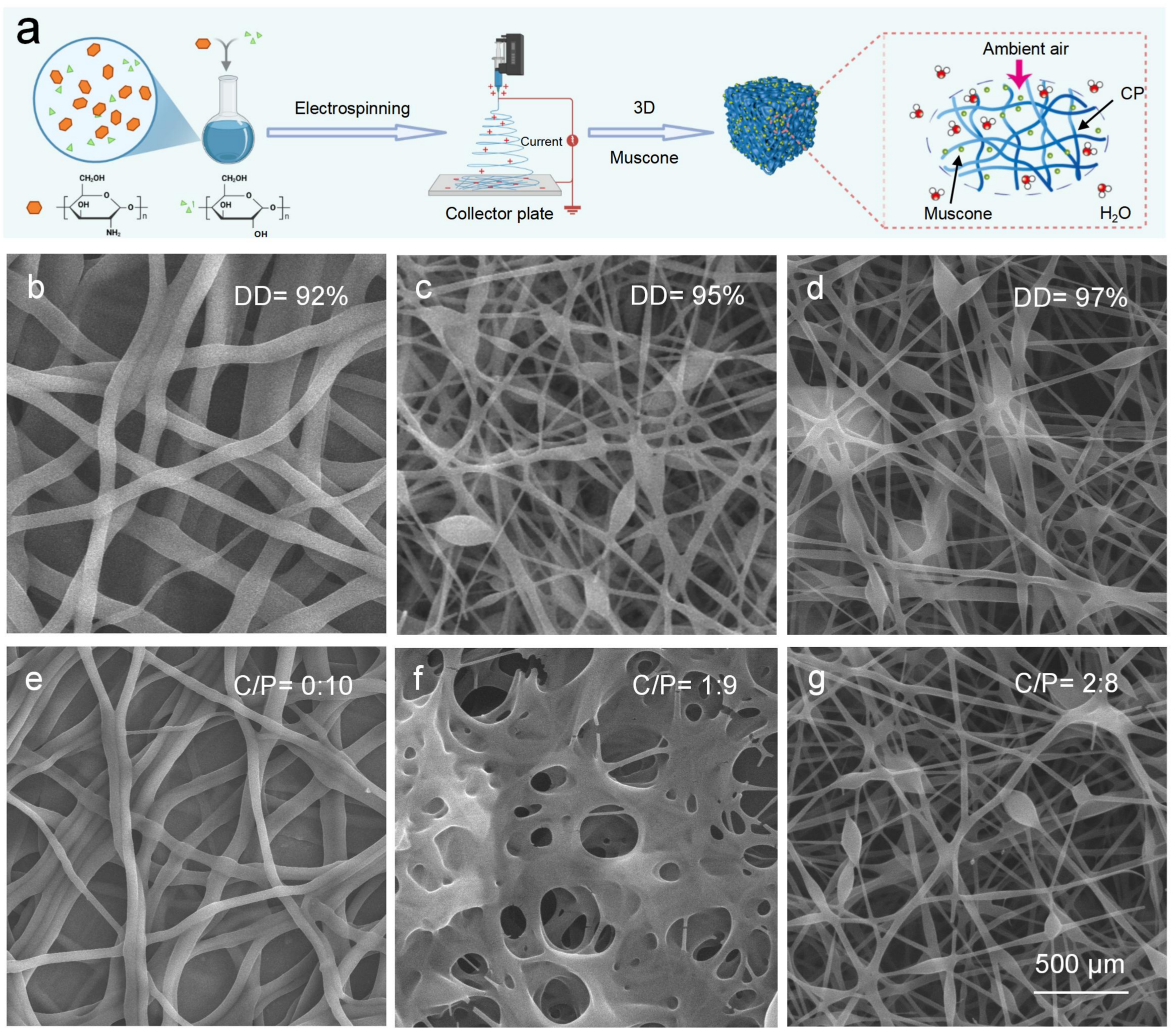


**Figure S2.** Optimization of parameters for electrospun CP nanofibers. (a) Schematic diagram of preparing CP by electrospinning and loading Musc to prepare Musc@CP. The SEM images of CP nanofibers (b-d) at increased DD levels (92%, 95%, 97%) and (e-g) varying C/P (0:10, 1:9, 2:8).

The degree of deacetylation (DD) is positively correlated with solution viscosity, cationic charge density, and antibacterial efficacy, yet it concurrently impedes the formation of uniform nanofibers. Similarly, an increased chitosan-to-pullulan (C/P) ratio elevates viscosity and often leads to beaded fiber morphology, whereas a lower ratio compromises spinnability. Notably, pure pullulan (100% P) yields highly uniform but extremely fine fibers that lack mechanical robustness and are prone to fracture.

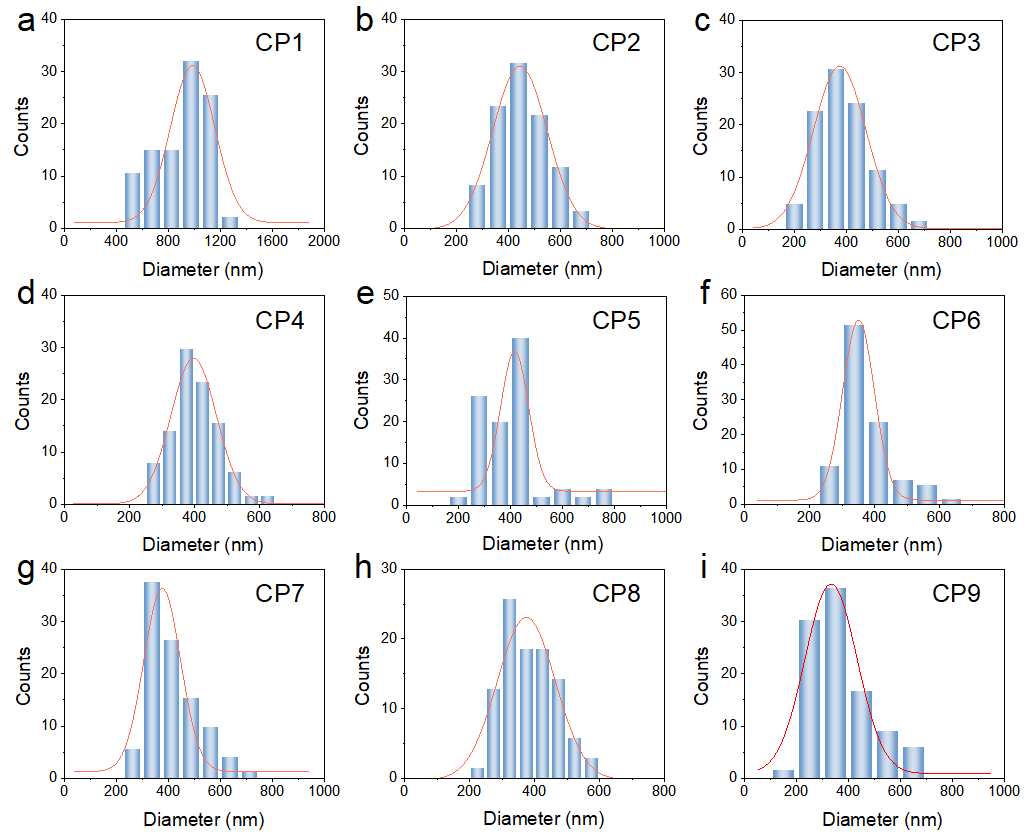


**Figure S3.** (a-i) Fiber diameter distribution of CP in 9 orthogonal experiments.

Statistical analysis of fiber diameter distributions revealed that CP2, CP3 and CP4 all exhibited a normal distribution. Among these, CP3 demonstrated a diameter profile most closely aligned with our target specifications.


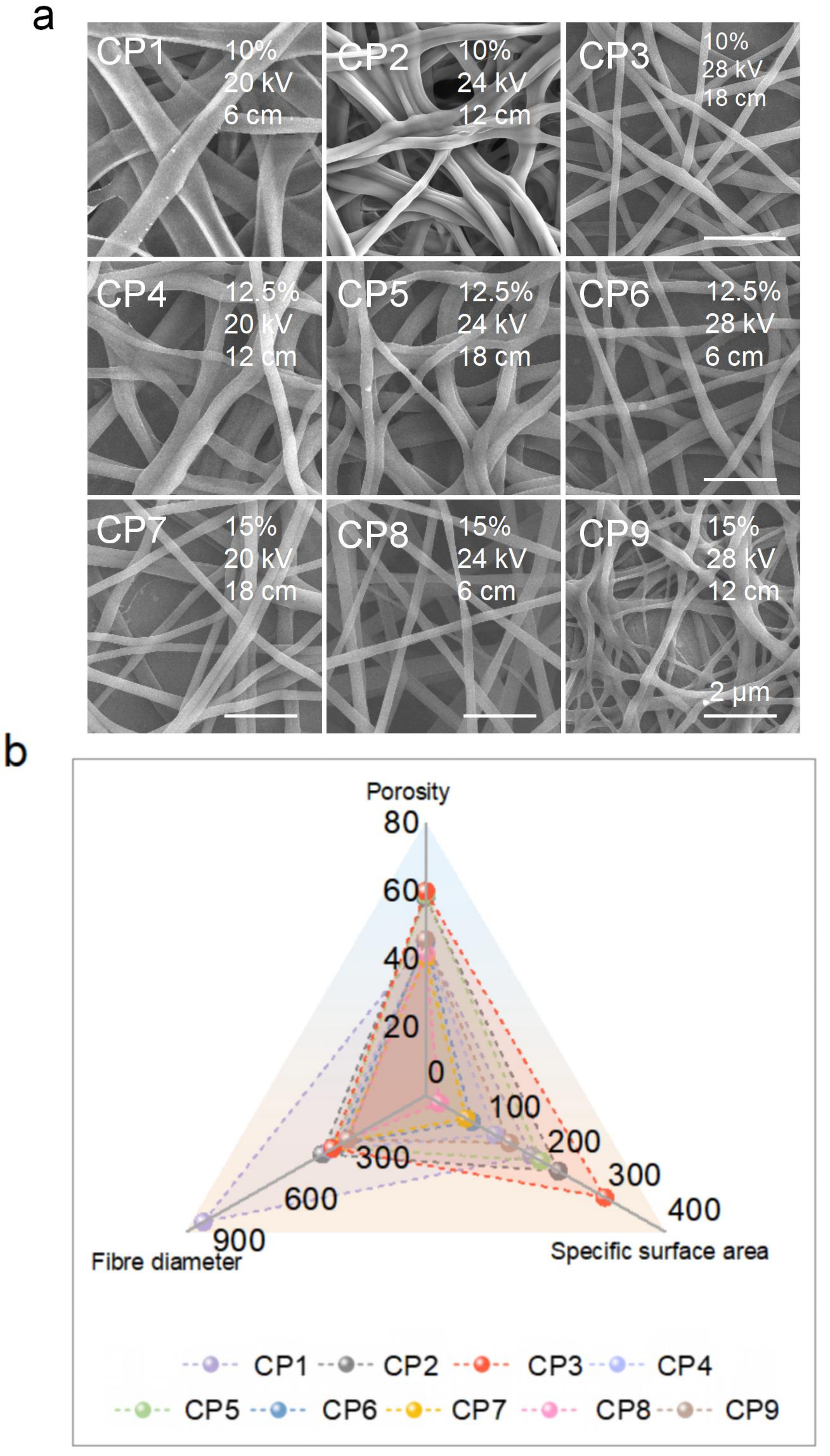


**Figure S4.** (a) Representative SEM images of Musc@CP fabricated under varying processing conditions. (b) Radar chart comparing the performance metrics (porosity, specific surface area and fiber diameter) across nine experimental groups (CP1-CP9).

Taking into account the critical structural parameters of fiber diameter, surface area, and porosity, CP3 was identified as the optimal formulation and was advanced for further study.

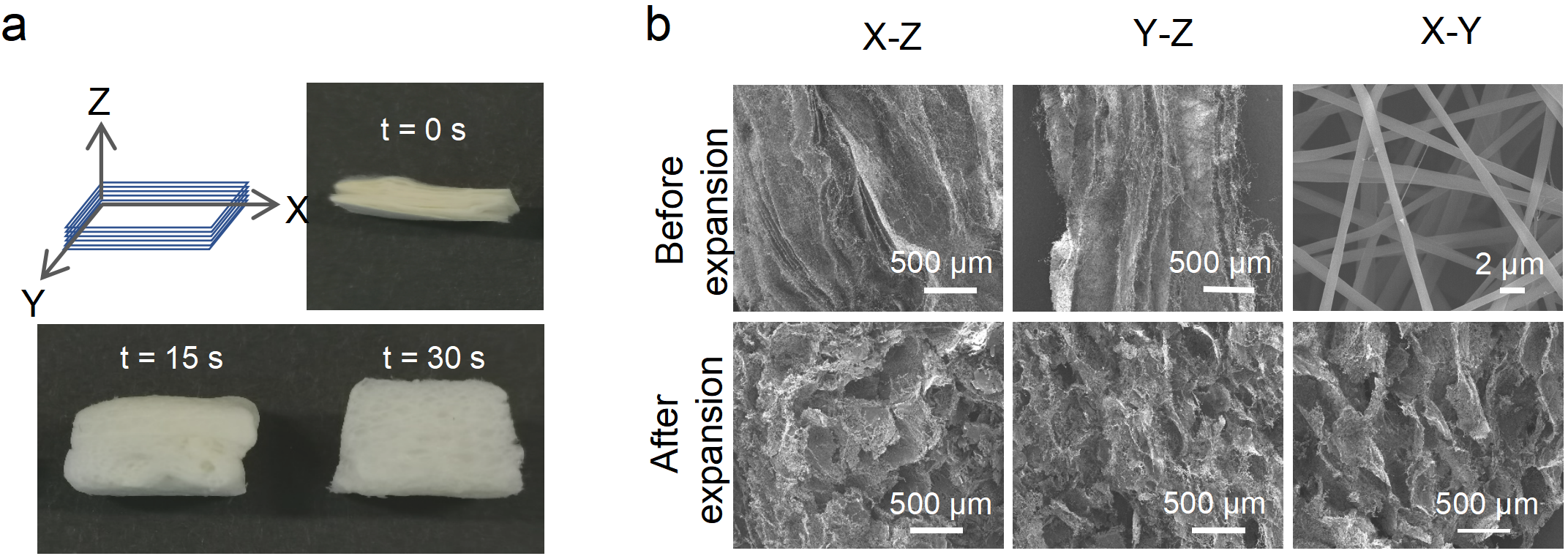


**Figure S5.** Microstructure of CP nanofibers. (a) The thickness of CP increased as extending the foaming time. (b) SEM of corresponding surfaces including before expansion and after expansion.

Diabetic wounds are frequently complicated by excessive exudate. To address this clinical need and enhance the dressing’s utility, we further engineered the material into a three-dimensional porous architecture via a foaming process. This structural optimization significantly increased thickness and fluid-uptake capacity without compromising the surface topography. As expected, extending the foaming time progressively augmented material thickness (Supplementary Fig. 5a).

To ensure the CP scaffold retained its ECM-mimetic structural features, we selectively immersed only the lower portion of the scaffold in the foaming solution, thereby generating a porous sublayer without compromising the nano-topographical integrity of the upper surface. SEM confirmed the uniform fibrous surface prior to foaming, while cross-sectional views from three orthogonal planes after foaming revealed a well-interconnected porous network throughout the scaffold (Supplementary Fig. 5b).


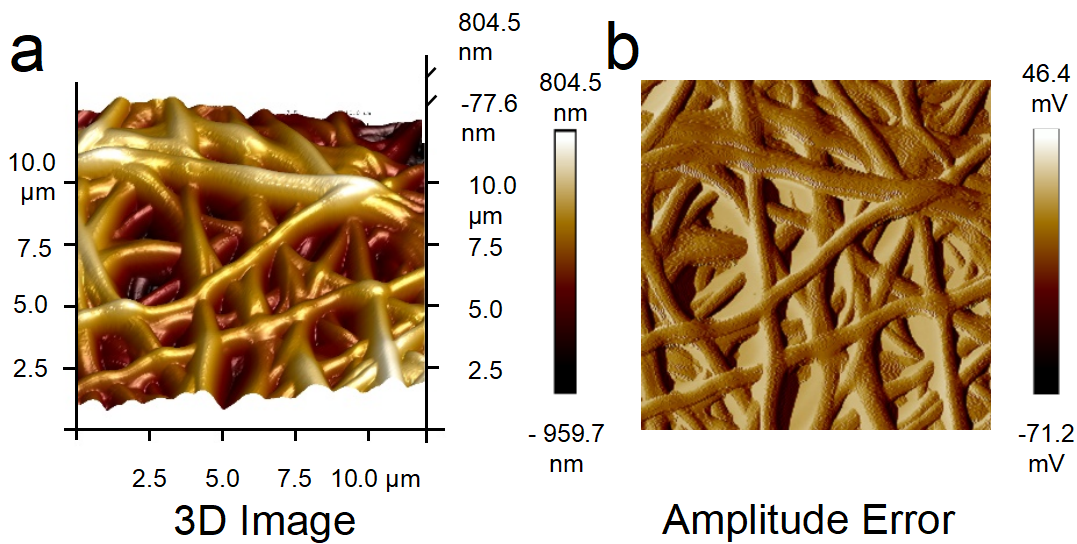


**Figure S6.** (a) AFM images of Musc@CP nanofiber about 3D image and (b) amplitude error.


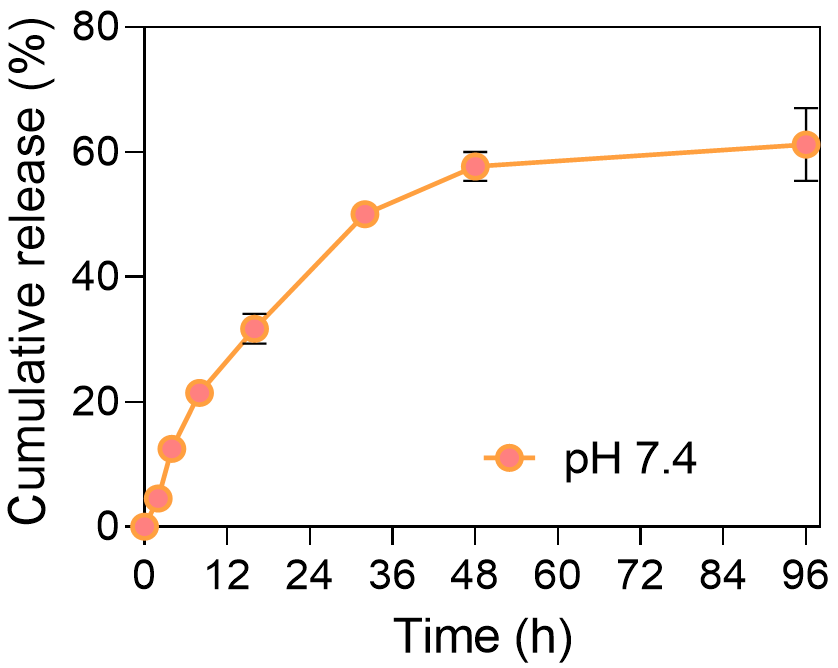


**Figure S7.** Cumulative release of muscone from the Musc@CP scaffold at pH 7.4.


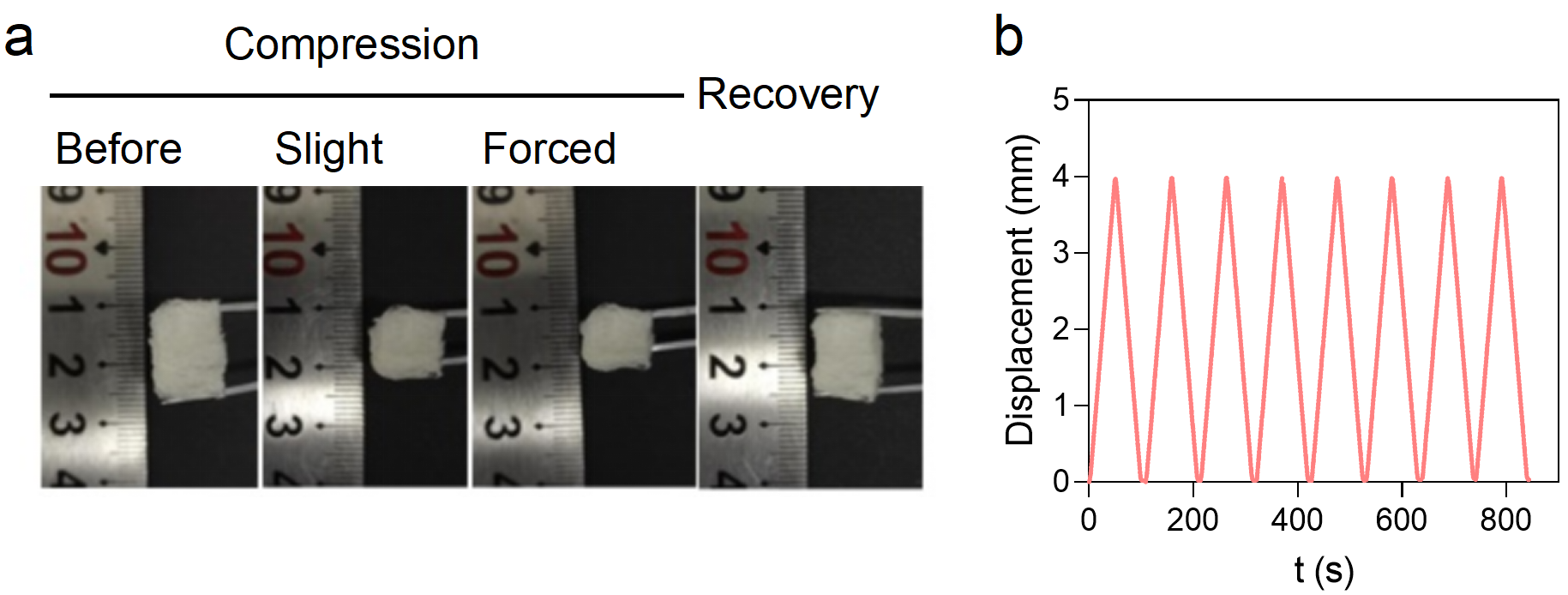


**Figure S8.** Mechanical resilience of Musc@CP. (a) Macroscopic recovery documentation after compression. (b) Displacement evolution during cyclic compression.


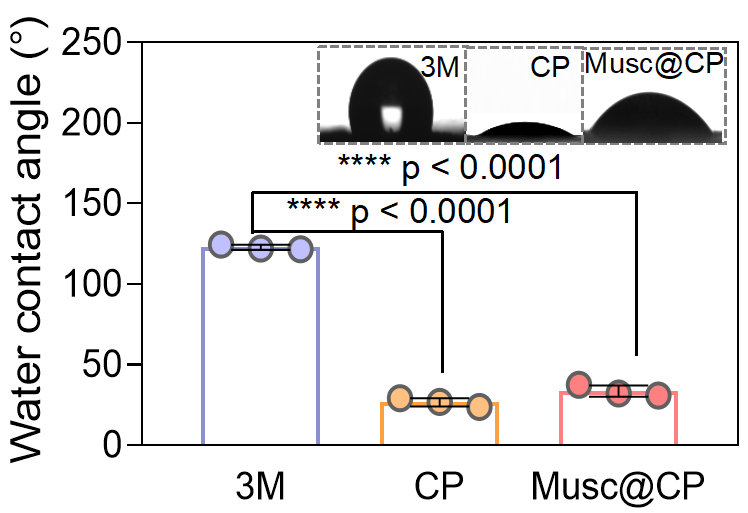


**Figure S9.** Water contact angle of different scaffolds.

Compared to the pristine CP scaffold, Musc@CP exhibited a slight decrease in hydrophilicity ratio, which we attribute to the hydrophobic nature of the surface-loaded Musc.


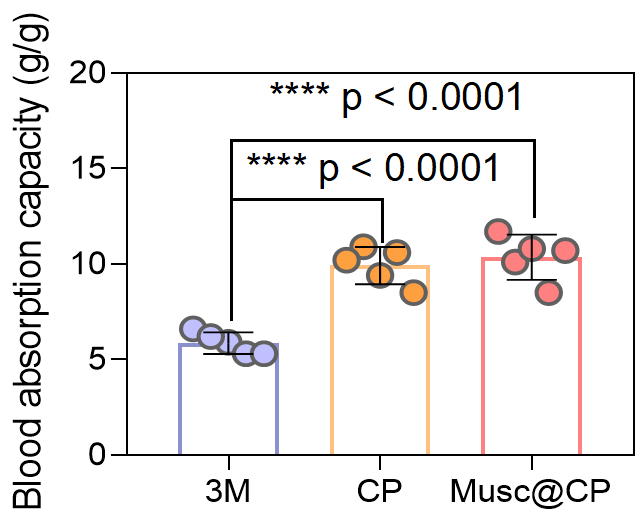


**Figure S10.** Blood absorption capacity of different scaffolds. CP and Musc@CP.

Both CP and Musc@CP have demonstrated significantly superior blood absorption capacity compared to the 3M.


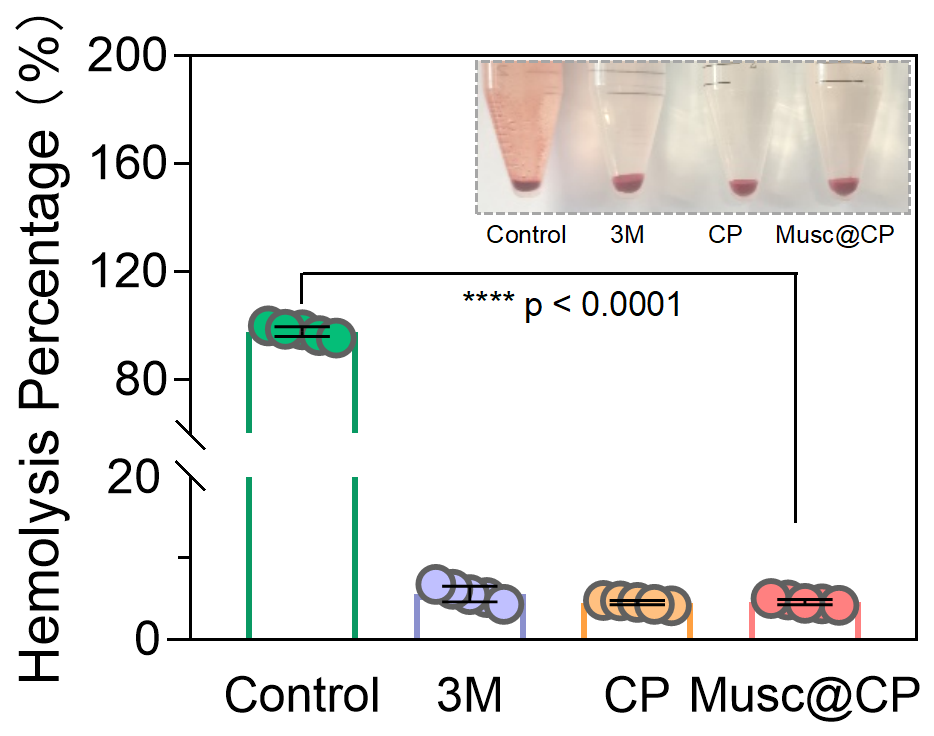


**Figure S11.** Hemolysis assay and images (insert) of RBCs incubated with different scaffolds (5 mg mL^-1^).

The percentage of RBC lysis was < 5% for Musc@CP.


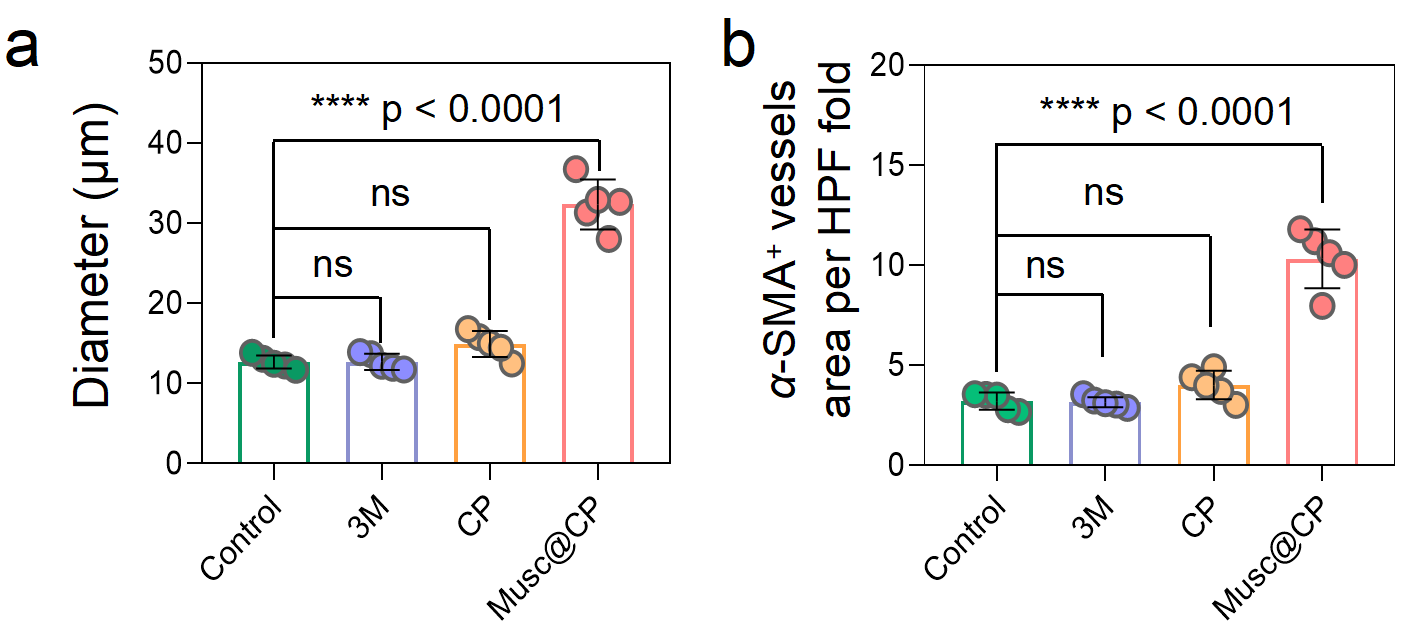


**Figure S12.** (a) Quantitative analysis of mean vessel diameter of wounds in different treatment groups on day 3. (b) Quantitative analysis of vessel area of wounds in different treatment groups on day 3. n = 5 biological independent samples. n = 5 biological independent samples.


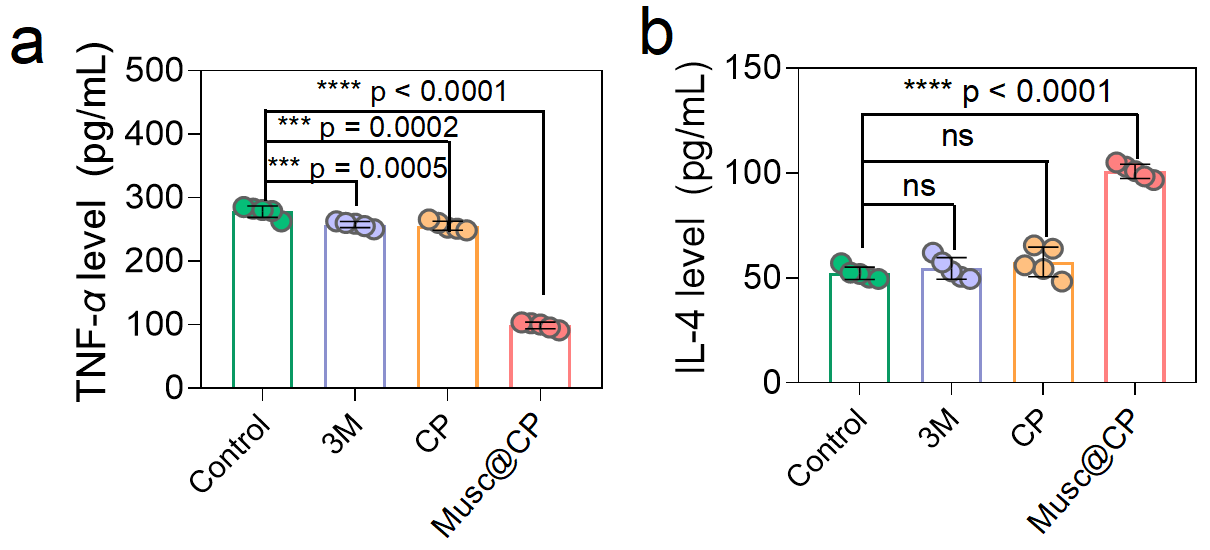


**Figure S13.** (a) ELISA measurements revealed the lowest level of the pro-inflammatory cytokine TNF-*α* in the Musc@CP group. (b) and this group displayed the highest level of the anti-inflammatory cytokine IL-4.

Immunofluorescence staining of sections of wounded tissue on Day 3 showed that the Musc@CP group exhibited the lowest fluorescence intensity of iNOS, this group also displayed the strongest fluorescence signal for CD206 (Fig. 2e-h).

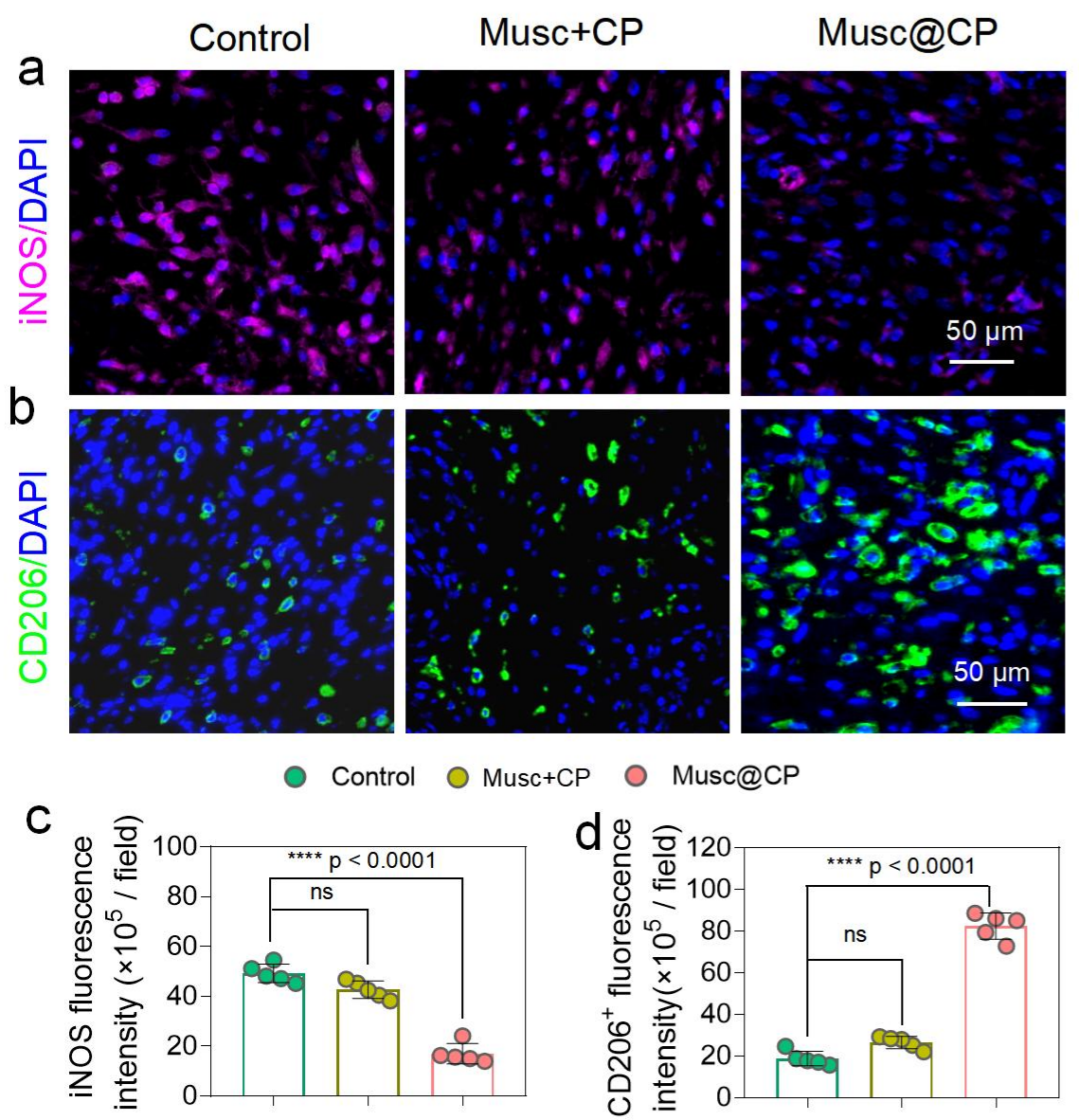


**Figure S14.** (a) iNOS, and (b) CD206 expression in different in vivo treatment groups on day 3. The fluorenscence intensity of (c) iNOS and (d) CD206 in different in vivo treatment groups on day 3. (n = 5 independent cells).

To distinguish between sustained release from nanofibers and simple physical mixing, we added a control group consisting of CP scaffold plus free muscone (Musc+CP) administered at the same total dose as loaded in Musc@CP. Immunofluorescence staining (Fig. S14) showed that the Musc+CP group exhibited a slight decrease in iNOS expression and a slight increase in CD206 expression compared to the Control group, but both were significantly inferior to the Musc@CP group.


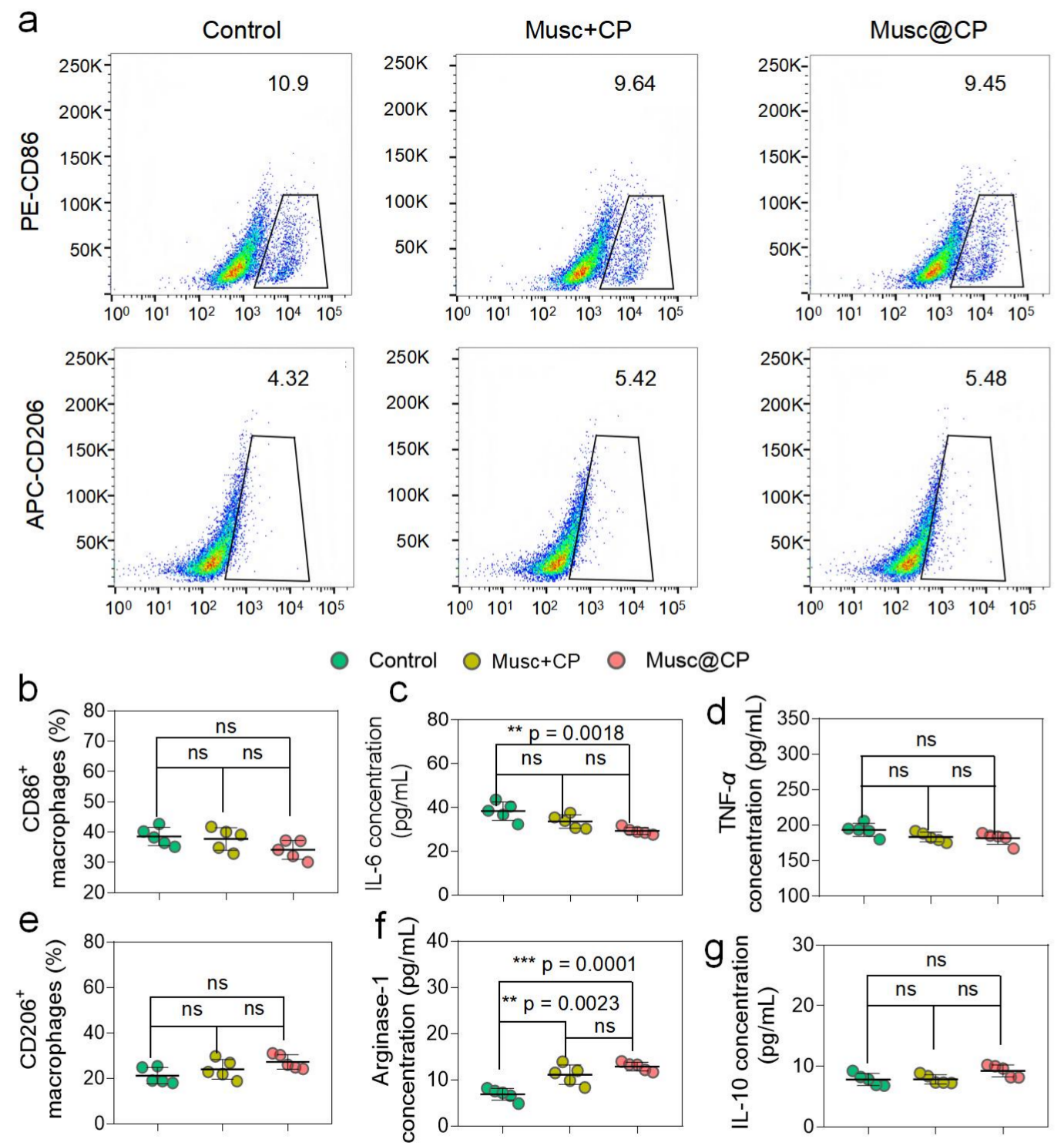


**Figure S15.** (a) Flow cytometry analysis of macrophage polarization following different treatments. Each experiment was repeated independently five times with similar results. (b) Flow cytometry and quantification analysis of M1 macrophages (CD86^+^). ELISA results showing the relative levels of pro-inflammatory cytokines (c) IL 6 and (d) TNF-α in RAW264.7 cells following different treatments. (e) Flow cytometry and quantification analysis of M2 macrophages (CD206^+^). ELISA results showing the relative levels of anti-inflammatory cytokines (f) Arginase-1 and (g) IL-10 in RAW264.7 cells following different treatments. n = 5 biological independent samples.

Using flow cytometry, we evaluated the direct effects of CP scaffold and free muscone (Musc+CP), and Musc@CP on macrophage polarization in vitro. The results showed that neither Musc+CP nor Musc@CP significantly promoted M1 to M2 transition compared to the control (Fig. S14). Specifically, the percentage of CD86^+^ (M1) cells showed only a marginal decrease, while CD206^+^ (M2) cells exhibited a slight, non-significant increase. ELISA further confirmed that levels of pro-inflammatory cytokines (IL-6, TNF-α) and anti-inflammatory factors (Arginase-1, IL-10) remained largely unchanged across all treatment groups. These findings indicate that neither the CP scaffold nor muscone (free or loaded) possesses a substantial direct immunomodulatory effect on macrophages in vitro.


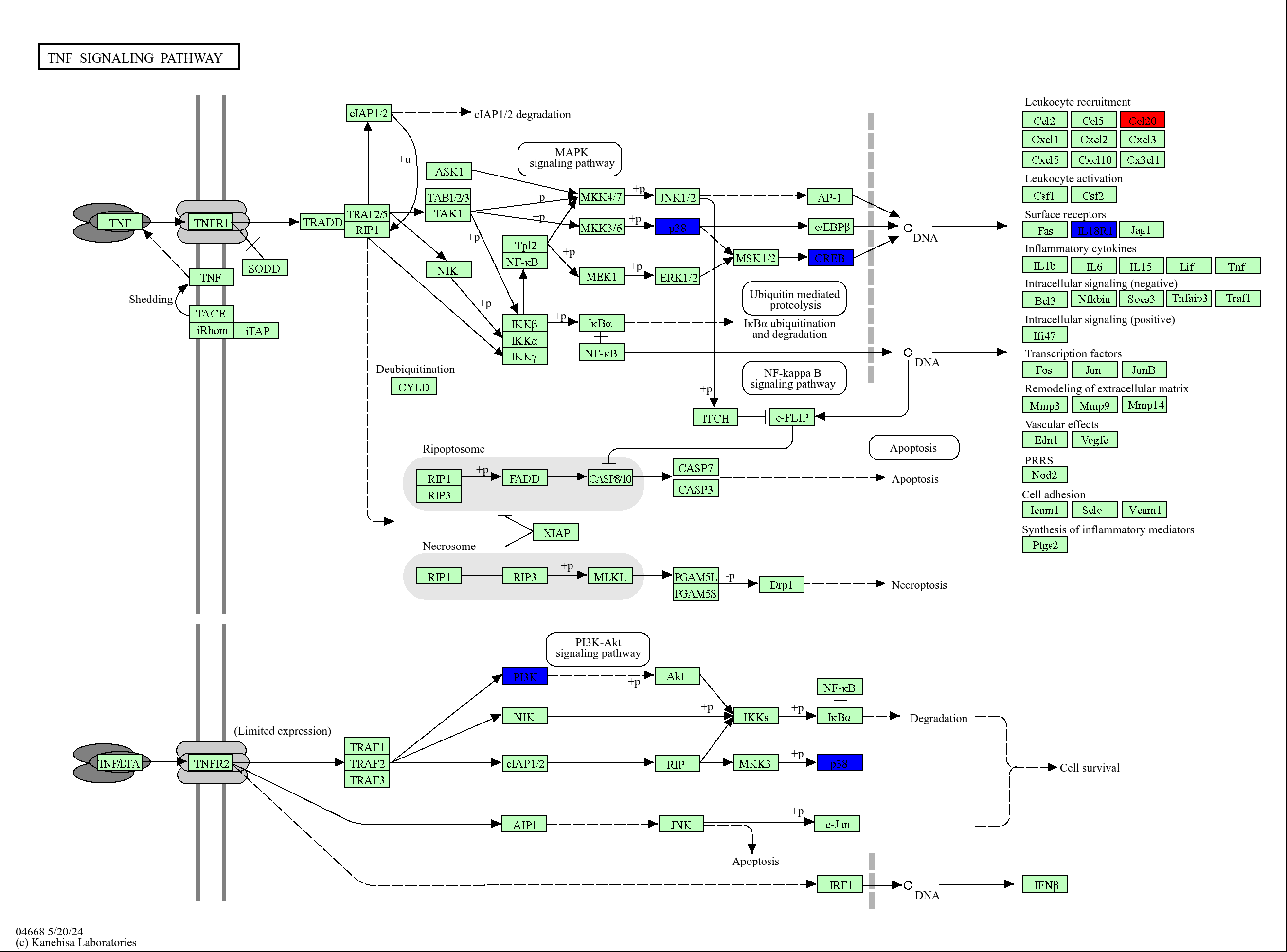


**Figure S16.** Pathview of KEGG terms TNF signaling pathway based on DEGs in Musc@CP group vs Control group.


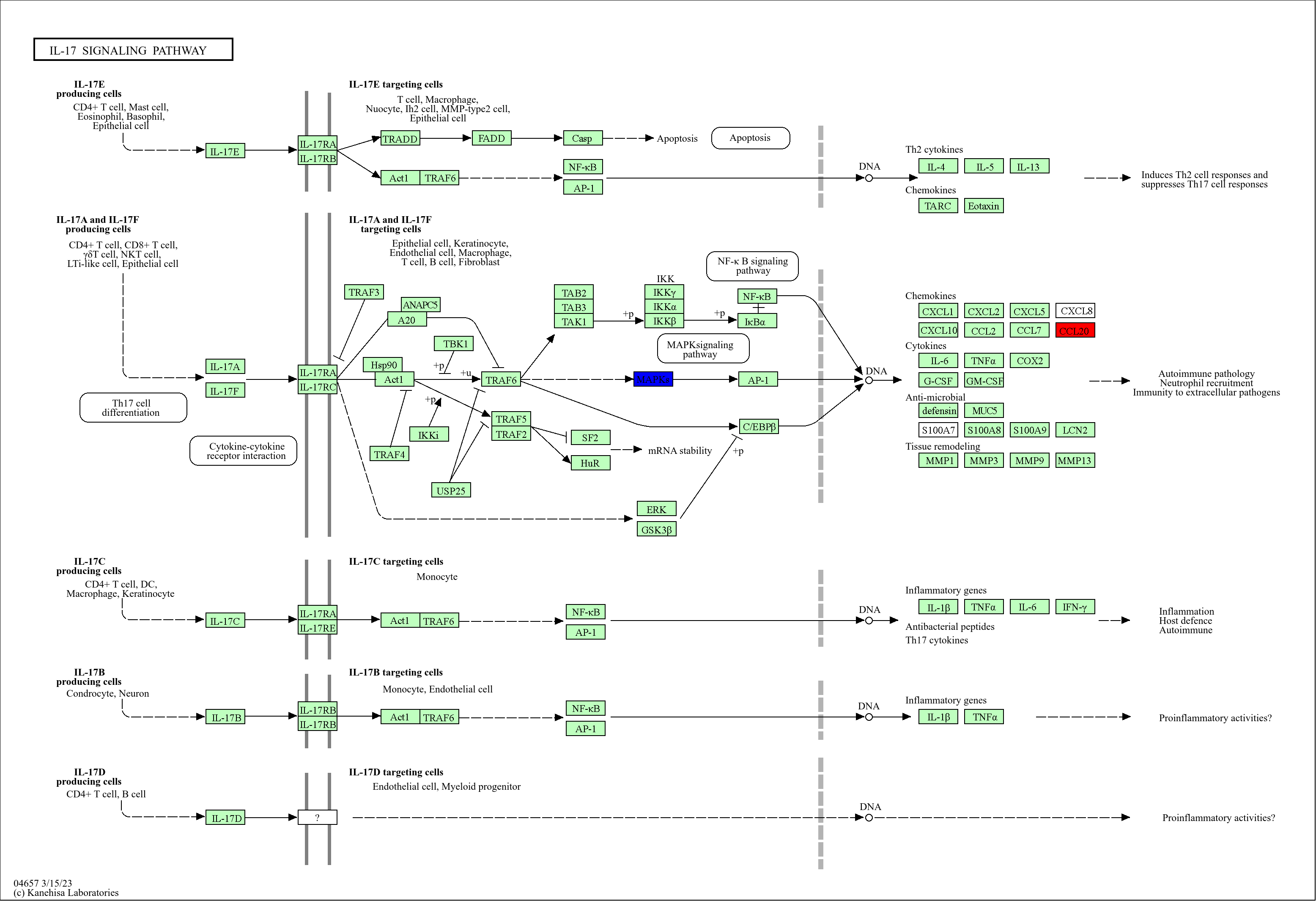


**Figure S17.** Pathview of KEGG terms IL-17 signaling pathway based on DEGs in Musc@CP group vs Control group.


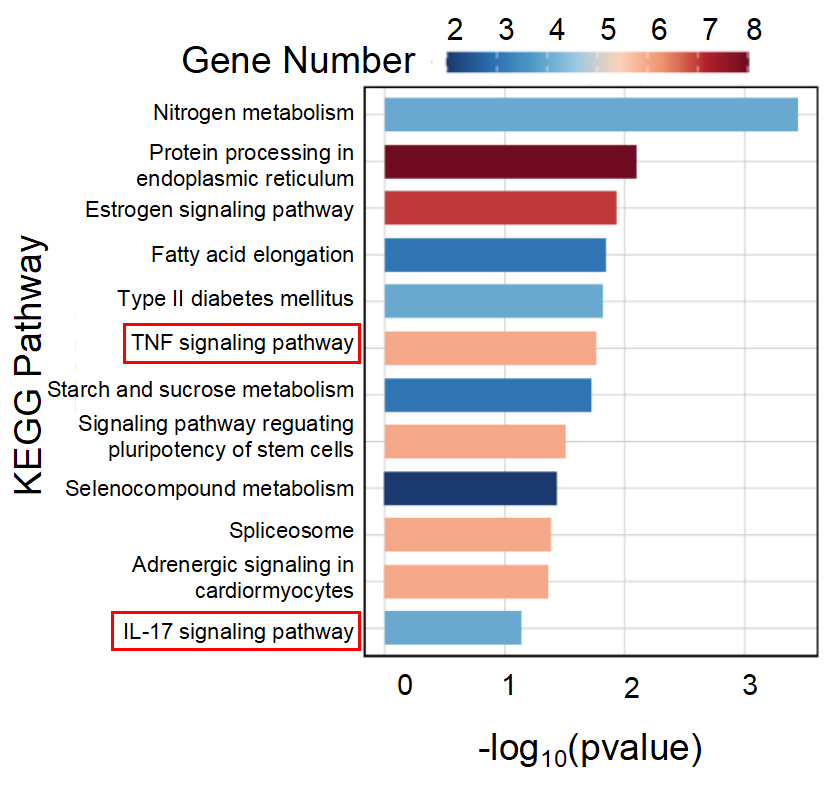


**Figure S18.** KEGG pathway enrichment analysis of DEGs between the Musc@CP and Control groups.

The enrichment analysis of DEGs in KEGG was performed using KOBAS, based on an over-representation analysis algorithm with *p*-values calculated by a hypergeometric test.


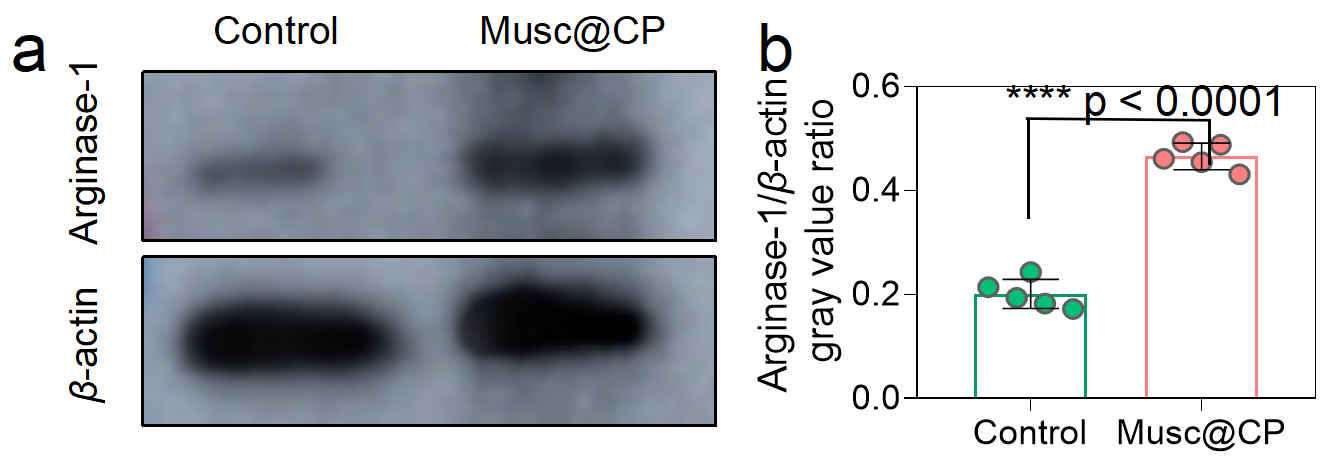


**Figure S19.** (a) Western Blot analysis of Arginase-1 expression in wound tissues of C57BL/6 diabetic mice and (b) quantitative histogram of Arginase-1 levels.


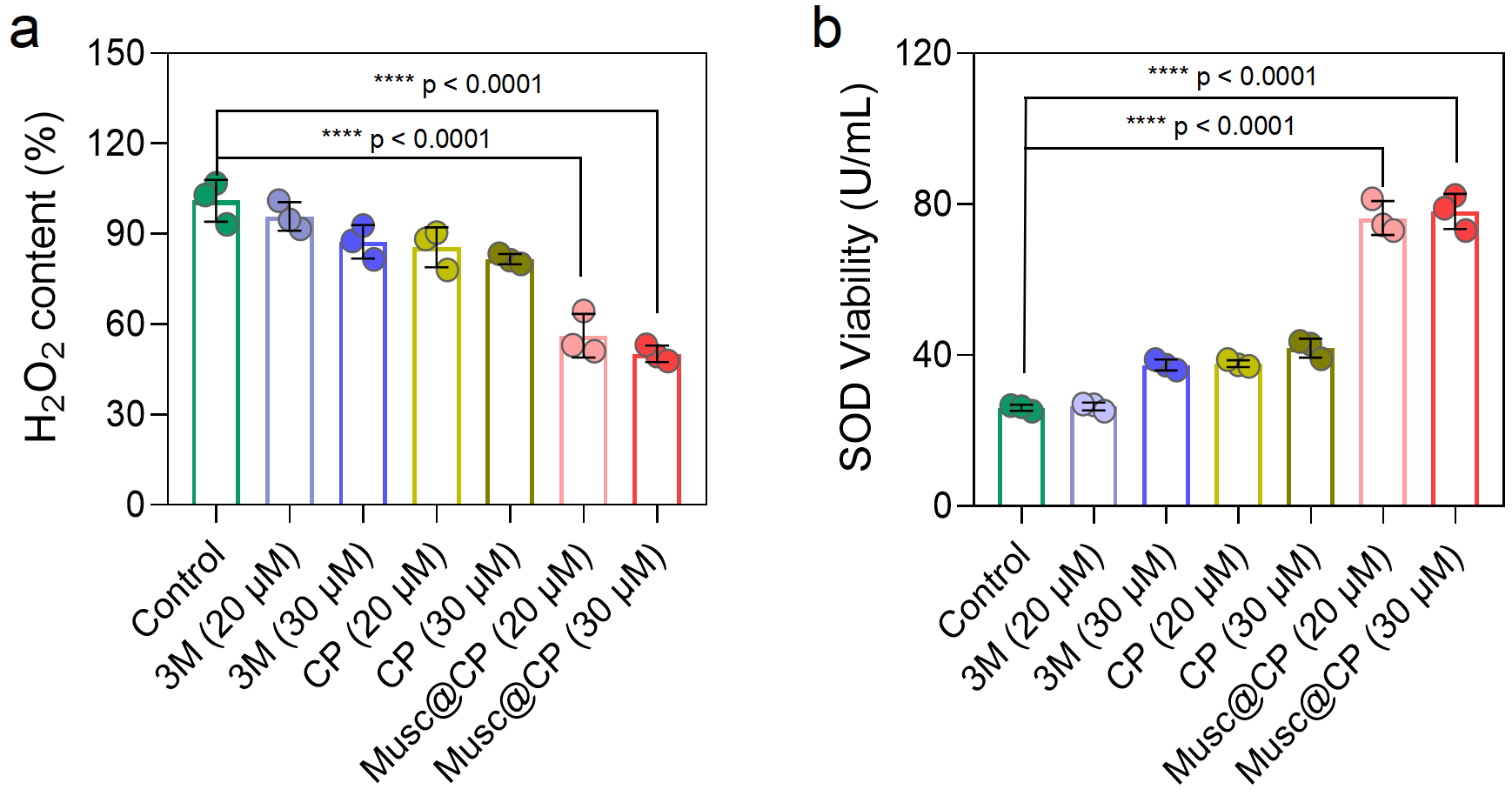


**Figure S20.** (a) H_2_O_2_-scavenging and (b) superoxide dismutase (SOD) viability performance in response to various treatments. The data are presented as the mean ± s.d. (n = 3 independent cells). Statistical differences were analyzed by Student’s two-sided t-test.

SOD is an antioxidant enzyme that catalyzes the dismutation (or disproportionation) of the superoxide anion radical (•O_2_^-^) into ordinary molecular oxygen (O_2_) and hydrogen peroxide (H_2_O_2_). By removing superoxide radicals, SOD plays a critical role in protecting cells from oxidative stress and damage.


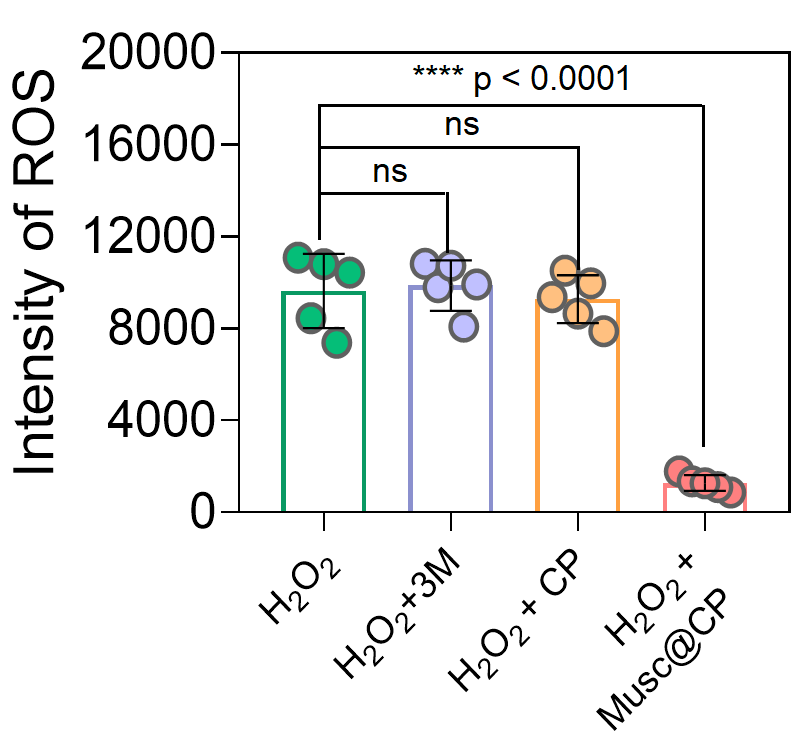


**Figure S21.** ROS scavenging ability of different scaffolds in L929. Each experiment was repeated independently three times with similar results. The data are presented as the mean ± s.d. (n = 5 independent cells). Statistical differences were analyzed by Student’s two-sided t-test.


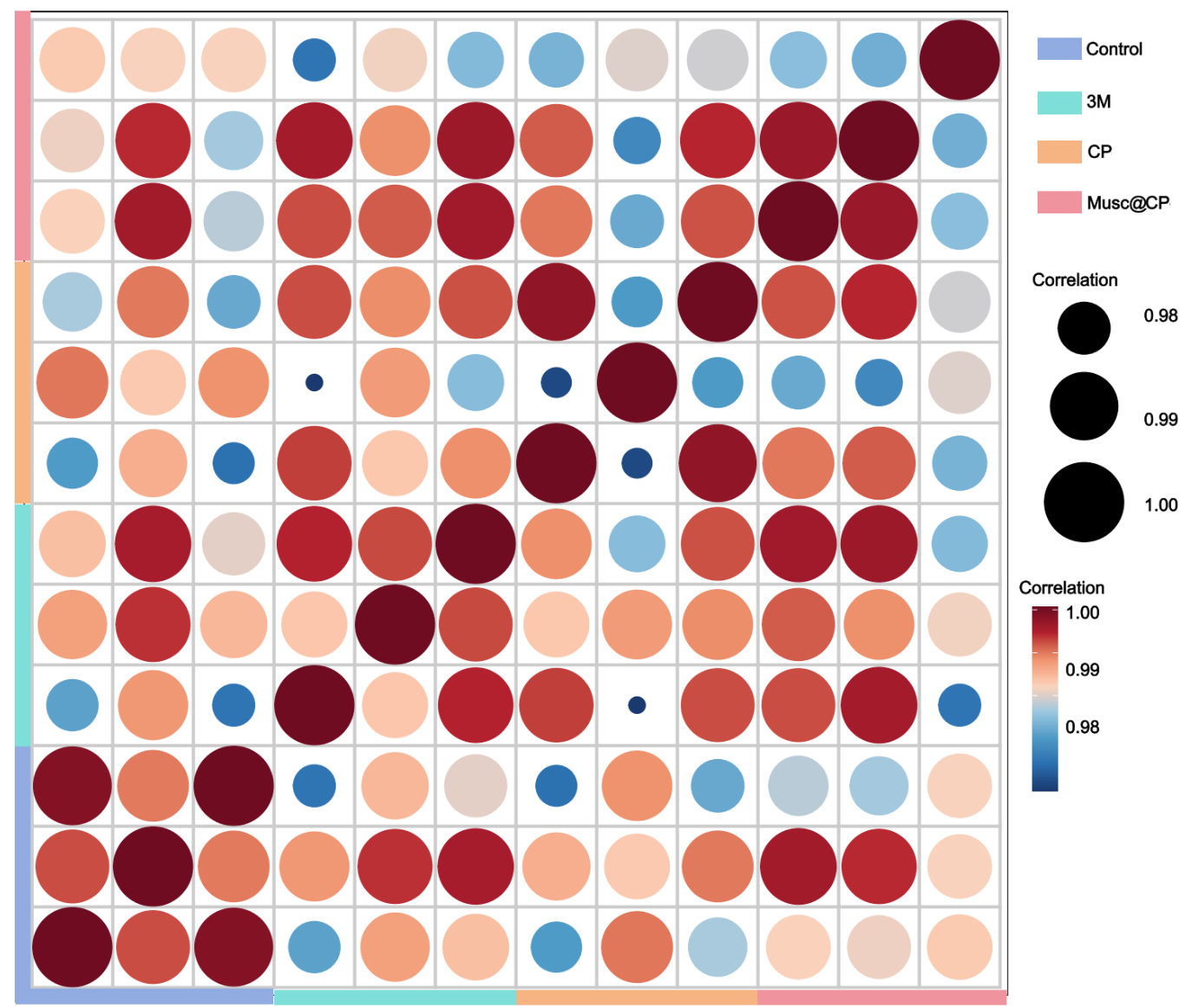


**Figure S22.** Correlation heatmap of each experimental group based on transcriptome FPKM results.


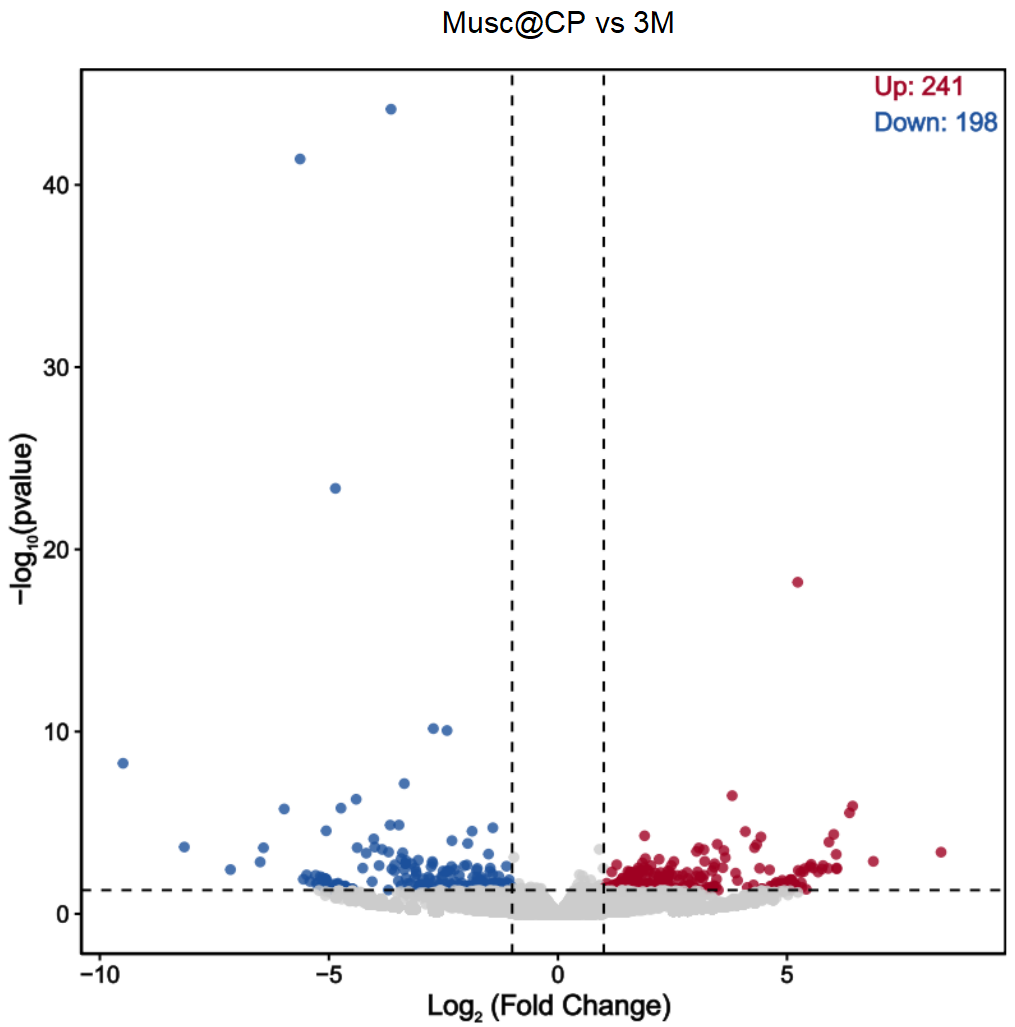


**Figure S23.** Volcano plot of DEGs between the Musc@CP and 3M groups.


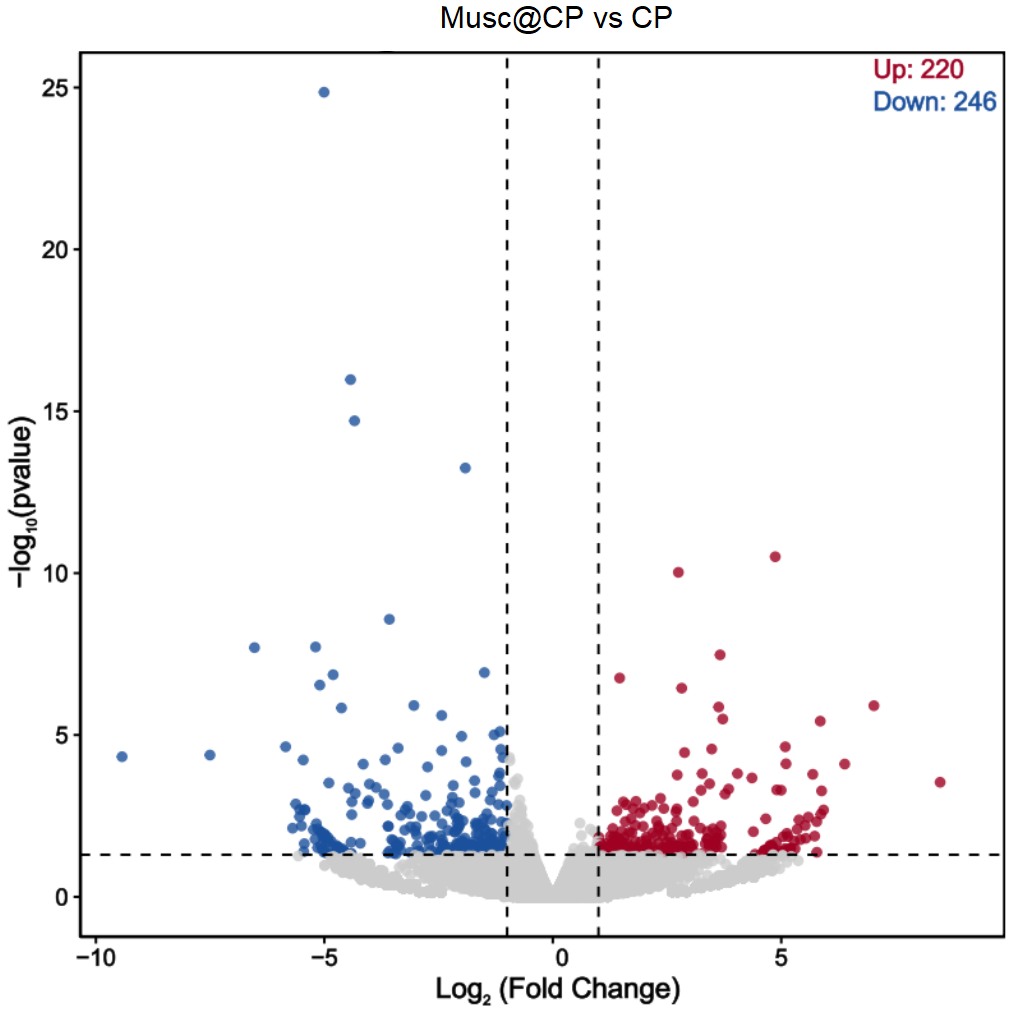


**Figure S24.** Volcano plot of DEGs between the Musc@CP and CP groups.


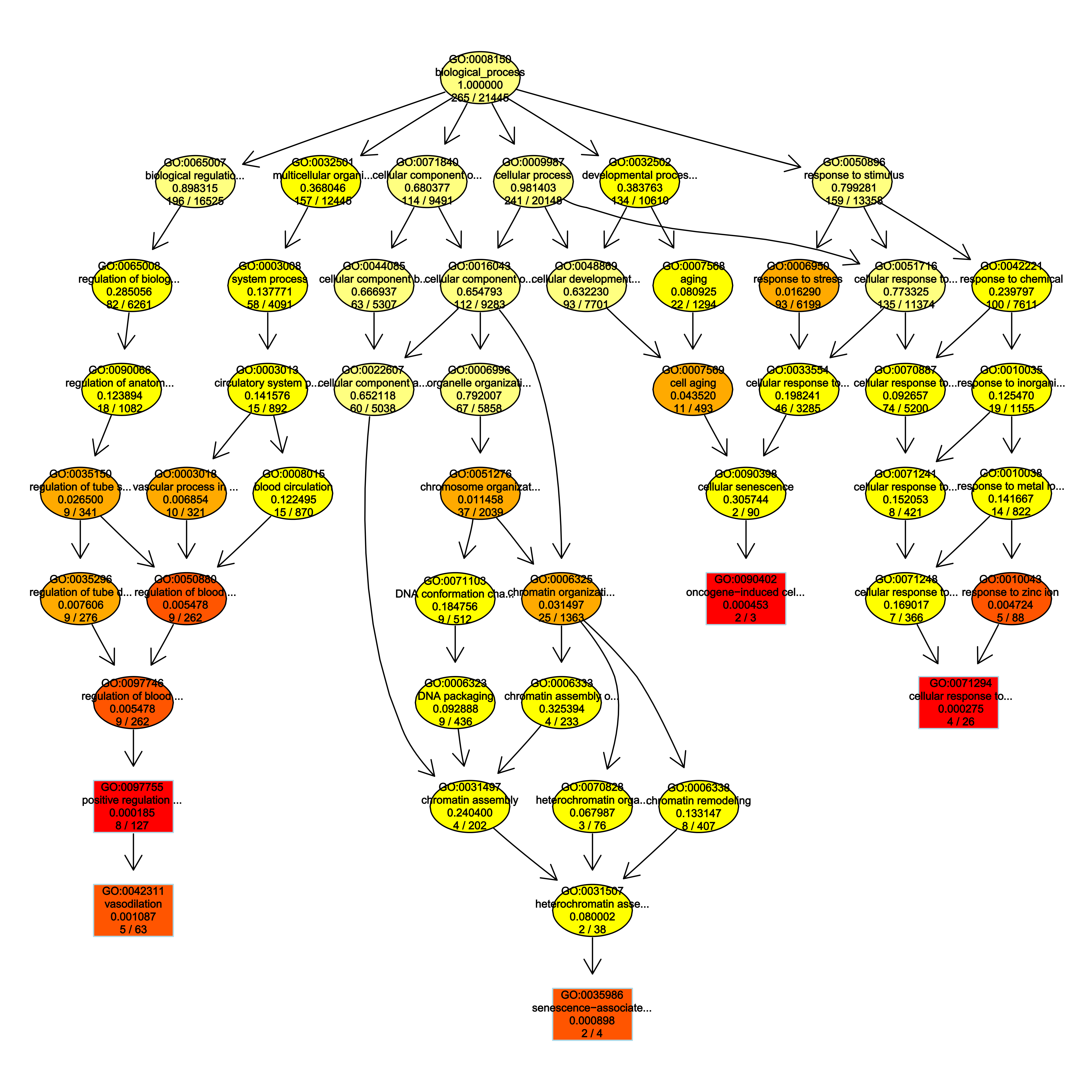


**Figure S25.** Network of GO biological process enrichment terms based on DEGs in Musc@CP group and Control group.


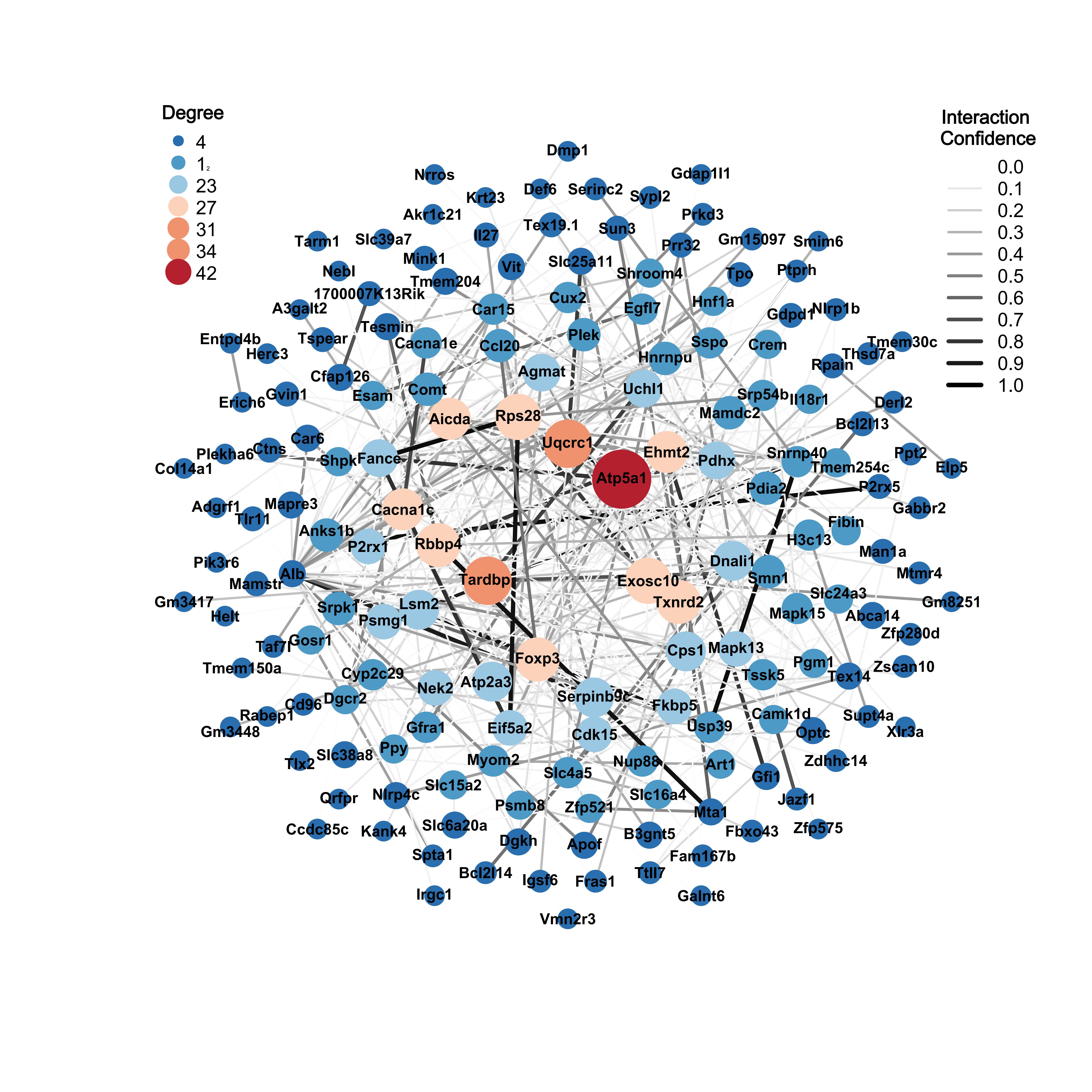


**Figure S26.** PPI network of DEGs between Musc@CP group and control group. (retrieved via STRING API in R with confidence score filtering).


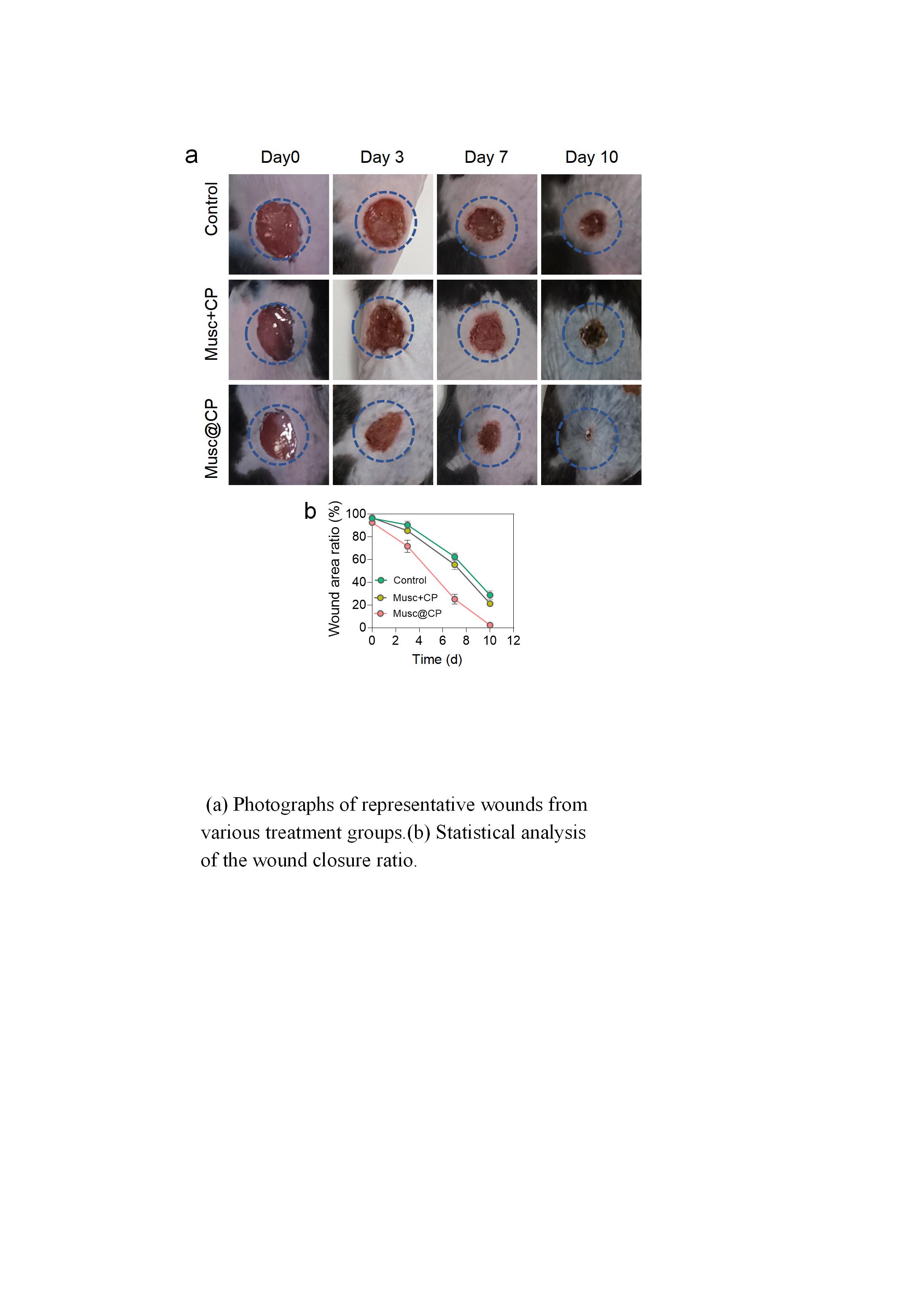


**Figure S27.** (a) Photographs of representative wounds from various treatment groups.(b) Statistical analysis of the wound closure ratio.

To distinguish between sustained release from nanofibers and simple physical mixing, we added a control group consisting of CP scaffold plus free muscone (Musc+CP) administered at the same total dose as loaded in Musc@CP.


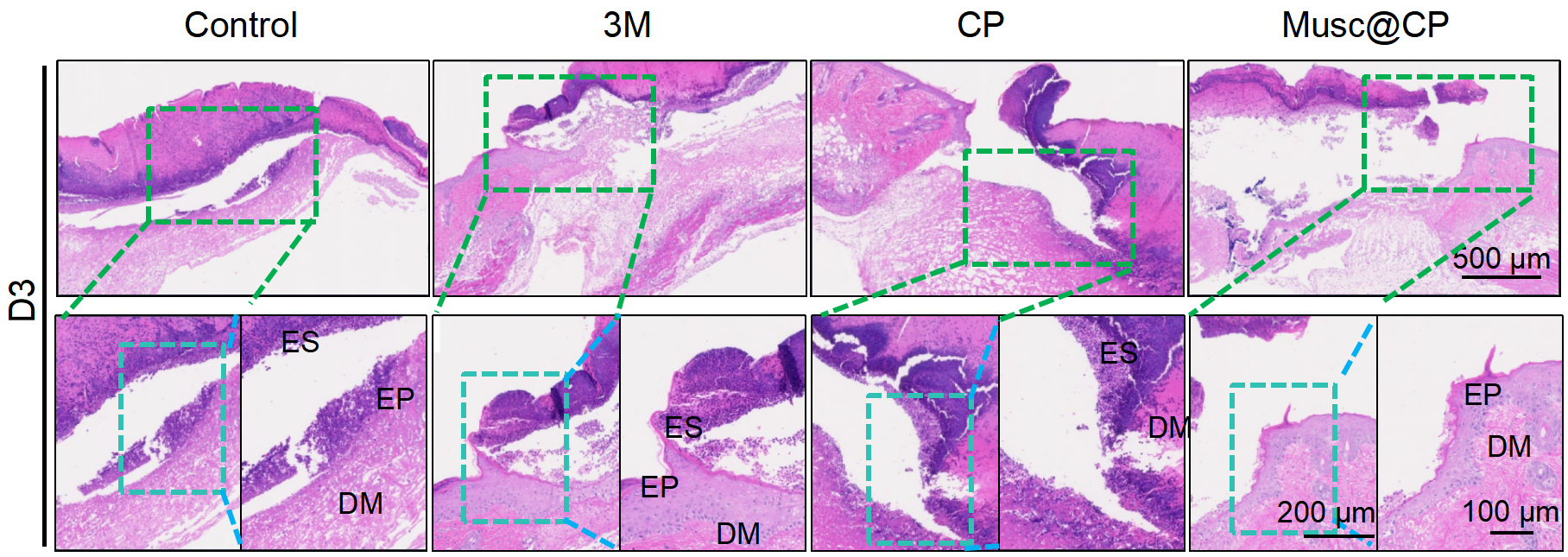


**Figure S28.** The H&E staining results of the wound tissue of diabetic mice with bacteria-free wounds on the 3rd day after dressing treatment. EP, DM and ES stand for epidermis, dermis and epidermal surface, respectively.


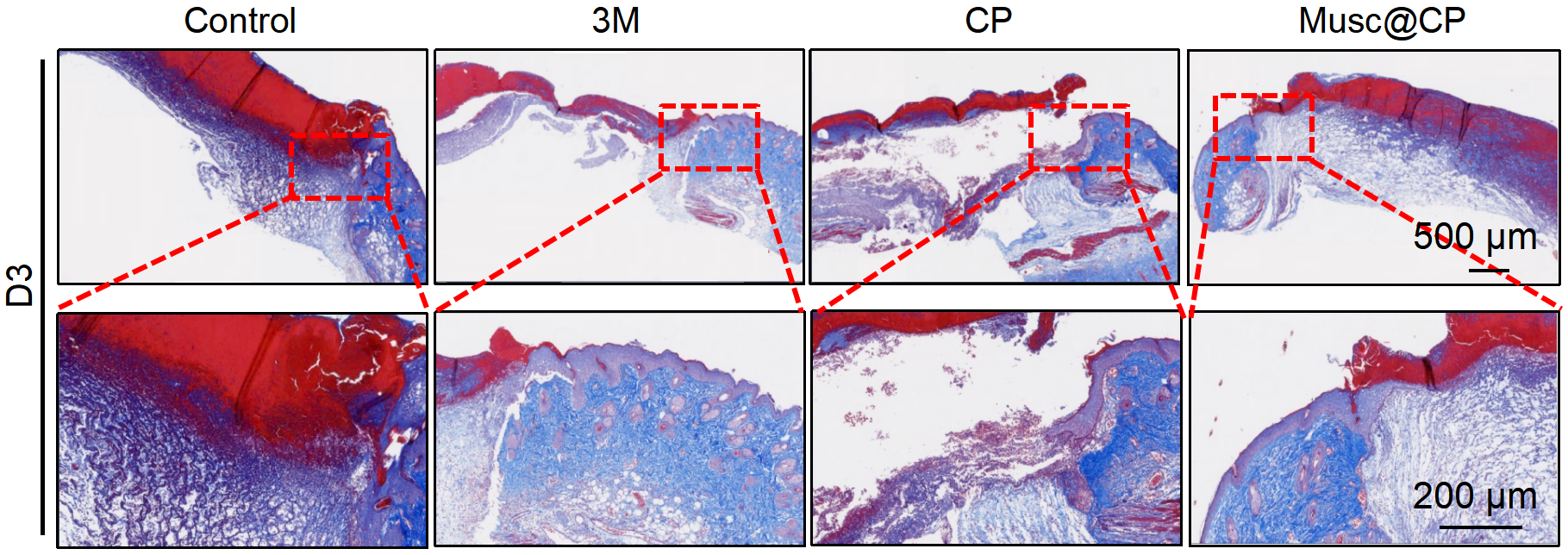


**Figure S29.** The Masson staining results of the wound tissue of diabetic mice with bacteria-free wounds on the 3rd day after dressing treatment.


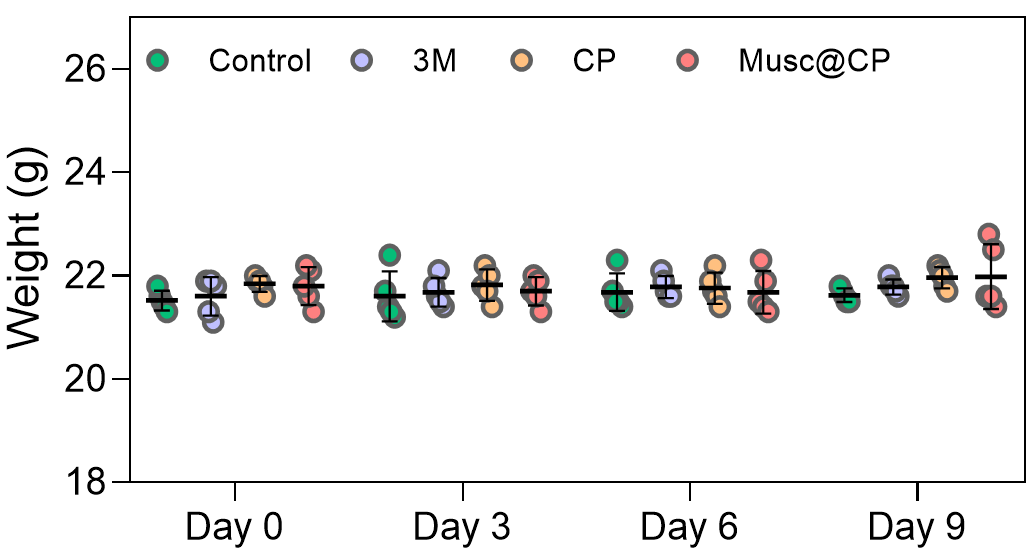


**Figure S30.** Weight of diabetic C57BL/6 mice with bacteria-free wounds during the whole wound healing experiment.


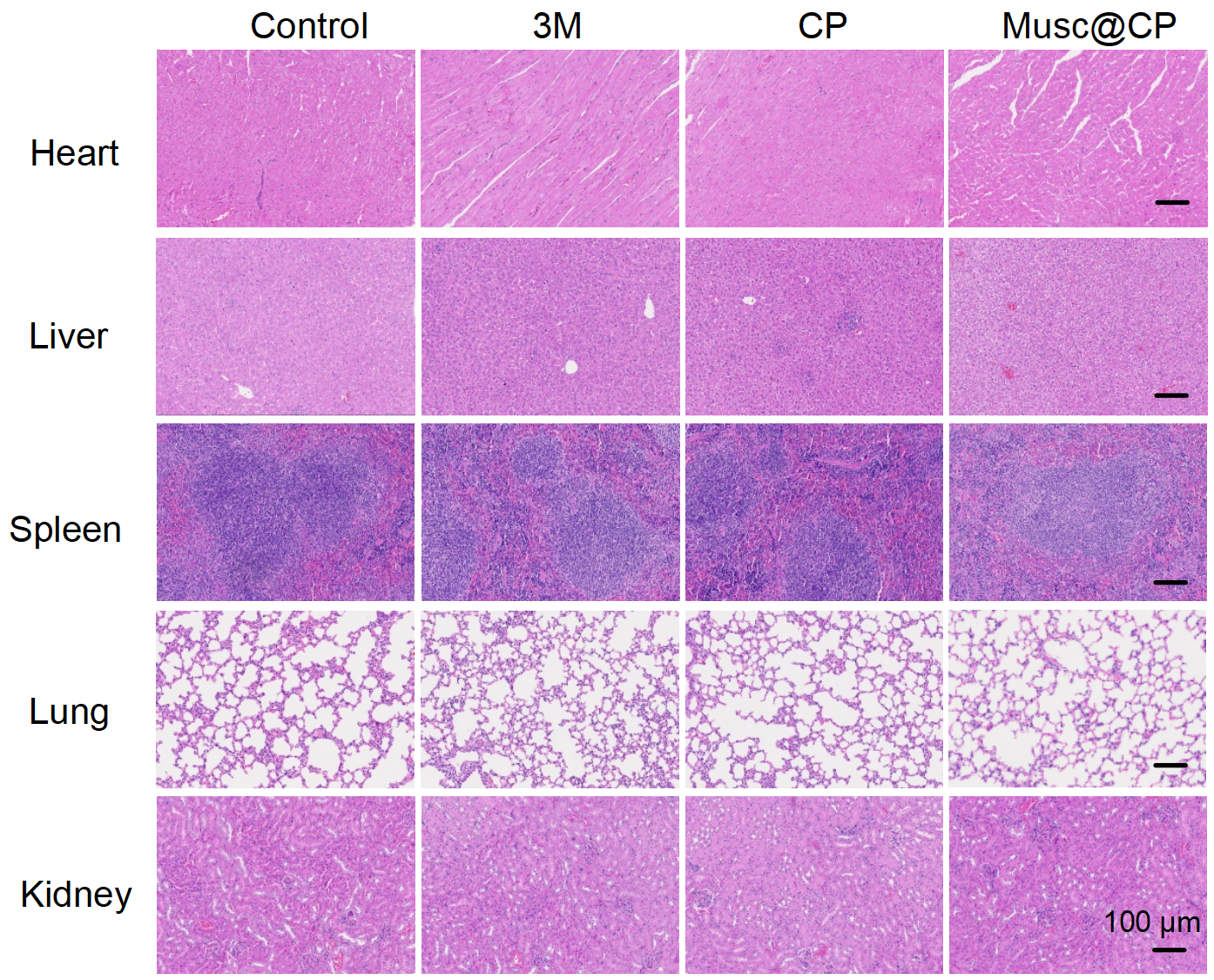


**Figure S31.** H&E staining images of heart, liver, spleen, lung and kidney of diabetic C57BL/6 mice with bacteria-free wounds under different treatments. Three times each experiment was repeated independently with similar results.

Histopathological examination of major organs revealed intact tissue architecture without apparent inflammatory infiltration, cellular degeneration, or necrosis in mice treated with Musc@CP compared to the control group.


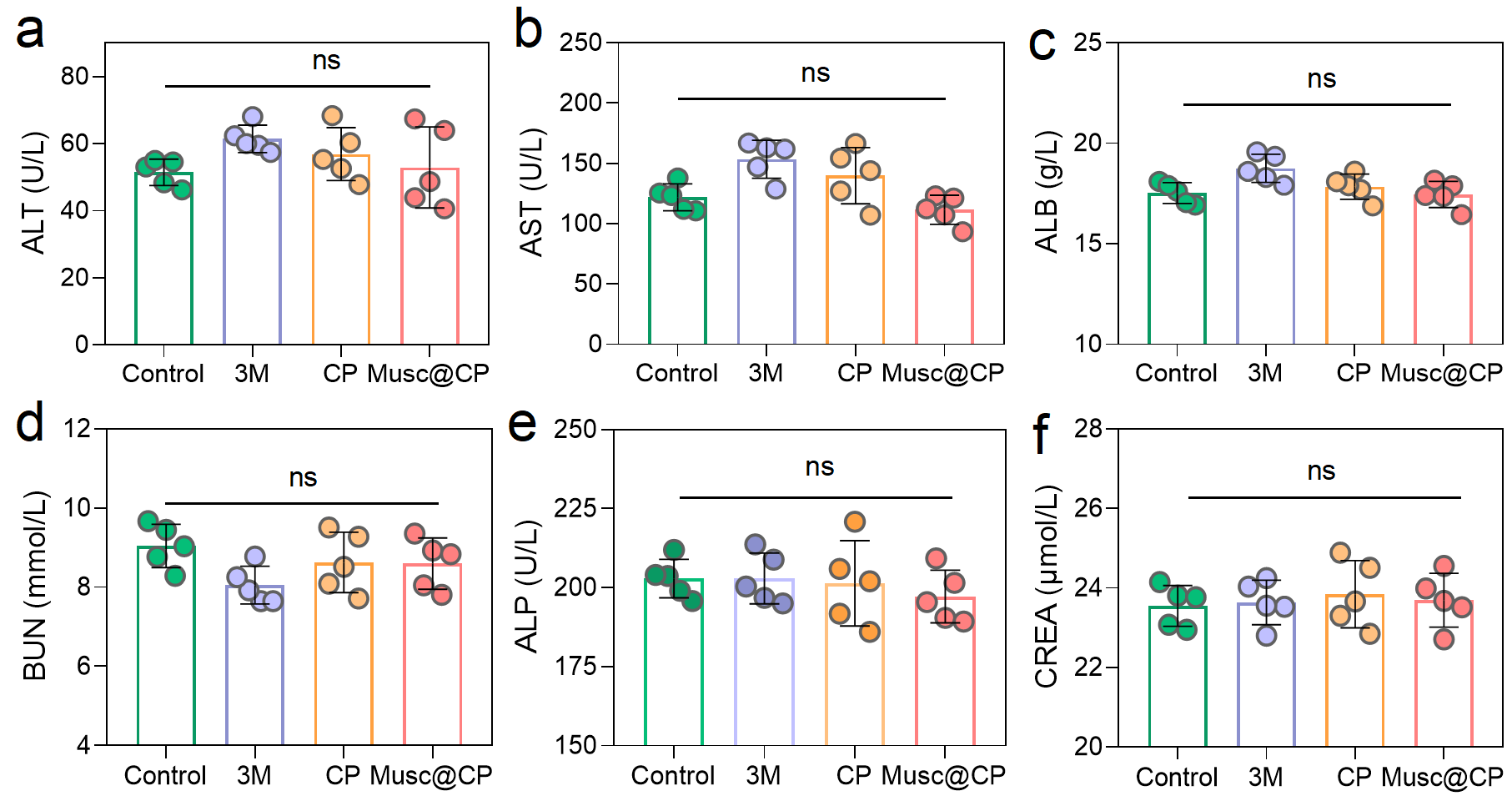


**Figure S32.** Results of biochemical tests of liver and kidney function in each group of diabetic C57BL/6 mice with bacteria-free wounds: (a) Alanine aminotransferase, ALT. (b) Aspartate aminotransferase, AST. (c) Albumin, ALB. (d) Blood urea nitrogen, BUN. (e) Alkaline phosphatase, ALP. (f) Creatinine, CREA. (n = 5)


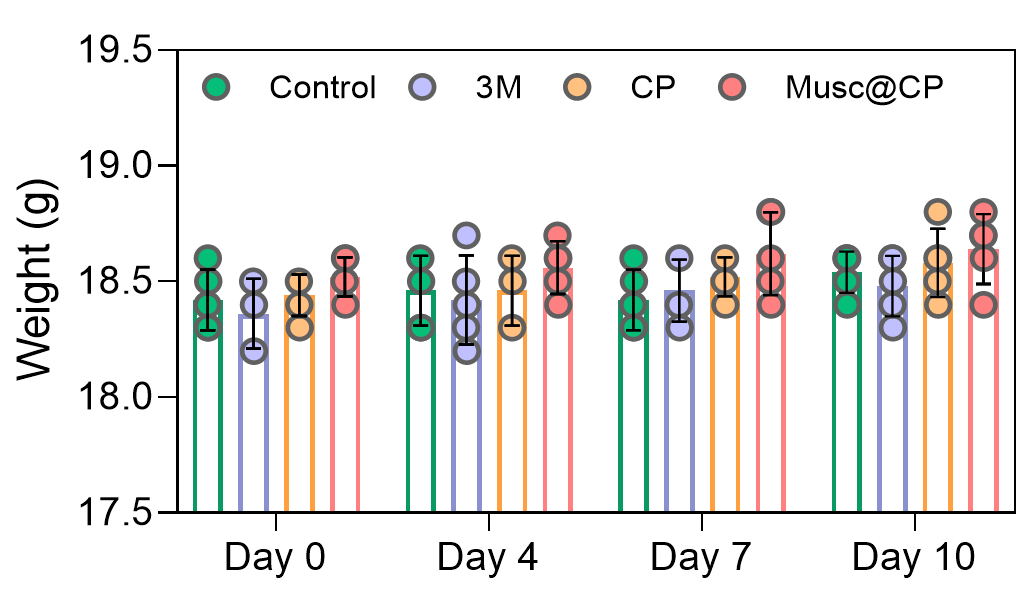


**Figure S33.** Weight of diabetic C57BL/6 mice with bacterially infected wounds during the whole wound healing experiment.

The body weights of diabetic mice showed no significant fluctuations (21.5 ± 0.7 g) during the study. This stability suggests the absence of systemic toxicity from Musc@CP treatment and demonstrates a favorable biosafety profile.


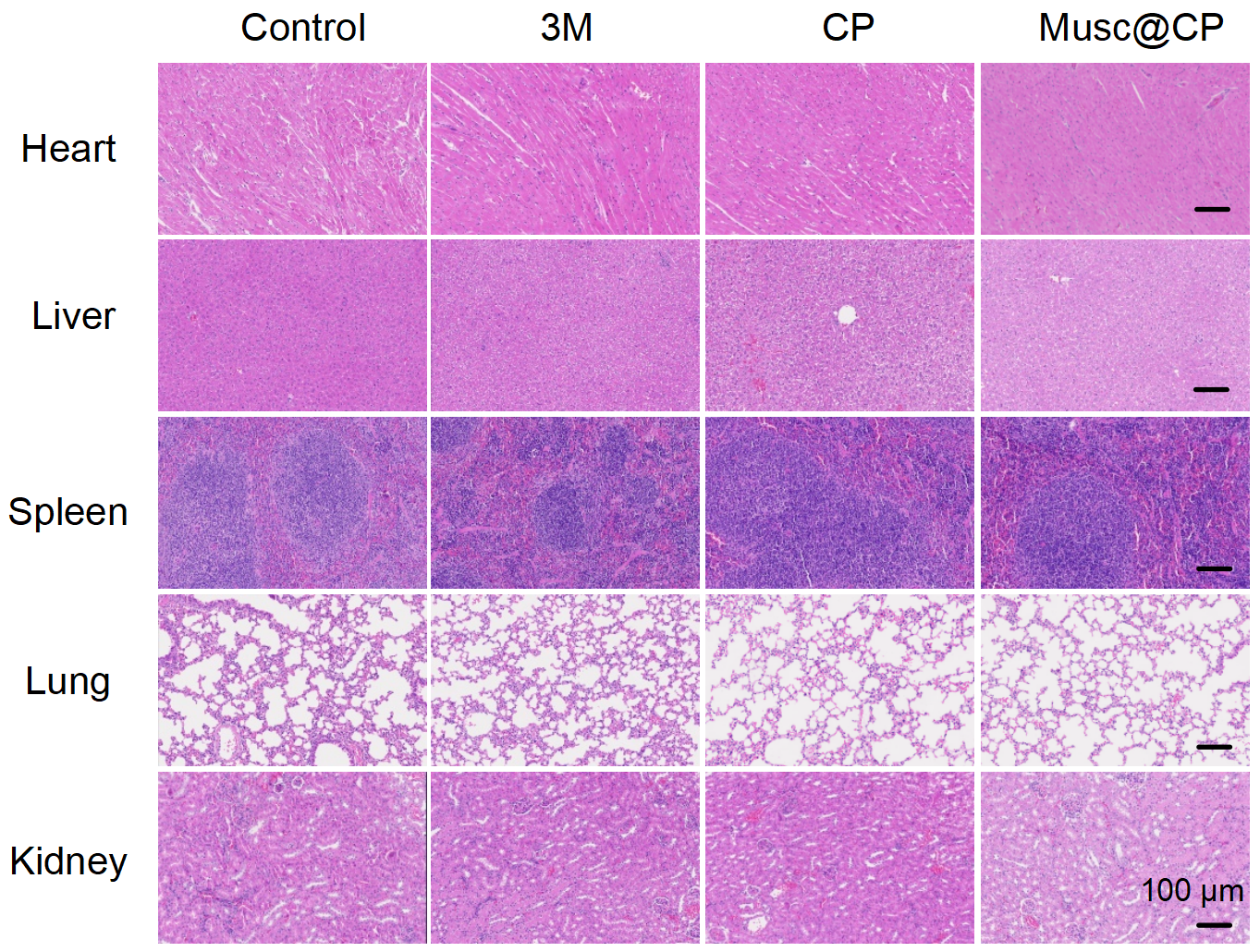


**Figure S34.** H&E staining images of heart, liver, spleen, lung and kidney of diabetic mice with bacterially infected wounds under different treatments. Three times each experiment was repeated independently with similar results.

Histopathological examination of major organs revealed intact tissue architecture without apparent inflammatory infiltration, cellular degeneration, or necrosis in mice treated with Musc@CP compared to the control group.


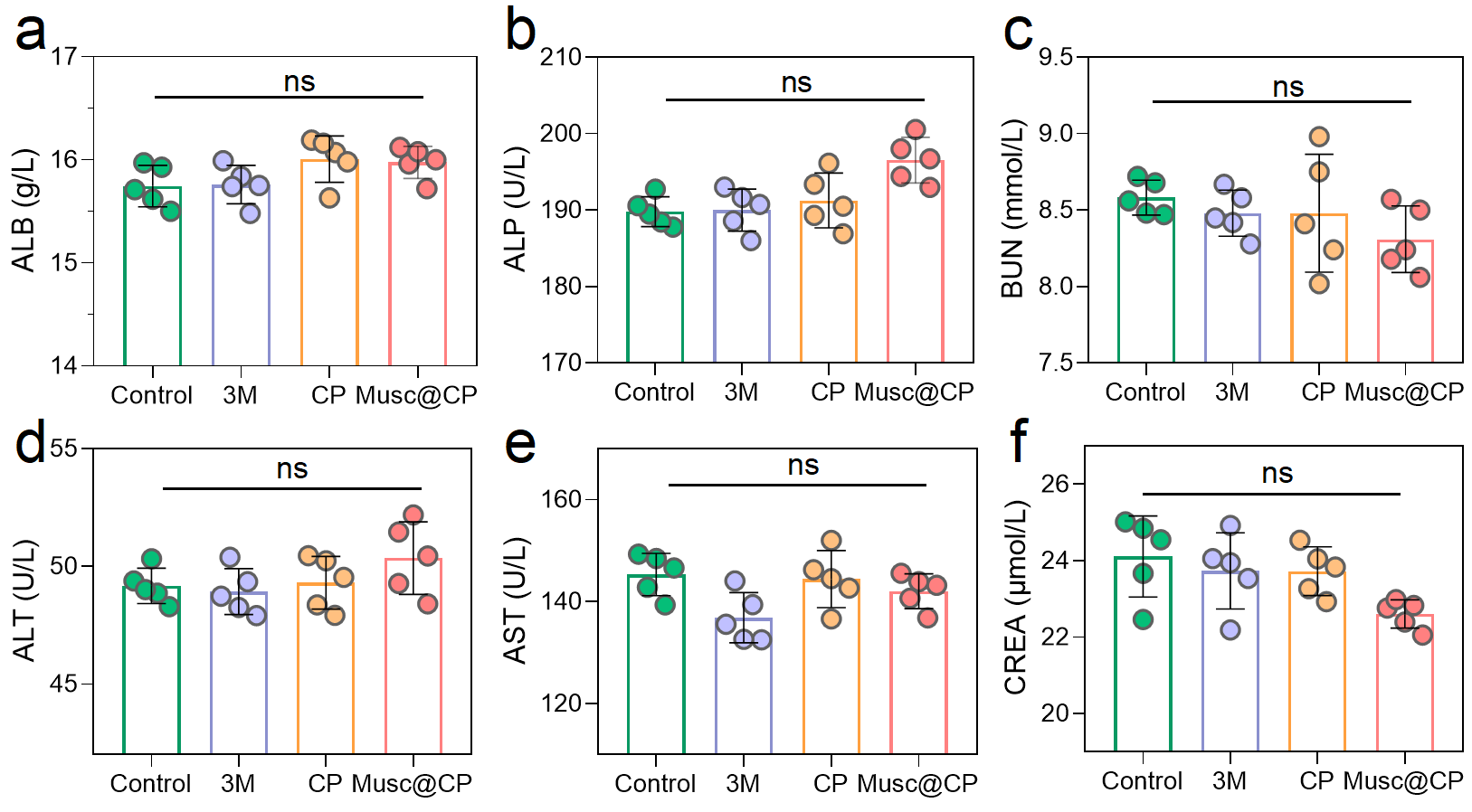


**Figure S35.** Results of biochemical tests of liver and kidney function in each group of diabetic C57BL/6 mice. (a) Albumin, ALB. (b) Alkaline phosphatase, ALP. (c) Blood urea nitrogen, BUN. (d) Alanine aminotransferase, ALT. (e) Aspartate aminotransferase, AST. (f) Creatinine, CREA. (n = 5).

No statistically significant differences were observed in key hepatic and renal function parameters including ALB, ALP, BUN, ALT, AST, and CREA in the Musc@CP group relative to controls. These collective findings indicate that Musc@CP treatment did not induce hepatotoxicity or renal dysfunction in vivo, further supporting its favorable biocompatibility and systemic safety as a wound dressing.

Table S1 Results of 9 orthogonal experiments.


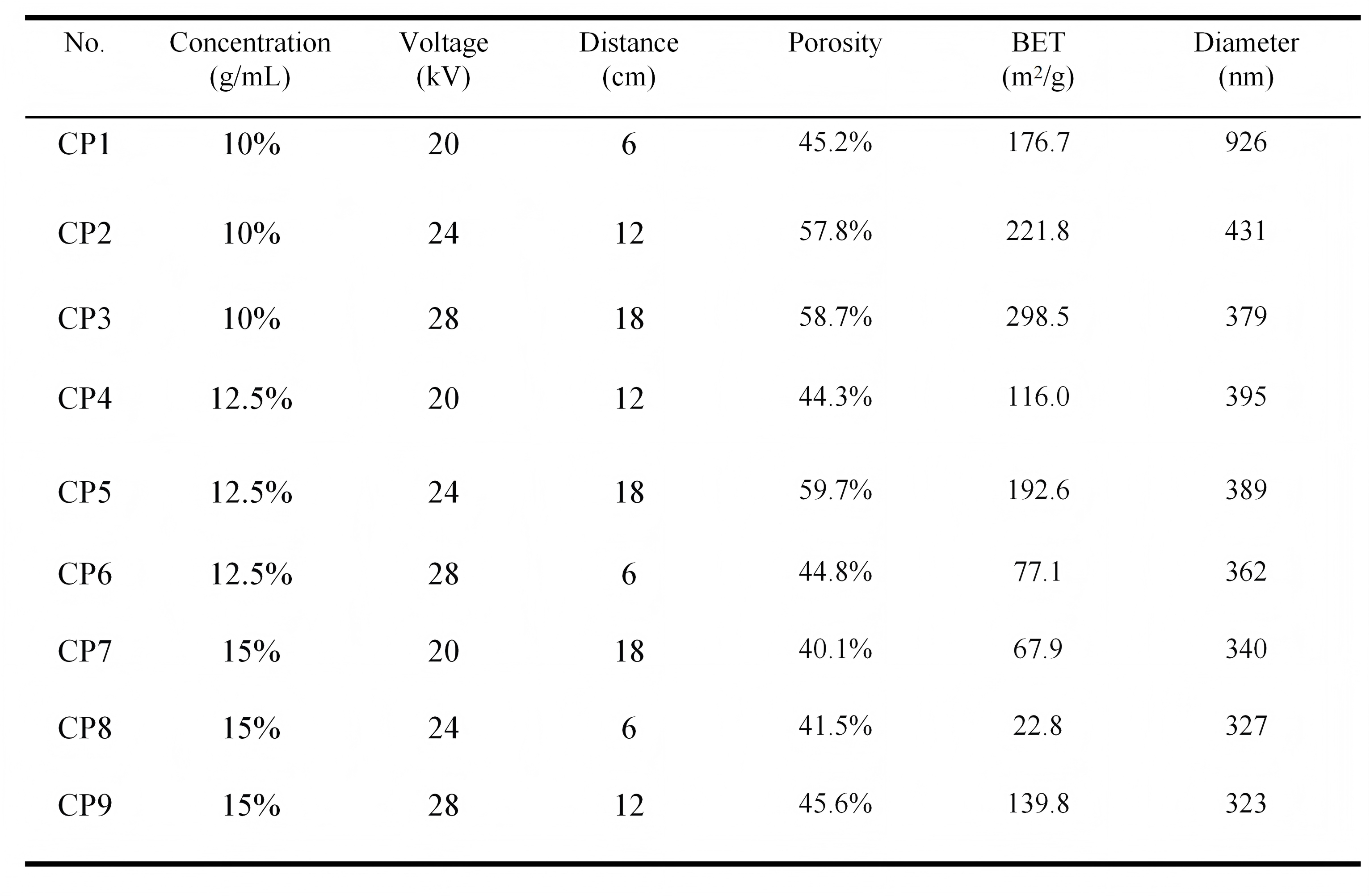


Table S2 Surface composition of different elements.

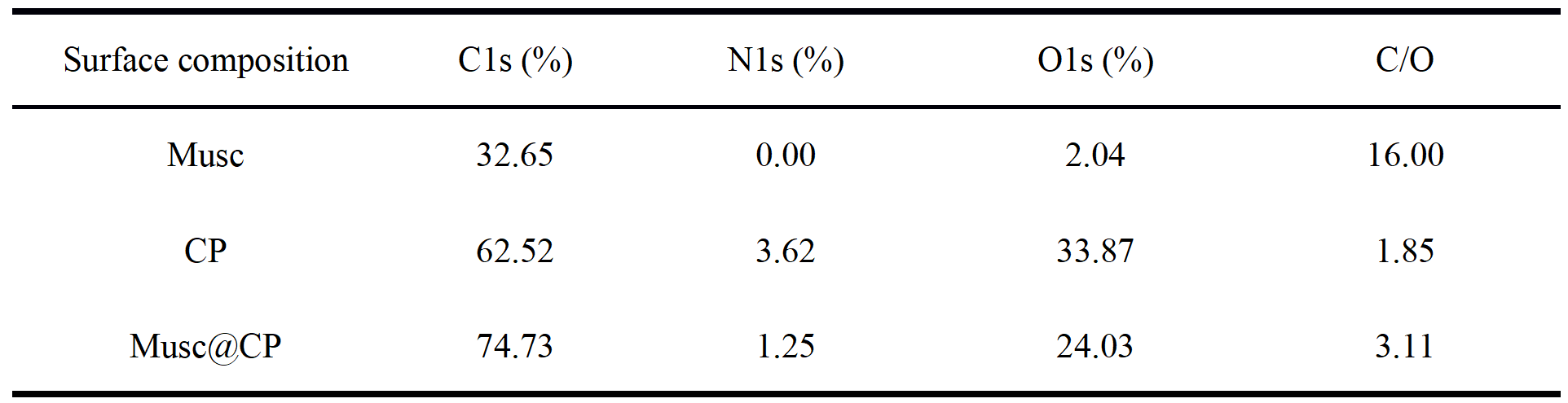

Supplement: Supplementary file 1 — Supporting File: advs76758‐sup‐0001‐SuppMat.docx. [file ADVS-9999-e76758-s001.docx]
